# Correction: *Randomised controlled trial to investigate the effectiveness of thoracic epidural and paravertebral blockade in reducing chronic post-thoracotomy pain (TOPIC): a pilot study to assess feasibility of a large multicentre trial*

**DOI:** 10.1136/bmjopen-2018-023679corr1

**Published:** 2019-08-15

Yeung J, Middleton L, Tryposkiadis K, *et al*. Randomised controlled trial to investigate the effectiveness of thoracic epidural and paravertebral blockade in reducing chronic post-thoracotomy pain (TOPIC): a pilot study to assess feasibility of a large multicentre trial. *BMJ Open* 2019;9:e023679. doi: 10.1136/bmjopen-2018-023679

This article was previously published with an error.

The first name of one of the authors is incorrect, Antony Worrall should be Andrew Worrall.

