# Supplementary material for: Randomised controlled trial to investigate the effectiveness of thoracic epidural and paravertebral blockade in reducing chronic post-thoracotomy pain (TOPIC): a pilot study to assess feasibility of a large multicentre trial
Source: BMJ Open. 2019 Jul 9;9(7):e023679. doi: 10.1136/bmjopen-2018-023679 (PMC6624049; doi:10.1136/bmjopen-2018-023679)
Supplement: Supplementary data [file bmjopen-2018-023679supp001.pdf]

TOPIC PILOT STUDY SUPPLEMENTARY APPENDIX

Table S1: Intraoperative data summary 2

Table S2: Analgesic use summary 3

Table S3: Post-operative (in hospital) pain scores summary 4

Table S4: Comprehensive summary of Visual Analogue Scale (VAS) scores 5

Table S5: Complications, short-term mortality and length of stay summary 7

Table S6: Patient satisfaction and blinding questions results summary 8

Table S1: Intraoperative data summary

		PVB (n=35)	TEB (n=33)[1]
Vasopressor/inotrope used		30 (86)	27 (82)
Delivery route	Boluses	24 (80)	24 (89)
	Infusion	3 (10)	1 (4)
	Both	3 (10)	2 (7)
Overall fluid therapy and balance	Median [IQR]	1000 [1000, 2000]	1000 [1000, 1500]
Grade of surgeon was consultant (as opposed to trainee)		21 (60)	18 (55)
Side of operation was left (as opposed to right)		9 (26)	10 (30)
Type of operation	Lobectomy	18 (51)	18 (55)
	Pneumonectomy	3 (9)	2 (6)
	Segmentectomy	1 (3)	1 (3)
	Wedge	3 (9)	2 (6)
	Lung biopsy	1 (3)	0 (-)
	Wedge and Segmentectomy	0 (-)	1 (3)
	Lobectomy and Wedge	1 (3)	0 (-)
	Lobectomy and Other	2 (6)	2 (6)
	Other	6 (17)	7 (21)
Surgical approach	Anterolateral	4 (12)[2]	2 (6)
	Posterolateral	22 (65)[2]	23 (70)
	Muscle sparing	0 (-)[2]	1 (3)
	Mini-thoracotomy	1 (3)[2]	0 (-)
	Posterolateral and Muscle sparing	7 (21)[2]	6 (18)
	Mini-limited lateral	0 (-)[2]	1 (3)

Frequency (%) presented (unless otherwise stated)
[1] one participant received neither technique as they did not have an operation; [2] one response missing (n=34)

Table S2: Analgesic use summary

			In Hospital (post-surgery)							
	Intraoperative		Day 1		Day 2		Day 3		Between day 3 and discharge/final assessment	
**Drug used**	**PVB**	**TEB**	**PVB**	**TEB**	**PVB**	**TEB**	**PVB**	**TEB**	**PVB**	**TEB**
Paracetamol	29	29	1	-	-	-	-	-	-	-
Diclofenac	3	-	3	2	-	-	-	-	-	-
Remifentanil	-	-	6	5	2	-	-	-	-	-
Fentanyl	-	-	16	11	5	4	2	-	-	-
Morphine	2	-	6	1	14	3	3	2	2	-
Diamorphine	-	2	-	10	1	7	-	1	-	-
Gabapentin	-	-	-	-	-	-	-	1	-	-
Alfentanil	-	-	1	2	3	10	1	1	-	-
Bupivicaine	-	-	-	-	-	-	-	1	-	-
Levobupivicaine	-	-	-	-	-	1	-	-	-	-
Pregabalin	-	-	-	-	-	-	1	-	-	-
Ketorolac	-	-	-	-	-	2	3	2	2	-

Number of participants administered drug indicated (total number given PVB=35 and total number given TEB=33)

Table S3: Post-operative (in hospital) pain scores summary

	PVB mean score (SD, n)	TEB mean score (SD, n)	Difference between groups (95% CI) [1]
Pain score at rest (0-3, higher=worse pain)			
Day 1	0.4 (0.7, 34)	0.5 (1.0, 33)	-0.1 (-0.5, 0.3)
Day 2	0.5 (0.7, 33)	0.7 (1.0, 32)	-0.2 (-0.6, 0.3)
Day 3	0.2 (0.4, 27)	0.6 (0.7, 27)	-0.4 (-0.8, -0.1)
Pain score at movement (0-3, higher=worse pain)			
Day 1	0.9 (0.9, 34)	0.8 (1.0, 33)	0.1 (-0.3, 0.6)
Day 2	1.2 (0.8, 33)	1.3 (0.9, 32)	-0.1 (-0.5, 0.4)
Day 3	0.8 (0.9, 27)	1.0 (1.0, 27)	-0.2 (-0.8, 0.3)

[1] scores<0 indicate less pain with PVB

Table S4: Comprehensive summary of Visual Analogue Scale (VAS) scores

	PVB mean score (SD, n)	TEB mean score (SD, n)	Difference between groups (95% CI) [1]
Average chest pain overall (0-10, higher=worse pain)			
Baseline	0.6 (1.8, 32)	0.7 (1.7, 33)	-1.0 (-1.0, 0.8)
24 hours post-surgery	5.2 (2.5, 33)	3.7 (3.4, 32)	1.5 (-0.02, 2.9)
48 hours post-surgery	6.4 (1.8, 31)	5.5 (2.7, 31)	0.9 (-0.3, 2.1)
72 hours post-surgery	5.1 (25, 2.4)	6.1 (2.5, 26)	-1.0 (-2.4, 0.3)
Discharge	4.9 (1.9, 29)	4.3 (2.5, 32)	0.6 (-0.5, 1.7)
3 months	2.1 (1.8, 25)	2.0 (2.6, 21)	0.1 (-1.3, 1.4)
**6 months**	0.8 (1.2, 27)	1.7 (2.1, 25)	-0.9 (-1.8, 0.1)
Average chest pain at rest (0-10, higher=worse pain)			
Baseline	0.4 (1.4, 32)	0.7 (1.7, 33)	-0.3 (-0.9, 0.5)
24 hours post-surgery	3.9 (2.2, 33)	3.3 (3.3, 32)	0.6 (-0.8, 2.0)
48 hours post-surgery	3.9 (2.1, 31)	4.3 (2.8, 31)	-0.4 (-1.6, 0.9)
72 hours post-surgery	3.2 (25, 2.6)	4.8 (2.8, 25)	-1.6 (-3.2, -0.1)
Discharge	3.1 (2.2, 29)	3.2 (2.5, 32)	-0.1 (-1.3, 1.1)
3 months	1.3 (1.4, 25)	1.5 (2.2, 21)	-0.2 (-1.3, 0.9)
**6 months**	0.4 (0.7, 27)	1.2 (1.7, 25)	-0.8 (-1.6, -0.1)
Average chest pain after coughing (0-10, higher=worse pain)			
Baseline	0.7 (1.9, 32)	0.9 (1.9, 33)	-0.2 (-1.2, 0.7)
24 hours post-surgery	6.2 (2.4, 33)	4.7 (3.3, 32)	1.5 (0.04, 2.9)
48 hours post-surgery	6.4 (2.5, 31)	6.1 (2.6, 31)	0.3 (-1.0, 1.5)
72 hours post-surgery	6.3 (2.6, 25)	7.0 (2.5, 26)	-0.7 (-2.2, 0.7)
Discharge	5.8 (2.2, 29)	5.5 (2.8, 32)	0.4 (-0.9, 1.7)
3 months	2.0 (1.8, 25)	1.95 (2.7, 21)	0.05 (-1.4, 1.4)
**6 months**	1.5 (1.9, 27)	2.1 (2.5, 24)	-0.6 (-1.8, 0.7)
Average chest pain after/with moving (0-10, higher=worse pain)			
Baseline	0.6 (1.9, 32)	0.8 (1.8, 33)	-0.2 (-1.1, 0.7)
24 hours post-surgery	6.1 (2.4, 33)	4.2 (3.3, 32)	1.9 (0.4, 3.3)
48 hours post-surgery	5.9 (2.3, 32)	5.4 (3.0, 31)	0.5 (-0.8, 1.9)
72 hours post-surgery	5.0 (2.6, 24)	6.3 (2.6, 25)	-1.3 (-2.8, 0.2)
Discharge	4.9 (2.2, 31)	4.5 (2.7, 31)	0.4 (-0.9, 1.6)
3 months	1.9 (1.8, 24)	2.2 (3.0, 23)	-0.3 (-1.8, 1.1)
**6 months**	1.0 (1.6, 27)	2.0 (2.7, 26)	-1.0 (-2.2, 0.3)
Average chest pain after physiotherapy (0-10, higher=worse pain)			
Baseline	0.04 (0.1, 29)	0.2 (0.5, 28)	-0.16 (-1.1, 0.7)
24 hours post-surgery	5.4 (2.3, 31)	4.1 (3.2, 30)	1.3 (-0.2, 2.7)
48 hours post-surgery	5.6 (2.3, 32)	5.0 (2.8, 31)	0.6 (-0.8, 1.9)
72 hours post-surgery	4.9 (2.4, 24)	5.9 (2.8, 25)	-1.0 (-2.5, 0.5)
Discharge	4.8 (2.1, 30)	3.8 (2.6 ,30)	1.0 (-0.2, 2.3)
3 months	1.95 (2.0, 18)	2.0 (2.9, 19)	-0.05 (-1.7, 1.6)
**6 months**	1.1 (1.6, 21)	2.0 (2.9, 19)	-0.9 (-2.3, 0.7)
Worst chest pain overall (0-10, higher=worse pain)			
Baseline	0.7 (1.7, 33)	0.9 (2.1, 33)	-0.2 (-1.2, 0.7)
24 hours post-surgery	7.7 (2.0, 33)	6.0 (3.5, 32)	1.7 (0.3, 3.2)
48 hours post-surgery	8.2 (1.6, 32)	7.4 (2.5, 31)	0.8 (-0.3, 1.9)
72 hours post-surgery	7.5 (2.0, 24)	7.9 (2.1, 25)	-0.4 (-1.6, 0.7)
Discharge	7.2 (2.1, 31)	6.6 (2.9, 31)	0.6 (-0.7, 1.9)
3 months	3.5 (2.9, 24)	2.7 (3.4, 23)	0.8 (-1.1, 2.7)
**6 months**	1.9 (2.5, 27)	3.1 (3.4, 26)	-1.2 (-2.8, 0.5)
Worst chest pain at rest (0-10, higher=worse pain)			
Baseline	0.3 (1.2, 33)	0.7 (1.7, 33)	-0.4 (-1.1, 0.4)
24 hours post-surgery	5.7 (2.8, 32)	4.8 (3.6, 32)	0.9 (-0.7, 2.5)
48 hours post-surgery	5.5 (2.7, 32)	5.4 (3.1, 31)	0.1 (-1.4, 1.6)
72 hours post-surgery	4.6 (2.8, 24)	6.1 (3.0, 25)	-1.5 (-3.2, 0.1)
Discharge	4.3 (2.5, 31)	4.7 (2.7, 31)	-0.4 (-1.7, 0.9)
3 months	2.2 (2.3, 24)	1.9 (2.7, 23)	0.3 (-1.2, 1.7)

**6 months**	1.0 (1.6, 27)	1.9 (2.7, 26)	-0.9 (-2.1, 0.3)
Worst chest pain after coughing (0-10, higher=worse pain)			
Baseline	0.6 (1.6, 33)	1.0 (2.2, 33)	-0.4 (-1.4, 0.6)
24 hours post-surgery	7.2 (1.9, 33)	5.5 (3.4, 32)	1.7 (0.3, 3.1)
48 hours post-surgery	7.6 (2.4, 32)	7.0 (2.5, 31)	0.6 (-0.7, 1.7)
72 hours post-surgery	7.2 (2.5, 25)	7.8 (2.1, 26)	-0.6 (-1.9, 0.7)
Discharge	6.7 (2.3, 30)	5.9 (2.9, 32)	0.8 (-0.5, 2.1)
3 months	2.9 (2.5, 25)	2.8 (3.1, 22)	0.1 (-1.7, 1.7)
**6 months**	1.8 (2.2, 28)	2.5 (2.9, 26)	-0.6 (-2.1, 0.8)
Worst chest pain after/with moving (0-10, higher=worse pain)			
Baseline	0.5 (1.5, 33)	0.9 (1.9, 33)	-0.4 (-1.2, 0.5)
24 hours post-surgery	7.2 (2.1, 33)	5.4 (3.6, 32)	1.8 (0.3, 3.3)
48 hours post-surgery	7.3 (1.9, 32)	6.5 (2.6, 31)	0.8 (-0.3, 2.1)
72 hours post-surgery	6.3 (2.3, 25)	7.0 (2.6, 26)	-0.7 (-2.1, 0.7)
Discharge	6.4 (2.3, 30)	5.8 (2.7, 32)	0.6 (-0.6, 1.9)
3 months	2.3 (1.8, 25)	2.5 (2.8, 22)	-0.2 (-1.6, 1.2)
**6 months**	1.3 (1.8, 28)	2.5 (3.2, 26)	-1.1 (-2.6, 0.4)
Worst chest pain after physiotherapy (0-10, higher=worse pain)			
Baseline	0.1 (0.2, 30)	0.2 (0.6, 29)	-0.1 (-0.4, 0.1)
24 hours post-surgery	6.2 (2.3, 31)	5.0 (3.5, 30)	1.2 (-0.4, 2.7)
48 hours post-surgery	6.5 (2.6, 31)	6.0 (2.8, 31)	0.6 (-0.8, 1.9)
72 hours post-surgery	5.9 (2.6, 25)	6.5 (2.6, 26)	-0.6 (-2.1, 0.8)
Discharge	6.0 (2.3, 29)	4.8 (2.8, 31)	1.2 (-0.1, 2.5)
3 months	2.6 (1.8, 25)	1.9 (2.7, 17)	0.7 (-0.9, 2.3)
**6 months**	1.2 (1.7, 22)	1.8 (2.8, 18)	-0.6 (-2.2, 1.0)

[1] scores<0 indicate less pain with PVB

Table S5: Complications, short-term mortality and length of stay summary

	Day 1		Day 2		Day 3		Between day 3 and discharge/final assessment	
	PVB	TEB	PVB	TEB	PVB	TEB	PVB	TEB
Arrhythmias	1/34 (3)	2/33 (6)	2/32 (6)	4/32 (13)	1/26 (4)	1/27 (4)	1/34 (3)	3/32 (9)
Systolic blood pressure (<90mmHg)	2/35 (6)	8/33 (24)	2/33 (6)	2/32 (6)	2/27 (7)	2/27 (7)	0/34 (-)	0/32 (-)
Low Respiratory rate (<10/minute)	1/35 (3)	0/33 (-)	0/33 (-)	0/32 (-)	0/27 (-)	0/27 (-)	0/34 (-)	0/32 (-)
Drowsiness	6/34 (18)	4/33 (12)	3/32 (9)	2/32 (6)	0/26 (-)	2/27 (7)	0/34 (-)	1/32 (3)
Nausea & vomiting	8/35 (23)	11/33 (33)	4/33 (12)	4/32 (13)	2/26 (8)	3/27 (11)	5/34 (15)	5/32 (16)
Urinary retention	0/35 (-)	0/33 (-)	1/33 (3)	0/32 (-)	0/27 (-)	0/27 (-)	1/34 (3)	1/32 (3)
Itching	0/34	1/33 (3)	0/32	1/32 (3)	0/26	0/27	0/32	1/32 (3)
High block (above T4)	0/35 (-)	0/33 (-)	0/33 (-)	0/32 (-)	0/27 (-)	0/27 (-)	0/34 (-)	0/32 (-)
Post-dural puncture headache	0/35 (-)	0/33 (-)	0/33 (-)	0/32 (-)	0/27 (-)	0/27 (-)	1/34 (3)	0/32 (-)
Post-op pulmonary complications (PPC)	2/21 (10)	1/19 (5)	2/19 (10)	1/18 (6)	1/15 (7)	1/15 (7)	0/19 (-)	0/19 (-)
Other	20/34 (59)	13/33 (39)	6/32 (19)	7/31 (23)	4/26 (15)	6/27 (22)	3/34 (9)	9/32 (28)
**Level 2 admission**	0/35 (-)	2/33 (6)	-	-	-	-	0/34 (-)	1/32 (3)
If yes, cardiovascular support	-	2/2 (100)	-	-	-	-	-	-
If yes, respiratory support	-	2/2 (100)	-	-	-	-	-	-
If yes, organ support	-	-	-	-	-	-	-	1/1 (100)
If yes, total length of stay *(Median [IQR], n)*		9 [6, 12]						6 [-]
**Level 3 admission**	1/35 (3)	1/32 (3)						
If yes, cardiovascular support	1/1 (100)	1/1 (100)	-	-	-	-	-	-
If yes, respiratory support	1/1 (100)	1/1 (100)	-	-	-	-	-	-
If yes, renal support	0/1 (-)	0/1 (-)	-	-	-	-	-	-
If yes, total length of stay *(Median [IQR], n)*	1 [-]	5 [-]	-	-	-	-	-	-
Discharge with flutter bag	-	-	-	-	-	-	5/34 (15)	4/33 (12)
Mortality	-	-	-	-	-	-	1/35 (3)	1/33 (3)

Frequency (%) presented (unless otherwise stated)

Table S6: Patient satisfaction and blinding questions results summary

		PVB	TEB
Satisfaction with pain therapy after surgery			
Discharge (n=31 for PVB, n=32 for TEB)	Very satisfied	18 (58)	20 (63)
	Satisfied	10 (33)	9 (28)
	Dissatisfied	2 (6)	2 (6)
	Very dissatisfied	1 (3)	1 (3)
3 months (n=26 for PVB, n=23 for TEB)	Very satisfied	13 (50)	9 (39)
	Satisfied	13 (50)	8 (35)
	Dissatisfied	0 (-)	6 (26)
	Very dissatisfied	0 (-)	0 (-)
6 months (n=26 for PVB, n=25 for TEB)	Very satisfied	14 (54)	16 (64)
	Satisfied	11 (42)	6 (24)
	Dissatisfied	0 (-)	2 (8)
	Very dissatisfied	1 (4)	1 (4)
Satisfaction with care provided by hospital			
Discharge (n=31 for PVB, n=32 for TEB)	Very satisfied	22 (71)	22 (69)
	Satisfied	9 (29)	8 (25)
	Dissatisfied	0 (-)	2 (6)
	Very dissatisfied	0 (-)	0 (-)
3 months (n=26 for PVB, n=23 for TEB)	Very satisfied	15 (58)	12 (52)
	Satisfied	11 (42)	9 (39)
	Dissatisfied	0 (-)	2 (9)
	Very dissatisfied	0 (-)	0 (-)
6 months (n=26 for PVB, n=26 for TEB)	Very satisfied	19 (73)	15 (58)
	Satisfied	5 (19)	9 (35)
	Dissatisfied	2 (8)	2 (8)
	Very dissatisfied	0 (-)	0 (-)
Do you know which technique you received?			
Discharge (n=31 for PVB, n=32 for TEB)	TEB	1 (3)	19 (60)
	PVB	8 (26)	3 (9)
	Don't know	22 (71)	10 (31)
3 months (n=26 for PVB, n=22 for TEB)	TEB	2 (8)	12 (55)
	PVB	2 (8)	0 (-)
	Don't know	22 (84)	10 (45)
6 months (n=26 for PVB, n=25 for TEB)	TEB	2 (8)	13 (52)
	PVB	5 (19)	1 (4)
	Don't know	19 (73)	11 (44)

Frequency (%) presented (unless otherwise stated)
