# Supplementary material for: Randomised controlled trial to investigate the effectiveness of thoracic epidural and paravertebral blockade in reducing chronic post-thoracotomy pain (TOPIC): a pilot study to assess feasibility of a large multicentre trial
Source: BMJ Open. 2019 Jul 9;9(7):e023679. doi: 10.1136/bmjopen-2018-023679 (PMC6624049; doi:10.1136/bmjopen-2018-023679)
Supplement: Author's manuscript [file bmjopen-2018-023679.draft_revisions.pdf]

BMJ Open

BMJ Open is committed to open peer review. As part of this commitment we make the peer review history of every article we publish publicly available.

When an article is published we post the peer reviewers' comments and the authors' responses online. We also post the versions of the paper that were used during peer review. These are the versions that the peer review comments apply to.

The versions of the paper that follow are the versions that were submitted during the peer review process. They are not the versions of record or the final published versions. They should not be cited or distributed as the published version of this manuscript.

BMJ Open is an open access journal and the full, final, typeset and author-corrected version of record of the manuscript is available on our site with no access controls, subscription charges or pay-per-view fees (http://bmjopen.bmj.com).

If you have any questions on BMJ Open's open peer review process please email info.bmjopen@bmj.com

BMJ Open

A RANDOMISED CONTROLLED TRIAL TO INVESTIGATE THE EFFECTIVENESS OF THORACIC EPIDURAL AND PARAVERTEBRAL BLOCKADE IN REDUCING CHRONIC POST-THORACOTOMY PAIN (TOPIC) – A PILOT STUDY TO ASSESS FEASIBILITY OF A LARGE MULTI-CENTRE TRIAL

Journal:	*BMJ Open*
Manuscript ID	bmjopen-2018-023679
Article Type:	Research
Date Submitted by the Author:	19-Apr-2018

Complete List of Authors:	Yeung, Joyce ; University of Warwick, Warwick Medical School; Heart of England NHS Foundation Trust, Department of Anaesthesia<br>Middleton, Lee; University of Birmingham, School of Health and Population Sciences<br>Tryposkiadis, Kostas; University of Birmingham, Birmingham Clinical Trials Unit<br>Kerr, Amy; Heart of England NHS Foundation Trust, Department of Thoracic Surgery<br>Daniels, Jane; University of Nottingham, Nottingham Clinical Trials Unit<br>Naidu, Babu; University of Birmingham, Institute of Inflammation and Ageing; Heart of England NHS Foundation Trust, Department of Thoracic Surgery<br>Melody, Teresa; Heart of England NHS Foundation Trust, Academic Department of Anaesthesia, Critical Care, Pain and Resuscitation<br>Goebel, Andreas; University of Liverpool, Institute of Translational Medicine<br>Wilson, Matthew; University of Sheffield, Health Services Research<br>Kumar, Sajith; Heart of England NHS Foundation Trust, Department of Anaesthesia<br>Szentgyorgyi, Lajos; University Hospital of South Manchester NHS Foundation Trust, Department of Anaesthesia<br>Flanagan, Sarah; University of Birmingham, Institute of Applied Health Research<br>Shah, Rajesh; University Hospital of South Manchester NHS Foundation Trust, Department of Thoracic Surgery<br>Worrall, Antony; Heart of England NHS Foundation Trust<br>Gao, Fang; University of Birmingham, Institute of Inflammation and Ageing, College of Medical and Dental Sciences; Heart of England NHS Foundation Trust, Academic Department of Anaesthesia, Critical Care, Pain and Resuscitation

Keywords:	Pain management < ANAESTHETICS, Adult anaesthesia < ANAESTHETICS, Thoracic surgery < SURGERY, Clinical trials < THERAPEUTICS

For peer review only

A RANDOMISED CONTROLLED TRIAL TO INVESTIGATE THE EFFECTIVENESS OF THORACIC EPIDURAL AND PARAVERTEBRAL BLOCKADE IN REDUCING CHRONIC POST-THORACOTOMY PAIN (TOPIC) – A PILOT STUDY TO ASSESS FEASIBILITY OF A LARGE MULTI-CENTRE TRIAL

Authors:

J Yeung[1,2,], L Middleton[3], K Tryposkiadis[3], A Kerr[1], J Daniels[4], B Naidu[1,5], T Melody[1], A Goebel[6], M Wilson[7], S Kumar[1], L Szentgyorgyi[8], S Flanagan[9], R Shah[8], A Worrall[1], and F Gao[1,5], On behalf of TOPIC Investigator Group

[1] Department of Anaesthesia, Heart of England NHS Foundation Trust, Birmingham, United Kingdom

[2] Warwick Medical School, University of Warwick, Coventry, United Kingdom

[3] Birmingham Clinical Trials Unit, University of Birmingham, Birmingham, United Kingdom

[4]Nottingham Clinical Trials Unit, University of Nottingham, Nottingham, United Kingdom

[5]Institute of Inflammation and Ageing, University of Birmingham, Birmingham, United Kingdom

[6]University of Liverpool and Walton Centre NHS Foundation Trust, Liverpool, United Kingdom

[7]School of Health and Related Research, University of Sheffield, Sheffield, United Kingdom

[8]Manchester University NHS Foundation Trust, Manchester, United Kingdom

[9]Institute of Applied Health Research, University of Birmingham, Birmingham, United Kingdom

Corresponding author: Fang Gao

E-mail: f.gaosmith@bham.ac.uk Tel: 01214147483

Institute of Inflammation and Ageing, College of Medical and Dental Sciences, University of Birmingham, Birmingham B15 2TT, United Kingdom

Word Count 3618

For peer review only

ABSTRACT

Objectives: Thoracotomy is considered one of the most painful surgical procedures. The incidence of chronic post-thoracotomy pain (CPTP) is up to 50%. Paravertebral blockade (PVB) may be superior to thoracic epidural blockade (TEB) in preventing CPTP.

Design: A randomised, parallel, external pilot study was conducted to assess whether a large randomised trial of TEB and PVB with CPTP as the primary outcome is feasible.

Setting: Two adult thoracic centres in the UK

Participants: All adult patients admitted for elective open thoracotomy. Participants were excluded if they were ASA physical status IV or V; or if there is contraindication to local anaesthetics; infection near the proposed puncture site; coagulation/thoracic spine disorders; required chest wall resection or emergency thoracic surgery or had a previous thoracotomy.

Results: All patients presenting for thoracotomy were screened over a 12-month period with 194 found to be eligible. Of these, 69 (36%) were randomised (95%CI: 29%-42%). Discounting five participants who died, 54 of 64 participants (84%) returned questionnaire booklets at six months. The number of participants indicating at least a moderate level of chest pain at six months was lower with PVB but with high levels of uncertainty (RR: 0.7; 95%CI: 0.3-1.7 for worst pain; RR: 0.3; 95%CI: 0.0-2.8 for average). There were no safety concerns.

Conclusions: A large multicentre RCT of PVB versus TEB is feasible as it is possible to randomise and follow up participants with high fidelity. Pain scores were lower on average with PVB compared to TEB but a much larger trial is required to confirm this reliably.

Trial Registration: ISRCTN45041624

For peer review only

Strengths and limitations of this study

- Chronic post thoracotomy pain can be debilitating to patients and research into preventative perioperative strategies is currently lacking.
- Randomised controlled trial comparing the effect of thoracic epidural and paravertebral block on chronic pain in patients undergoing open thoracotomy.
- Feasibility study designed to test study protocol, assess patient recruitment and refine study processes in order to conduct definitive study.
- Definitive study is required to answer which analgesic technique can reduce chronic pain.

For peer review only

BACKGROUND

An estimated 7200 thoracotomies (surgical incision into the chest wall) are performed annually in the UK, most commonly to treat lung cancer.[1] Thoracotomy is considered one of the most painful surgical procedures due to tissue, muscle and nerve damage from the incision and wound retraction. Intercostal nerve injury can result in a high risk of persistent pain for months after surgery.[2-4] The incidence of chronic post-thoracotomy pain (CPTP; defined as pain that recurs or persists at least two months following the surgery)[5] is thought to be as high as 50%.[6]

Two main analgesic techniques are commonly used for perioperative pain control during thoracotomy. Thoracic epidural blockade (TEB) blocks nerves that supply the chest with local anaesthetic bilaterally, at spinal cord level. It acts by reducing onward transmission of painful nerve signals but may not abolish them completely.[7,8] Paravertebral blockade (PVB), involves injecting local anaesthetic into the paravertebral space on the side of surgery and may completely block painful nerve signals from reaching the spinal cord.[9,10] This total blockade of nerve signals could remove the stimulus for 'central sensitisation' which underpins the formation of chronic pain pathways. PVB could be uniquely effective in preventing long-term pain[11], and there is evidence from a recent trial of two techniques in breast surgery to support this premise.[12] With limited current evidence to support the most effective choice of anaesthetic technique in preventing CPTP, current UK practice varies greatly.[13] A recent Cochrane Review recommended that a high quality randomised controlled trial (RCT) to compare TEB and PVB with the primary outcome of chronic pain is urgently needed.[14]

The overall aim of this research is to determine whether PVB at thoracotomy is more effective in reducing CPTP compared to TEB. To answer this question a large, multi-centre RCT is required. The specific aim of this pilot study was to assess the feasibility of conducting a larger trial and to enhance the likelihood of its success by developing the necessary structure and processes that a large trial would need. Objectives included: an assessment of the effectiveness of the patient identification and screening process; identification of reasons for failure to randomise; development of educational and training materials for surgeons and anaesthetists; evaluation of robustness of in-hospital data collection processes; assessment of trial processes including impact on participants and staff.

METHODS

We conducted a randomised, parallel group, external pilot trial of TEB versus PVB for perioperative pain control during thoracotomy. Recruitment took place over 12 months in two adult thoracic centres: Heartlands Hospital, Birmingham and University Hospital South Manchester

For peer review only

(Wythenshawe). This study was approved by NHS Research Ethics Committee (NRES Committee East Midlands - Derby, REC number 14/EM/1280) and was prospectively registered (ISRCTN45041624).

Population and inclusion/exclusion criteria

Eligible participants were all adults admitted for an elective open thoracotomy. Participants were excluded if they had any of the following: ASA (classification of fitness) physical status IV or V (as these patients are unlikely to present for elective surgery due to their physical condition); contraindication to local anaesthetics; infection near the proposed puncture site; coagulation/thoracic spine disorders; required chest wall resection or emergency thoracic surgery or had a previous thoracotomy (scarring due to previous surgery can limit the effectiveness of paravertebral block and these patients may already have existing chronic pain).

Study conduct

All patients thought to fulfil the eligibility criteria were approached with study information at their pre-operative assessment clinic. They were provided with a Patient Information Sheet and given the opportunity to consider participation. Once eligibility was confirmed and written informed consent was obtained, randomisation was performed prior to surgery using a web-based central randomisation system (via Birmingham Clinical Trials Unit) to allocate patients to either TEB or PVB in a 1:1 ratio. Minimisation was used to achieve balance between sex, age (<65 years or ≥65 years), thoracotomy for lung cancer resection or for other indication and study site. An Oversight Committee was formed to provide independent guidance to the Trial Management Committee and to review accruing safety information during the period of recruitment. Public and patient involvement was integral to the study throughout.

Interventions

Interventions were delivered by experienced thoracic anaesthetists, trained and deemed competent in both anaesthetic techniques. Participants were not told specifically about their allocated intervention. It was not possible to blind anaesthetists, surgeons or nursing staff due to the nature of the interventions. PVB was performed using three single injections pre-incision with the patient awake or asleep, at the levels of T3-4, 5-6 and 7-8 with 15ml 0.25% levobupivicaine/bupivacaine, using a landmark technique at each level. The PVB catheter was then placed at T5 under direct vision by a surgeon during surgery. A loading dose of 10ml 0.25% levobupivicaine/bupivacaine was administered before chest closure, followed by infusion of 0.125% levobupivicaine/bupivacaine 0.1-0.25ml kg^{-1} hr^{-1} until operation end. TEB was inserted pre-incision with the patient awake or asleep, with a catheter inserted at the spinal level supplying the skin at the incision site (normally T5-6), a test dose of 3ml of 0.5% bupivacaine, and a loading dose of 0.25% levobupivicaine/bupivacaine

For peer review only - http://bmjopen.bmj.com/site/about/guidelines.xhtml

0.1ml kg^{-1} with up to 3mg of diamorphine. This was followed by infusion of 0.125% levobupivicaine/bupivacaine with 2µg ml^{-1} fentanyl at 0.1-0.25 ml kg^{-1} hr^{-1}. Further information on delivery and training on the interventions is available in the study protocol.[15]

Pilot outcomes

The following outcomes and targets were set a-priori as being indicative that a larger trial would be feasible to conduct.[15] These were: i) patient recruitment rate (the proportion of eligible patients randomised) at least 25%; ii) screening rate (the proportion of all patients listed for elective thoracotomy that were screened for eligibility and recorded on a screening log) at least 90%; iii) clinicians' willingness to recruit: national survey of consultant thoracic anaesthetist indicating the number willing to participate in a larger study at least 70%; iv) data completion – number of anaesthetic/peri-operative forms completed at least 90% and follow-up questionnaires completed at six months at least 80%. The study was given a 12-month recruitment period, after which screening would stop.

Clinical and participant reported outcome measures

Baseline data (including participant completed questions as indicated below) were recorded at the pre-operative assessment appointment. Intraoperative (anaesthetic technique and use), post-operative and discharge data (analgesic use, pain scores on days 1-3, acute complications, mortality and length of stay) were collected by the study team. Three and six-month post-randomisation follow-up was conducted by postal questionnaire. This included: Visual Analogue Scales (VAS)[16] for worst and average chest pain (overall, at rest, after coughing, after moving, after physiotherapy; with higher score - maximum 10 - indicating higher levels of pain), Brief Pain Inventory (BPI)[17], Neuropathic Pain Scale (NPS)[18], generic health-related quality of life (EQ-5D-5L)[19], Hospital Anxiety and Depression Scale (HADS)[20] and assessment of satisfaction and participant blinding. Serious adverse events were collected throughout.

Statistics

We expected to recruit between 50-75 participants over a 12-month recruitment period, depending on the number found to be eligible. We estimated that there would be approximately 500 open elective thoracotomies from the two sites, of which 60% would potentially be eligible (300). Using our own target criteria of 25% recruited would yield 75 participants. This number would be sufficient to measure the recruitment rate to uncertainty width up to approximately 10%.

Feasibility outcomes were considered with simple summary statistics (proportions and percentages), with uncertainty estimates provided by 95% confidence intervals. Clinical and participant reported

For peer review only

outcome measures were analysed with point estimates (RR or mean difference) and 95% confidence intervals, adjusting for the minimisation variables. They were not subject to hypothesis testing as the size of the sample would not allow reliable assessment of efficacy.[21] Participants were considered in the groups they were randomised to regardless of compliance (intention-to-treat).

Qualitative assessment and survey of practice

In addition to the clinical assessment, semi-structured qualitative interviews were undertaken with 18 patients at 6-8 weeks discharge to gauge the impact of the trial on their hospital care and eight clinical staff (four anaesthetists, one surgeon and three members of the research team) to aid insight into trial related processes. An electronic national survey of anaesthetists on willingness to participate in a future trial was also conducted via the Association for Cardiothoracic Anaesthesia and Critical Care membership in 26 UK thoracic units.

Patient involvement

Our study question in chronic pain post thoracotomy is in the top ranked research priorities voted by patient 'What can we do to stop patients developing chronic pain after surgery?' The development of study protocol was designed in conjunction with our patient partners who also participated as a member of trial management group and trial steering committee. Our patient partners reviewed study design, paying attention to acceptability of randomisation analgesic techniques in perioperative setting and burden of study participation. Study results including plan for future study has been disseminated within local and national patient research ambassador groups.

RESULTS

Participants and follow-up

564 patients were screened for eligibility from July 2015 to July 2016 - 100% of those listed for elective thoracotomy - with 194 patients found to be eligible (Figure 1). The most common reasons for ineligibility were: undergoing video-assisted thoracoscopic surgery (174/370, 47%); participation in another study which did not allow co-enrolment (59/370, 16%); previous thoracotomy (31/370, 8%). Of the 194 patients, 69 (36%, 95%CI: 29% to 42%) were ultimately randomised. Of the others, the most common reasons for non-randomisation were: anaesthetists trained to perform PVB not available (24/125, 19%); did not want to be randomised (15/125, 12%); patient had preference for PVB (15/125, 12%). Details of randomised participants are given in Table 1. The minimisation algorithm provided appropriate balance for the balancing factors; the average age of participants was 66 years with the vast majority were having a lung cancer resection operation (60/69, 87%). Seven participants (10%) reported previous problems with (unspecified) chronic pain.

For peer review only

At six months post-randomisation, 54 of 63 (86%) questionnaire booklets were returned, discounting 5 participants who died during the follow-up period and one participant who withdrew from the study. Of these, only one did not complete their VAS questions.

For peer review only

Table 1. Baseline characteristics of patients

		PVB (n=35)	TEB (n=34)
Age >=65, years[1]		21 (60)	20 (59)
Age, years	Mean (SD)	66.0 (10.6)	65.9 (7.9)
Gender=male[1]		19 (54)	20 (59)
Centre[1]	Birmingham Heartlands	23 (66)	26 (76)
	University Hospital South Manchester	12 (34)	8 (24)
Reason for thoracotomy=lung cancer resection[1]		30 (86)	30 (88)
ASA[2] physical status	1: Normal healthy	0 (-)	2 (6)
	2: Mild systemic disease	13 (37)	10 (30)
	3: Severe systemic disease	22 (63)	21 (64)
	Missing	-	1
ECOG[3] performance status	0: Normal activity	20 (57)	19 (58)
	1: Symptomatic but nearly fully ambulatory	14 (40)	14 (42)
	2: Symptomatic but bed <50% daytime	1 (3)	0 (-)
	3: Symptomatic bed>50% daytime	0 (-)	0 (-)
	4: Unable to get out of bed	0 (-)	0 (-)
	Missing	-	1
Dyspnoea	0: None	17 (48)	17 (52)
	1: Slight	16 (46)	13 (39)
	2: Moderate	2 (6)	1 (3)
	3: Moderately severe	0 (-)	2 (6)
	4: Severe	0 (-)	0 (-)
	5: Very severe	0 (-)	0 (-)
	Missing	-	1
Smoking status	Never smoked	7 (20)	6 (18)
	Stopped>1 year	19 (54)	18 (55)
	Stopped>6 weeks	3 (9)	3 (9)
	Stopped<6 weeks	1 (3)	4 (12)
	Current smoker	5 (14)	2 (6)
	Missing	-	1
Smoking-pack years	Median [IQR]	37 [20, 49]	37 [20, 50]
	Missing	-	1
Alcohol units per week	Median [IQR]	3 [0, 13.5]	0 [0, 9.7]
	Missing	-	1
Previous medical history=yes	COPD	7 (20)	6 (18)
	Ischaemic heart disease	4 (11)	3 (9)
	Congestive cardiac failure	0 (-)	1 (3)
	Hypertension	18 (51)	15 (45)
	Diabetes diet controlled	0 (-)	3 (9)
	Diabetes oral medication	6 (17)	3 (9)
	Diabetes insulin controlled	2 (6)	1 (3)
	Renal failure	1 (3)	1 (3)
	Previous stroke	0 (-)	0 (-)
	Hyperthyroidism	0 (-)	0 (-)
	Hypothyroidism	5 (14)	2 (6)
	Other cancer	12 (34)	7 (21)
	Chronic pain	2 (6)	5 (15)
	Missing	-	1
BMI, kg/m^2	Mean (SD)	28.4 (5.8)	27.7 (5.9)
	Missing	-	1

Frequency (%) presented (unless otherwise stated)

[1] minimisation variable; [2] American Society of Anesthesiologists; [3] Eastern Cooperative Oncology Group

For peer review only

Analgesic technique

Of the 69 randomised, 67 (97%) received their randomised intervention. One participant allocated PVB received TEB as the pleura was removed and the surgeon was unable to place the PVB. Another participant was not considered fit for surgery and was withdrawn from the study. Discounting this participant, all anaesthetic technique and intra-operative forms were completed. A summary of the anaesthetic techniques is given in Table 2; a summary of the operative details and other analgesics provided to participants up to discharge is provided in the Appendix (Tables S1, S2).

Table 2. Anaesthetic technique summary

		**PVB (n=35)**	**TEB (n=33)**[1]
Difficulty in insertion		4 (12)[2]	14 (42)
Failure to deliver technique		1 (3)	0 (-)
Intraoperative use			
Preoperative bolus given		26 (74)	25 (76)
Bolus drug given through catheter		14 (40)	24 (73)
Infusion given through catheter		23 (66)	21 (64)
Post-operative settings			
Local anaesthetic was Bupivacaine (as opposed to Levobupivacaine)		32 (94)[2]	19 (58)
Concentration	0.1%	20 (59)[2]	11 (33)
	0.125%	4 (12)[2]	22 (67)
	0.25%	10 (29)[2]	0 (-)
Fentanyl	None	32 (91)	3 (9)
	2 mcg/ml	0 (-)	0 (-)
	4 mcg/ml	3 (9)	30 (91)
Starting rate, ml/hr	Median [IQR]	20 [10, 20][2]	6 [6, 8]
Prescribed range, ml/hr	Median [IQR]	20 [15, 20][2]	12 [8, 15]

Frequency (%) presented (unless otherwise stated)
[1] one participant received neither technique as they did not have an operation; [2] one response missing (n=34)

For peer review only

Patient reported outcomes

Levels of pain in the three days post-operation appeared similar in both groups (Appendix Table S3). The number of patients indicating at least a moderate level of average or worst chest pain (based on commonly used threshold of >3 or ≥4[22, 23]) was lower with PVB compared to TEB at six months, but with expected high levels of uncertainty given the limited size of sample (Table 3; full results given in Appendix Table S4). Similar patterns were seen from other questionnaire responses, with scores generally favouring PVB on average, although most estimates of uncertainty did not discount parity (Table 4).

Table 3. Incidence of significant (>3 or >=4) or severe pain (>=7) from Visual Analogue Scale Scores

	PVB n (%)	TEB n (%)	RR (95% CI) [1]
Average chest pain overall>3			
3 months	5/25 (20.0)	6/21 (28.6)	0.7 (0.2, 2.0)
**6 months**	2/27 (7.4)	5/25 (20.0)	0.4 (0.1, 1.7)
Average chest pain overall>=4			
3 months	5/25 (20.0)	4/21 (19.0)	1.1 (0.3, 3.4)
**6 months**	1/27 (3.7)	3/25 (12.0)	0.3 (0.03, 2.8)
Average chest pain overall>=7			
3 months	0/25 (-)	2/21 (10.0)	- [2]
**6 months**	0/27 (-)	1/25 (4.0)	- [2]
Worst chest pain overall>3			
3 months	9/24 (37.5)	7/23 (30.4)	1.2 (0.6, 2.8)
**6 months**	7/27 (25.9)	11/26 (42.3)	0.6 (0.3, 1.3)
Worst chest pain overall>=4			
3 months	8/24 (33.3)	7/23 (30.4)	1.1 (0.5, 2.5)
**6 months**	7/27 (25.9)	9/26 (34.6)	0.75 (0.3, 1.7)
Worst chest pain overall>=7			
3 months	6/24 (25.0)	5/23 (21.7)	1.2 (0.4, 3.3)
**6 months**	2/27 (7.4)	5/26 (19.2)	0.4 (0.1, 1.8)

[1] RR<0 indicate less incidence with PVB; [2] unable to estimate

For peer review only

Table 4. Results of patient reported outcomes

	PVB mean score (SD, n)	TEB mean score (SD, n)	Difference between groups (95% CI)
**Brief pain inventory (BPI) results summary[1]**			
Pain severity score (0-10, higher=worse pain)			
Baseline	0.9 (1.7, 34)	1.3 (2.0, 32)	-0.4 (-1.3, 0.5)
24 hours post-surgery	5.3 (1.7, 33)	3.9 (2.7, 32)	1.4 (0.3, 2.5)
48 hours post-surgery	5.2 (1.7, 30)	5.1 (2.5, 31)	0.1 (-0.9, 1.2)
72 hours post-surgery	4.9 (1.7, 23)	5.6 (2.0, 25)	-0.7 (-1.8, 0.4)
Discharge	4.0 (1.4, 31)	4.1 (2.4, 32)	-0.1 (-1.1, 0.9)
3 months	1.6 (1.7, 22)	2.5 (2.5, 21)	0.9 (-2.2, 0.4)
**6 months**	1.3 (1.8, 25)	2.3 (2.6, 23)	-1.0 (-2.3, 0.3)
Pain interference score (0-10, higher=worse pain)			
Baseline	0.6 (1.3, 34)	1.5 (2.2, 32)	-0.9 (-1.8, -0.1)
24 hours post-surgery	5.8 (2.1, 30)	5.4 (2.9, 28)	0.4 (-0.8, 1.8)
48 hours post-surgery	6.0 (2.0, 30)	5.8 (2.2, 27)	0.2 (-0.9, 1.3)
72 hours post-surgery	5.4 (2.3, 21)	5.8 (2.6, 23)	-0.4 (-1.9, 1.1)
Discharge	4.7 (2.0, 23)	4.3 (2.5, 29)	0.4 (-0.9, 1.7)
3 months	1.1 (1.4, 24)	2.8 (3.0, 22)	-1.7 (-3.1, -0.3)
**6 months**	1.6 (2.2, 27)	1.9 (2.5, 25)	-0.3 (-1.6, 1.0)
**Neuropathic pain scale (NPS) results summary[1]**			
How intense does your pain feel? (0-10, higher=worse pain)			
Baseline	1.7 (2.8, 33)	1.8 (2.8, 33)	-0.1 (-1.5, 1.3)
Discharge	5.8 (2.8, 31)	5.4 (3.8, 31)	0.4 (-1.3, 2.1)
3 months	2.2 (2.4, 25)	3.6 (3.8, 21)	-1.4 (-3.4, 0.5)
**6 months**	2.2 (2.7, 27)	2.7 (3.2, 26)	-0.5 (-2.2, 1.1)
How sharp does your pain feel? (0-10, higher=worse pain)			
Baseline	1.1 (2.8, 33)	1.6 (3.4, 32)	-0.5 (-2.1, 1.1)
Discharge	5.5 (3.2, 31)	5.4 (4.0, 31)	0.1 (-1.7, 1.9)
3 months	2.6 (3.0, 25)	3.2 (4.0, 21)	-0.6 (-2.8, 1.5)
**6 months**	3.0 (3.3, 27)	2.4 (3.3, 26)	0.6 (-1.3, 2.4)
How hot does your pain feel? (0-10, higher=worse pain)			
Baseline	1.0 (2.6, 33)	1.3 (2.7, 32)	-0.3 (-1.6, 1.0)
Discharge	3.6 (3.4, 31)	2.4 (2.9, 31)	1.2 (-0.4, 2.8)
3 months	1.6 (2.8, 25)	2.5 (3.3, 21)	-0.9 (-2.7, 1.0)
**6 months**	1.2 (2.0, 28)	1.6 (2.9, 26)	-0.4 (-1.7, 1.0)
How dull does your pain feel? (0-10, higher=worse pain)			
Baseline	1.8 (2.9, 33)	1.5 (2.5, 33)	0.3 (-1.0, 1.7)
Discharge	2.9 (2.7, 30)	3.9 (2.8, 30)	-1.0 (-2.4, 0.5)
3 months	2.6 (2.1, 25)	3.3 (3.3, 21)	-0.7 (-2.4, 0.9)
**6 months**	2.1 (2.8, 28)	2.3 (2.8, 26)	-0.2 (-1.8, 1.2)
How cold does your pain feel? (0-10, higher=worse pain)			
Baseline	0.3 (1.6, 33)	0.4 (1.7, 32)	-0.1 (-0.9, 0.7)
Discharge	1.5 (2.5, 30)	1.0 (2.2, 31)	0.5 (-0.7, 1.7)
3 months	0.2 (0.5, 25)	1.5 (2.9, 21)	-1.3 (-2.7, -0.0)
**6 months**	0.3 (0.9, 28)	0.7 (1.6, 26)	-0.4 (-1.1, 0.3)
How sensitive is your pain? (0-10, higher=worse pain)			
Baseline	0.5 (1.9, 33)	1.1 (2.5, 32)	-0.6 (-1.7, 0.5)
Discharge	2.3 (2.9, 30)	2.8 (2.9, 32)	-0.5 (-1.9, 1.0)
3 months	2.2 (3.0, 25)	2.1 (2.7, 23)	0.1 (-1.6, 1.7)
**6 months**	1.6 (2.0, 28)	2.5 (3.1, 26)	-0.9 (-2.4, 0.5)
How itchy is your pain? (0-10, higher=worse pain)			
Baseline	0 (0, 33)	0.8 (2.0, 32)	-0.8 (-1.5, -0.0)
Discharge	2.2 (2.8, 30)	2.6 (3.3, 31)	-0.4 (-2.0, 1.2)
3 months	1.2 (2.5, 25)	1.8 (2.6, 22)	-0.6 (-2.1, 0.9)
**6 months**	0.4 (0.7, 28)	2.1 (2.6, 25)	-1.7 (-2.8, -0.6)
How unpleasant is your pain? (0-10, higher=worse pain)			
Baseline	1.9 (2.9, 31)	2.1 (3.3, 33)	-0.2 (-1.7, 1.4)
Discharge	6.2 (3.0, 30)	5.6 (3.3, 30)	0.6 (-1.1, 2.2)
3 months	2.8 (2.6, 24)	4.0 (3.9, 21)	-1.2 (-3.3, 0.8)

For peer review only

**6 months**	2.4 (2.7, 28)	3.0 (3.2, 24)	0.6 (-2.3, 1.0)
How intense is your deep pain? (0-10, higher=worse pain)			
Baseline	1.3 (2.4, 32)	2.4 (3.7, 33)	-1.1 (-2.6, 0.5)
Discharge	6.2 (2.8, 31)	5.5 (3.4, 31)	0.7 (-0.9, 2.3)
3 months	3.0 (2.8, 23)	4.1 (3.7, 21)	-1.1 (-3.1, 0.9)
**6 months**	2.8 (3.0, 28)	2.5 (2.9, 25)	0.3 (-1.4, 1.9)
How intense is your surface pain? (0-10, higher=worse pain)			
Baseline	0.8 (2.1, 31)	0.7 (1.5, 32)	0.1 (-0.9, 1.0)
Discharge	3.9 (3.4, 30)	3.8 (3.3, 32)	0.1 (-1.7, 1.7)
3 months	1.9 (2.2, 24)	3.5 (3.6, 21)	-1.6 (-3.3, 0.2)
**6 months**	1.2 (1.5, 27)	2.7 (3.2, 24)	-1.5 (-2.9, -0.1)
**EQ-5D-DL results summary[2]**			
EQ-5D index score (-0.59=worst outcome, 1.0=best outcome)			
Baseline	0.75 (0.22, 35)	0.77 (0.20, 33)	-0.02 (-0.12, 0.08)
Discharge	0.49 (0.23, 27)	0.49 (0.23, 30)	-0.00 (-0.12, 0.12)
3 months	0.73 (0.17, 25)	0.65 (0.23, 23)	0.08 (-0.04, 0.20)
**6 months**	0.72 (0.23, 25)	0.69 (0.22, 25)	0.03 (-0.09, 0.16)
EQ-5D thermometer (0-100, higher=better)			
Baseline	76.6 (16.3, 34)	74.3 (17.8, 33)	2.3 (-5.9, 10.7)
Discharge	60.4 (22.7, 26)	60.2 (20.7, 30)	0.2 (-11.5, 11.9)
3 months	73.2 (21.2, 25)	67.5 (18.3, 23)	5.7 (-5.8, 17.2)
**6 months**	78.0 (14.0, 25)	64.4 (20.4, 25)	13.6 (3.6, 23.6)
**Hospital anxiety and depression index score (HADS) results summary[3]**			
Depression index score (0-21, lower=better)			
Baseline	2.9 (2.9, 33)	3.0 (2.6, 32)	-0.1 (-1.4, 1.3)
Discharge	4.3 (3.4, 28)	6.0 (5.0, 28)	-1.7 (-4.0, 0.5)
3 months	3.6 (2.7, 25)	5.5 (4.0, 21)	-1.9 (-4.1, 0.1)
**6 months**	4.5 (3.5, 24)	6.0 (4.6, 25)	-1.5 (-3.9, 0.8)
Anxiety index score (0-21, 0-lower=better)			
Baseline	6.3 (5.0, 34)	6.5 (4.4, 30)	-0.2 (-2.6, 2.1)
Discharge	5.0 (4.4, 29)	7.0 (5.3, 28)	-2.0 (-4.6, 0.6)
3 months	5.2 (4.3, 25)	6.1 (4.8, 19)	-0.9 (-3.7, 1.9)
**6 months**	5.7 (3.6, 24)	7.3 (5.0, 25)	-1.6 (-4.1, 0.9)

[1] scores<0 indicate less pain with PVB
[2] scores>0 indicate better QoL with PVB
[3] scores<0 indicate less anxiety/depression with PVB

Safety

No concerns were expressed by the independent Oversight Committee who met twice during the recruitment period to review study progress and safety data. Complications and short-term mortality data appeared similar in both groups (Appendix Table S5). There were five deaths; only one was considered to be a serious adverse event, this was aspiration pneumonitis and was not considered related to intervention (PVB). One other participant experienced a serious adverse event in the TEB group; this participant was diagnosed with acute kidney injury in the post-operative period.

Assessment of participant blinding

19/32 (60%) patients in the TEB group correctly identified the anaesthetic technique they had been allocated at discharge; the figure for PVB was 8/31 (26%); this reduced to 13/24 (52%) in the TEB group at 6 months and 5/26 (19%) in the PVB group (Appendix Table S6).

National survey of anaesthetists

Forty-three responses were returned. Of these, 27 anaesthetists indicated that they would be willing to randomise in a future study (63%). Five (12%) indicating that they would 'possibly' be willing to randomise with reasons of concern given as lack of skill and ward structure. The remaining 11 (26%) were unwilling.

Qualitative interviews

Interviewed participants had a positive experience, reporting that they felt well informed by trial staff. The consent process was felt to be well undertaken and patients felt reassured by the explanations of randomisation. A substantial number of participants reported that the study questionnaires were long and repetitive. Interviewed staff felt recruitment was successful although there were some challenges when the role was undertaken by non-clinical staff. There was a feeling that some of the data collection tools were repetitive. Clinical staff were supportive of the trial; they found that the trial processes, randomisation and procedures were very straightforward. The guidance and teaching provided for anaesthetists to get them up to speed for performing PVB was very positively received and it was considered a relatively simple procedure to learn.

DISCUSSION

Our four key indicators for feasibility were met and we have shown that a large multicentre randomised controlled trial of PVB versus TEB for peri-operative pain control with an objective of

For peer review only

assessing long-term post-thoracotomy chronic pain is feasible. It is possible to randomise and follow up patients with high fidelity over six months. In the qualitative assessment we carried out, participants and staff were largely happy with study processes. In particular, the consent, randomisation and assessment methods were considered appropriate. As hypothesised, VAS pain scores were lower with PVB compared with TEB at six months on average but with high levels of uncertainty. This would need to be investigated further in a much more substantial trial.

Whilst the evidence on short-term outcomes, e.g. minor complications and analgesic efficacy, point to PVB being at least as effective as TEB[14], current evidence on whether PVB is more effective than TEB in reducing rates of chronic pain is limited. A recent systematic review failed to attain sufficient data to compare the impact of TEB and PVB techniques on CPTP. [14] The incidence of CPTP was very poorly reported and studies did not describe how data on chronic pain was measured or collected. The lack of evidence highlights an area in need of further research. We are not aware of any current or planned randomised trials with CPTP as an outcome that may change these findings since this systematic review was published. Incidence of CPTP was slightly lower in our study - between 30-34% - than previously quoted at 49%[6], although we need to acknowledge that our figure is associated with a reasonably large amount of uncertainty given the small size of sample. A likely contributing factor to this discrepancy is the considerable and widely acknowledged variation in how pain is measured[24], as it is dependent on the measurement instrument and threshold chosen amongst other factors.[22]

This feasibility work has allowed us to fine tune study processes ahead of a larger trial. One likely modification to the future study protocol is to revise the length of the follow-up questionnaires, it was considered by participants that their length was too long and repetitive in content. Shortened and simplified follow-up questionnaire would also potentially helping to improve follow-up figures further, as lengthy questionnaires are a known predictor of participant drop-out from trials.[25]

Inclusion and exclusion criteria appeared appropriate and do not appear to need to be changed. The vast majority of ineligible participants were those scheduled to receive video-assisted thoracoscopic surgery (VATS). Whilst this procedure is becoming more popular[1], the comparison in question is not an appropriate one as TEB is not routinely used to provide pain relief for in this type of surgery. The VATS approach also does not require any rib spreading, resulting in a less invasive procedure, and whilst chronic pain is still an issue after VATS it is not considered to be as great as following open thoracotomy.[26] Unfortunately, whilst VATS is becoming more common, there will still be a substantial proportion of patients that will always be ineligible for this operation because of, for example, inability to achieve complete resection in more advanced pulmonary lung cancers. Lung cancer remains the most common cancer worldwide.[27,28]

For peer review only - http://bmjopen.bmj.com/site/about/guidelines.xhtml

It was apparent from the assessment of the ability to blind the participant to the analgesic technique that complete blinding is unlikely to be possible. Substantially more participants appeared to be able to determine they had had an epidural blockade than the paravertebral technique. Attempts to completely blind participants in any future study are likely to make processes prohibitively complex and expensive due to innate differences in the mode of delivery and action in the two analgesic techniques. This could include sham insertion techniques to disguise the randomised intervention. The question is whether patient blinding is likely to be an important potential cause of detection bias, unduly affecting the results of any future trial. The proposed primary outcome of pain rating is subjective in nature and this is a theoretical possibility. However, we believe that there is no reason to suspect that recipients of the randomised interventions have strong pre-conceptions with regard to the relative effectiveness of each analgesic technique. Furthermore, the primary outcome would be collected via questionnaires administered by post or phone, at a time remote from the original operative procedure, which are likely to be resilient to the effects of imperfect concealment.

Findings from the survey of practice were encouraging, although whilst we set a target of 70% of thoracic anaesthetists willing to randomise to a future trial as being indicative of success, we found 63% willing and 12% ‘possibly willing’. To achieve 70%, we would require solutions for those anaesthetists expressing uncertainty. The major reasons given were lack of skill and ward structure. These hurdles do not appear insurmountable if funding for further training and extra nurse support could be provided; something a large funding body may be willing to provide. A successful trial would also require full engagement with anaesthetic and thoracic surgical trainees’ network to maximise recruitment.

We have reported the study to randomised pilot study standards as recommended by CONSORT.[29] Also as recommended[30], we set objectives a priori to determine whether the pilot study would demonstrate feasibility of a larger trial. Since this study was not a definitive trial, we have been careful not to over-interpret the findings. Hence at this stage, we cannot expect that the outcomes of this pilot study to be directly translated into clinical care, as the study was not large enough to be able to detect small to moderate realistic-sized difference in rates of chronic pain, nor was the scope wide enough in terms of the number of centres involved to return a generalisable result.

Nevertheless, this pilot study is an important precursor to a larger, substantive, trial and provides invaluable information that will help to ensure its success. A definitive study - instigated by the authors of this manuscript - has now been funded by the NIHR Health Technology Assessment Programme and is expected to start recruiting in 2018 from 20 UK thoracic centres. The design of this study has been partially informed by the results of this pilot. The primary outcome will be incidence of chronic pain at six months attained from visual analogue scale responses similar to that

For peer review only - http://bmjopen.bmj.com/site/about/guidelines.xhtml

used here; other outcomes will follow IMMPACT recommendations.[31] A total sample size of 1026 participants will give 90% power (p=0.05) to detect a reduction in incidence of CPTP of 30% with TEB (a similar event rate to that noted in this pilot study) down to 20% with PVB. The rate of recruitment noted in this study has been factored into the number of sites and length of the recruitment period required; we estimate access to a pool of 5000 participants over a two and a half year recruitment period and need to recruit a fifth of these. The design will allow a definitive answer this important question.

AUTHOR'S CONTRIBUTION

FG, JY, JPD and MW conceived the topic. FG, JY, JPD, LJM and AG designed the study with the input from SK, LS, BN, MW, TM and AW. FG oversaw the running of the trial and all the authors contributed to the ongoing management of the trial. AK, RS, BN collected data for the trial. KT performed the statistical analysis. SF evaluated the qualitative data. The manuscript was drafted by LJM, JY and FG with contributions from the other authors. All the authors contributed to the interpretation of the output and revised and reviewed the paper; they are the guarantors. The Birmingham Clinical Trials Unit undertook the randomisation and data management and monitoring. The authors had full access to all the data from the study. The authors vouch for the accuracy and completeness of the data and analyses. All authors read and approved the final manuscript.

ACKNOWLEDGEMENTS

We thank the trial participants; members of the trial Oversight Committee: Tom Treasure (Chair), Alistair Macfie (independent anaesthetist), Mahmoud Loubani (independent surgeon), Lucy Bradshaw (independent statistician), Alistair Murray (patient and public representative); the Clinical Research Ambassador Group at Heart of England NHS Foundation Trust; Birmingham Clinical Trials Unit staff: Matt Hill (database programmer) and Hannah Watson (data manager).

COMPETING INTERESTS

All authors declare that they have no competing interests.

FUNDING

The study was funded by a grant from the National Institute for Health Research for Patient Benefit (RFPB grant number PB-PG-0213-30126). The funder had no role in study design, data collection, interpretation or analysis, or in writing the report for publication.

For peer review only

SPONSORSHIP

The study sponsor was the Heart of England NHS Foundation Trust. The sponsor had no role in study design, data collection, interpretation or analysis, or in writing the report for publication.

DATA SHARING STATEMENT

All available study data can be obtained from the corresponding author.

For peer review only

References

1. The Society for Cardiothoracic Surgery in Great Britain and Ireland. SCTS 2014-2015 Thoracic Registry Data. 2015.

2. Hughes R, Gao F. Pain control for thoracotomy. *Continuing Education in Anaesthesia, Critical Care and Pain.* 2005;5(2):56-60.

3. Soto RG, Fu ES. Acute pain management for patients undergoing thoracotomy. *Ann Thorac Surg.* 2003;75(4):1349-1357.

4. Kavanagh BP, Katz J, Sandler AN. Pain control after thoracic surgery. A review of current techniques. *Anesthesiology.* 1994;81(3):737-759.

5. International Association for the Study of Pain. Classification of chronic pain. Descriptions of chronic pain syndromes and definitions of pain terms. Prepared by the International Association for the Study of Pain, Subcommittee on Taxonomy. *Pain Supplement.* 1986;3:S1-226.

6. Bayman EO, Brennan TJ. Incidence and severity of chronic pain at 3 and 6 months after thoracotomy: meta-analysis. *The Journal of Pain.* 2014;15(9):887-897.

7. Dahl JB, Rosenberg J, Lund C, Kehlet H. Effect of thoracic epidural bupivacaine 0.75% on somatosensory evoked potentials after dermatomal stimulation. *Reg Anesth.* 1990;15(2):73-75.

8. Lund C, Hansen OB, Mogensen T, Kehlet H. Effect of thoracic epidural bupivacaine on somatosensory evoked potentials after dermatomal stimulation. *Anesth Analg.*1987;66(8):731-734.

9. Richardson J, Lönnqvist PA. Thoracic paravertebral block. *Br J Anaesth.* 1998;81(2):230-238.

10. Cook E, Downs C. Analgesia After Thoracotomy - The Role of the Extrapleural Paravertebral Catheter. *Australian Anaesthesia.* 2005:103-117.

11. Richardson J, Jones J, Atkinson R. The effect of thoracic paravertebral blockade on intercostal somatosensory evoked potentials. *Anesthesia & Analgesia.* 1998;87(2):373-376.

12. Karmakar MK, Samy W, Li JW, et al. Thoracic paravertebral block and its effects on chronic pain and health-related quality of life after modified radical mastectomy. *Reg Anesth Pain Med.* 2014;39(4):289-298.

13. Turner J, Yeung J. Analgesic technique in thoracic surgery: a survey of UK ACTACC members. Unpublished data.2016.

14. Yeung JH, Gates S, Naidu BV, Wilson MJ, Gao Smith F. Paravertebral block versus thoracic epidural for patients undergoing thoracotomy. *Cochrane Database Syst Rev.* 2016;2:CD009121.

15. Yeung J, Melody T, Kerr A, Naidu B, Middleton L, Tryposkiadis K, Daniels J, Gao F. Randomised controlled pilot study to investigate the effectiveness of thoracic epidural and paravertebral blockade in reducing chronic post-thoracotomy pain: TOPIC feasibility study protocol. BMJ Open. 2016 Dec 1;6(12):e012735. doi: 10.1136/bmjopen-2016-012735.

For peer review only

16. Jensen MP, Karoly P, Braver S. The measurement of clinical pain intensity: a comparison of six methods. Pain. 1986 Oct;27(1):117-26.

17. Tan G, Jensen MP, Thornby JI, et al. Validation of the Brief Pain Inventory for chronic nonmalignant pain. J Pain 2004;5:133–7.

18. Galer BS, Jensen MP. Development and preliminary validation of a pain measure specific to neuropathic pain: the Neuropathic Pain Scale. Neurology 1997;48:332–8.

19. Herdman M, Gudex C, Lloyd A, et al. Development and preliminary testing of the new five-level version of EQ-5D (EQ-5D-5L). Qual Life Res 2011;20:1727–36.

20. Zigmond AS, Snaith RP. The hospital anxiety and depression scale. Acta Psychiatr Scand. 1983;67:361–70.

21. Moore CG, Carter RE, Nietert PJ, Stewart PW. Recommendations for planning pilot studies in clinical and translational research. Clin Transl Sci. 2011 Oct;4(5):332-7. doi: 10.1111/j.1752-8062.2011.00347.x.

22. Jensen MP, Chen C, Brugger AM. Interpretation of visual analog scale ratings and change scores: a reanalysis of two clinical trials of postoperative pain. J Pain. 2003;4:407-14.

23. Palos GR, Mendoza TR, Mobley GM, Cantor SB, Cleeland CS. Asking the community about cutpoints used to describe mild, moderate, and severe pain. J Pain. 2006 Jan;7(1):49-56.

24. Litcher-Kelly L, Martino SA, Broderick JE, Stone AA. A systematic review of measures used to assess chronic musculoskeletal pain in clinical and randomized controlled clinical trials. The journal of pain: official journal of the American Pain Society. 2007;8(12):906-913.doi:10.1016/j.jpain.2007.06.009.

25. Edwards P, Roberts I, Sandercock P, Frost C. Follow-up by mail in clinical trials: does questionnaire length matter? Control Clin Trials. 2004 Feb;25(1):31-52

26. Bendixen M, Jørgensen OD, Kronborg C, Andersen C, Licht PB. Postoperative pain and quality of life after lobectomy via video-assisted thoracoscopic surgery or anterolateral thoracotomy for early stage lung cancer: a randomised controlled trial. Lancet Oncol. 2016 Jun;17(6):836-844. doi: 10.1016/S1470-2045(16)00173-X. Epub 2016 May 6.

27. Ferlay J, Soerjomataram I, Ervik M, Dikshit R, Eser S, Mathers C et al. GLOBOCAN 2012 v1.0, Cancer Incidence and Mortality Worldwide: IARC Cancer Base No. 11.

28. Stewart BW, Wild CP, editors. World cancer report 2014. Lyon: International Agency for Research on Cancer; 2014.

29. Eldridge SM, Chan CL, Campbell MJ, Bond CM, Hopewell S, Thabane L, Lancaster GA. CONSORT 2010 statement: extension to randomised pilot and feasibility trials. BMJ. 2016 Oct 24;355:i5239. doi: 10.1136/bmj.i5239.

For peer review only

30. Thabane L, Ma J, Chu R, Cheng J, Ismaila A, Rios LP, Robson R, Thabane M, Giangregorio L, Goldsmith CH. A tutorial on pilot studies: the what, why and how. BMC Medical Research Methodology201010:1 https://doi.org/10.1186/1471-2288-10-1.

31. Dworkin RH, Turk DC, Farrar JT, Haythornthwaite JA, Jensen MP, Katz NP et al. Core outcome measures for chronic pain clinical trials: IMMPACT recommendations. Pain. 2005; 113:9-19.

For peer review only

LEGEND FOR FIGURE 1

Figure 1. Flow chart of patients through the study

For peer review only

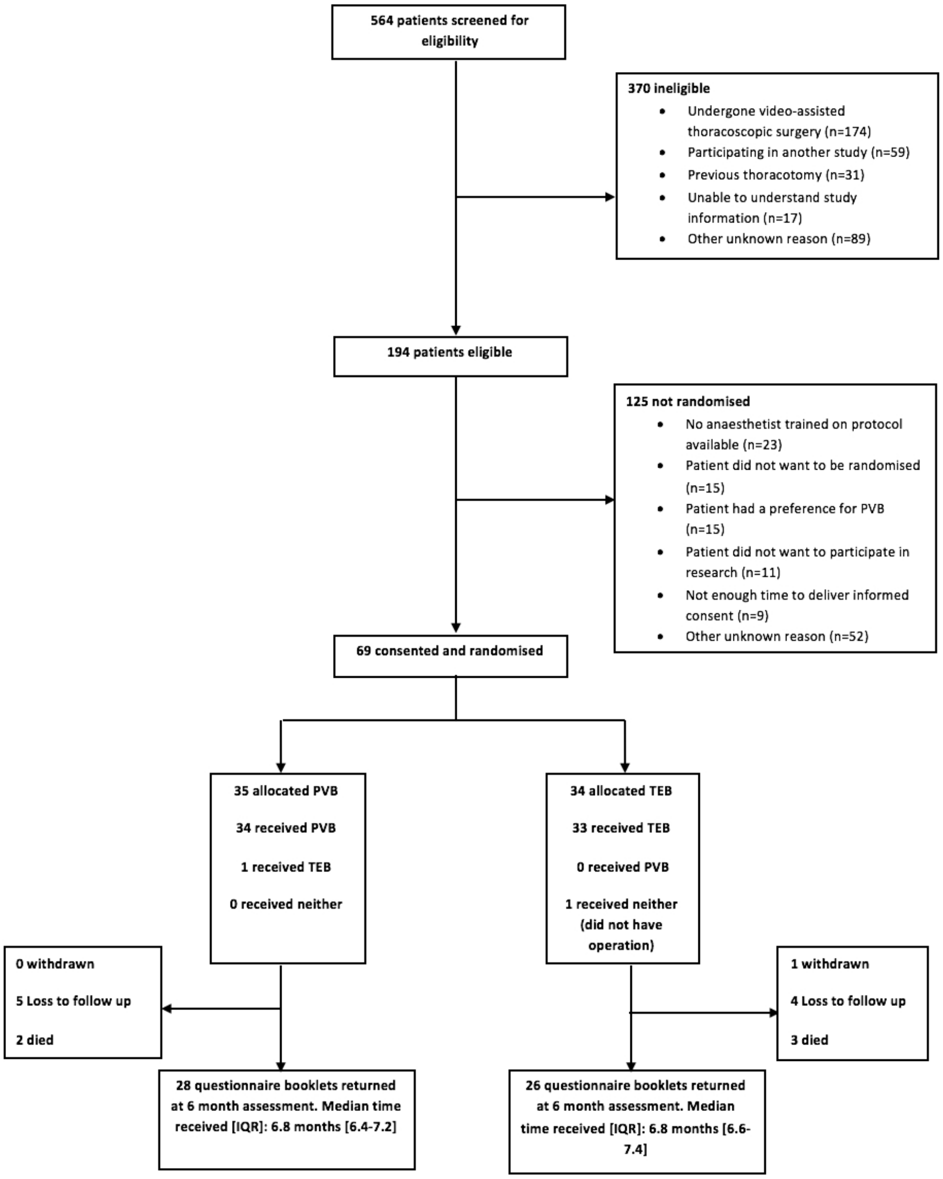

Figure 1

79x98mm (300 x 300 DPI)

TOPIC PILOT STUDY SUPPLEMENTARY APPENDIX

Table S1: Intraoperative data summary 2

Table S2: Analgesic use summary 3

Table S3: Post-operative (in hospital) pain scores summary 4

Table S4: Comprehensive summary of Visual Analogue Scale (VAS) scores 5

Table S5: Complications, short-term mortality and length of stay summary 7

Table S6: Patient satisfaction and blinding questions results summary 8

For peer review only

Table S1: Intraoperative data summary

		PVB (n=35)	TEB (n=33)[1]
Vasopressor/inotrope used		30 (86)	27 (82)
Delivery route	Boluses	24 (80)	24 (89)
	Infusion	3 (10)	1 (4)
	Both	3 (10)	2 (7)
Overall fluid therapy and balance	Median [IQR]	1000 [1000, 2000]	1000 [1000, 1500]
Grade of surgeon was consultant (as opposed to trainee)		21 (60)	18 (55)
Side of operation was left (as opposed to right)		9 (26)	10 (30)
Type of operation	Lobectomy	18 (51)	18 (55)
	Pneumonectomy	3 (9)	2 (6)
	Segmentectomy	1 (3)	1 (3)
	Wedge	3 (9)	2 (6)
	Lung biopsy	1 (3)	0 (-)
	Wedge and Segmentectomy	0 (-)	1 (3)
	Lobectomy and Wedge	1 (3)	0 (-)
	Lobectomy and Other	2 (6)	2 (6)
	Other	6 (17)	7 (21)
Surgical approach	Anterolateral	4 (12)[2]	2 (6)
	Posterolateral	22 (65)[2]	23 (70)
	Muscle sparing	0 (-)[2]	1 (3)
	Mini-thoracotomy	1 (3)[2]	0 (-)
	Posterolateral and Muscle sparing	7 (21)[2]	6 (18)
	Mini-limited lateral	0 (-)[2]	1 (3)

Frequency (%) presented (unless otherwise stated)
[1] one participant received neither technique as they did not have an operation; [2] one response missing (n=34)

For peer review only

Table S2: Analgesic use summary

			In Hospital (post-surgery)							
	Intraoperative		Day 1		Day 2		Day 3		Between day 3 and discharge/final assessment	
**Drug used**	**PVB**	**TEB**	**PVB**	**TEB**	**PVB**	**TEB**	**PVB**	**TEB**	**PVB**	**TEB**
Paracetamol	29	29	1	-	-	-	-	-	-	-
Diclofenac	3	-	3	2	-	-	-	-	-	-
Remifentanil	-	-	6	5	2	-	-	-	-	-
Fentanyl	-	-	16	11	5	4	2	-	-	-
Morphine	2	-	6	1	14	3	3	2	2	-
Diamorphine	-	2	-	10	1	7	-	1	-	-
Gabapentin	-	-	-	-	-	-	-	1	-	-
Alfentanil	-	-	1	2	3	10	1	1	-	-
Bupivicaine	-	-	-	-	-	-	-	1	-	-
Levobupivicaine	-	-	-	-	-	1	-	-	-	-
Pregabalin	-	-	-	-	-	-	1	-	-	-
Ketorolac	-	-	-	-	-	2	3	2	2	-

Number of participants administered drug indicated (total number given PVB=35 and total number given TEB=33)

For peer review only

3

Table S3: Post-operative (in hospital) pain scores summary

	PVB mean score (SD, n)	TEB mean score (SD, n)	Difference between groups (95% CI) [1]
Pain score at rest (0-3, higher=worse pain)			
Day 1	0.4 (0.7, 34)	0.5 (1.0, 33)	-0.1 (-0.5, 0.3)
Day 2	0.5 (0.7, 33)	0.7 (1.0, 32)	-0.2 (-0.6, 0.3)
Day 3	0.2 (0.4, 27)	0.6 (0.7, 27)	-0.4 (-0.8, -0.1)
Pain score at movement (0-3, higher=worse pain)			
Day 1	0.9 (0.9, 34)	0.8 (1.0, 33)	0.1 (-0.3, 0.6)
Day 2	1.2 (0.8, 33)	1.3 (0.9, 32)	-0.1 (-0.5, 0.4)
Day 3	0.8 (0.9, 27)	1.0 (1.0, 27)	-0.2 (-0.8, 0.3)

[1] scores<0 indicate less pain with PVB

For peer review only

Table S4: Comprehensive summary of Visual Analogue Scale (VAS) scores

	PVB mean score (SD, n)	TEB mean score (SD, n)	Difference between groups (95% CI) [1]
Average chest pain overall (0-10, higher=worse pain)			
Baseline	0.6 (1.8, 32)	0.7 (1.7, 33)	-1.0 (-1.0, 0.8)
24 hours post-surgery	5.2 (2.5, 33)	3.7 (3.4, 32)	1.5 (-0.02, 2.9)
48 hours post-surgery	6.4 (1.8, 31)	5.5 (2.7, 31)	0.9 (-0.3, 2.1)
72 hours post-surgery	5.1 (25, 2.4)	6.1 (2.5, 26)	-1.0 (-2.4, 0.3)
Discharge	4.9 (1.9, 29)	4.3 (2.5, 32)	0.6 (-0.5, 1.7)
3 months	2.1 (1.8, 25)	2.0 (2.6, 21)	0.1 (-1.3, 1.4)
**6 months**	0.8 (1.2, 27)	1.7 (2.1, 25)	-0.9 (-1.8, 0.1)
Average chest pain at rest (0-10, higher=worse pain)			
Baseline	0.4 (1.4, 32)	0.7 (1.7, 33)	-0.3 (-0.9, 0.5)
24 hours post-surgery	3.9 (2.2, 33)	3.3 (3.3, 32)	0.6 (-0.8, 2.0)
48 hours post-surgery	3.9 (2.1, 31)	4.3 (2.8, 31)	-0.4 (-1.6, 0.9)
72 hours post-surgery	3.2 (25, 2.6)	4.8 (2.8, 25)	-1.6 (-3.2, -0.1)
Discharge	3.1 (2.2, 29)	3.2 (2.5, 32)	-0.1 (-1.3, 1.1)
3 months	1.3 (1.4, 25)	1.5 (2.2, 21)	-0.2 (-1.3, 0.9)
**6 months**	0.4 (0.7, 27)	1.2 (1.7, 25)	-0.8 (-1.6, -0.1)
Average chest pain after coughing (0-10, higher=worse pain)			
Baseline	0.7 (1.9, 32)	0.9 (1.9, 33)	-0.2 (-1.2, 0.7)
24 hours post-surgery	6.2 (2.4, 33)	4.7 (3.3, 32)	1.5 (0.04, 2.9)
48 hours post-surgery	6.4 (2.5, 31)	6.1 (2.6, 31)	0.3 (-1.0, 1.5)
72 hours post-surgery	6.3 (2.6, 25)	7.0 (2.5, 26)	-0.7 (-2.2, 0.7)
Discharge	5.8 (2.2, 29)	5.5 (2.8, 32)	0.4 (-0.9, 1.7)
3 months	2.0 (1.8, 25)	1.95 (2.7, 21)	0.05 (-1.4, 1.4)
**6 months**	1.5 (1.9, 27)	2.1 (2.5, 24)	-0.6 (-1.8, 0.7)
Average chest pain after/with moving (0-10, higher=worse pain)			
Baseline	0.6 (1.9, 32)	0.8 (1.8, 33)	-0.2 (-1.1, 0.7)
24 hours post-surgery	6.1 (2.4, 33)	4.2 (3.3, 32)	1.9 (0.4, 3.3)
48 hours post-surgery	5.9 (2.3, 32)	5.4 (3.0, 31)	0.5 (-0.8, 1.9)
72 hours post-surgery	5.0 (2.6, 24)	6.3 (2.6, 25)	-1.3 (-2.8, 0.2)
Discharge	4.9 (2.2, 31)	4.5 (2.7, 31)	0.4 (-0.9, 1.6)
3 months	1.9 (1.8, 24)	2.2 (3.0, 23)	-0.3 (-1.8, 1.1)
**6 months**	1.0 (1.6, 27)	2.0 (2.7, 26)	-1.0 (-2.2, 0.3)
Average chest pain after physiotherapy (0-10, higher=worse pain)			
Baseline	0.04 (0.1, 29)	0.2 (0.5, 28)	-0.16 (-1.1, 0.7)
24 hours post-surgery	5.4 (2.3, 31)	4.1 (3.2, 30)	1.3 (-0.2, 2.7)
48 hours post-surgery	5.6 (2.3, 32)	5.0 (2.8, 31)	0.6 (-0.8, 1.9)
72 hours post-surgery	4.9 (2.4, 24)	5.9 (2.8, 25)	-1.0 (-2.5, 0.5)
Discharge	4.8 (2.1, 30)	3.8 (2.6 ,30)	1.0 (-0.2, 2.3)
3 months	1.95 (2.0, 18)	2.0 (2.9, 19)	-0.05 (-1.7, 1.6)
**6 months**	1.1 (1.6, 21)	2.0 (2.9, 19)	-0.9 (-2.3, 0.7)
Worst chest pain overall (0-10, higher=worse pain)			
Baseline	0.7 (1.7, 33)	0.9 (2.1, 33)	-0.2 (-1.2, 0.7)
24 hours post-surgery	7.7 (2.0, 33)	6.0 (3.5, 32)	1.7 (0.3, 3.2)
48 hours post-surgery	8.2 (1.6, 32)	7.4 (2.5, 31)	0.8 (-0.3, 1.9)
72 hours post-surgery	7.5 (2.0, 24)	7.9 (2.1, 25)	-0.4 (-1.6, 0.7)
Discharge	7.2 (2.1, 31)	6.6 (2.9, 31)	0.6 (-0.7, 1.9)
3 months	3.5 (2.9, 24)	2.7 (3.4, 23)	0.8 (-1.1, 2.7)
**6 months**	1.9 (2.5, 27)	3.1 (3.4, 26)	-1.2 (-2.8, 0.5)
Worst chest pain at rest (0-10, higher=worse pain)			
Baseline	0.3 (1.2, 33)	0.7 (1.7, 33)	-0.4 (-1.1, 0.4)
24 hours post-surgery	5.7 (2.8, 32)	4.8 (3.6, 32)	0.9 (-0.7, 2.5)
48 hours post-surgery	5.5 (2.7, 32)	5.4 (3.1, 31)	0.1 (-1.4, 1.6)
72 hours post-surgery	4.6 (2.8, 24)	6.1 (3.0, 25)	-1.5 (-3.2, 0.1)
Discharge	4.3 (2.5, 31)	4.7 (2.7, 31)	-0.4 (-1.7, 0.9)
3 months	2.2 (2.3, 24)	1.9 (2.7, 23)	0.3 (-1.2, 1.7)

For peer review only - http://bmjopen.bmj.com/site/about/guidelines.xhtml

**6 months**	1.0 (1.6, 27)	1.9 (2.7, 26)	-0.9 (-2.1, 0.3)
Worst chest pain after coughing (0-10, higher=worse pain)			
Baseline	0.6 (1.6, 33)	1.0 (2.2, 33)	-0.4 (-1.4, 0.6)
24 hours post-surgery	7.2 (1.9, 33)	5.5 (3.4, 32)	1.7 (0.3, 3.1)
48 hours post-surgery	7.6 (2.4, 32)	7.0 (2.5, 31)	0.6 (-0.7, 1.7)
72 hours post-surgery	7.2 (2.5, 25)	7.8 (2.1, 26)	-0.6 (-1.9, 0.7)
Discharge	6.7 (2.3, 30)	5.9 (2.9, 32)	0.8 (-0.5, 2.1)
3 months	2.9 (2.5, 25)	2.8 (3.1, 22)	0.1 (-1.7, 1.7)
**6 months**	1.8 (2.2, 28)	2.5 (2.9, 26)	-0.6 (-2.1, 0.8)
Worst chest pain after/with moving (0-10, higher=worse pain)			
Baseline	0.5 (1.5, 33)	0.9 (1.9, 33)	-0.4 (-1.2, 0.5)
24 hours post-surgery	7.2 (2.1, 33)	5.4 (3.6, 32)	1.8 (0.3, 3.3)
48 hours post-surgery	7.3 (1.9, 32)	6.5 (2.6, 31)	0.8 (-0.3, 2.1)
72 hours post-surgery	6.3 (2.3, 25)	7.0 (2.6, 26)	-0.7 (-2.1, 0.7)
Discharge	6.4 (2.3, 30)	5.8 (2.7, 32)	0.6 (-0.6, 1.9)
3 months	2.3 (1.8, 25)	2.5 (2.8, 22)	-0.2 (-1.6, 1.2)
**6 months**	1.3 (1.8, 28)	2.5 (3.2, 26)	-1.1 (-2.6, 0.4)
Worst chest pain after physiotherapy (0-10, higher=worse pain)			
Baseline	0.1 (0.2, 30)	0.2 (0.6, 29)	-0.1 (-0.4, 0.1)
24 hours post-surgery	6.2 (2.3, 31)	5.0 (3.5, 30)	1.2 (-0.4, 2.7)
48 hours post-surgery	6.5 (2.6, 31)	6.0 (2.8, 31)	0.6 (-0.8, 1.9)
72 hours post-surgery	5.9 (2.6, 25)	6.5 (2.6, 26)	-0.6 (-2.1, 0.8)
Discharge	6.0 (2.3, 29)	4.8 (2.8, 31)	1.2 (-0.1, 2.5)
3 months	2.6 (1.8, 25)	1.9 (2.7, 17)	0.7 (-0.9, 2.3)
**6 months**	1.2 (1.7, 22)	1.8 (2.8, 18)	-0.6 (-2.2, 1.0)

[1] scores<0 indicate less pain with PVB

For peer review only

Table S5: Complications, short-term mortality and length of stay summary

	Day 1		Day 2		Day 3		Between day 3 and discharge/final assessment	
	**PVB**	**TEB**	**PVB**	**TEB**	**PVB**	**TEB**	**PVB**	**TEB**
Arrhythmias	1/34 (3)	2/33 (6)	2/32 (6)	4/32 (13)	1/26 (4)	1/27 (4)	1/34 (3)	3/32 (9)
Systolic blood pressure (<90mmHg)	2/35 (6)	8/33 (24)	2/33 (6)	2/32 (6)	2/27 (7)	2/27 (7)	0/34 (-)	0/32 (-)
Low Respiratory rate (<10/minute)	1/35 (3)	0/33 (-)	0/33 (-)	0/32 (-)	0/27 (-)	0/27 (-)	0/34 (-)	0/32 (-)
Drowsiness	6/34 (18)	4/33 (12)	3/32 (9)	2/32 (6)	0/26 (-)	2/27 (7)	0/34 (-)	1/32 (3)
Nausea & vomiting	8/35 (23)	11/33 (33)	4/33 (12)	4/32 (13)	2/26 (8)	3/27 (11)	5/34 (15)	5/32 (16)
Urinary retention	0/35 (-)	0/33 (-)	1/33 (3)	0/32 (-)	0/27 (-)	0/27 (-)	1/34 (3)	1/32 (3)
Itching	0/34	1/33 (3)	0/32	1/32 (3)	0/26	0/27	0/32	1/32 (3)
High block (above T4)	0/35 (-)	0/33 (-)	0/33 (-)	0/32 (-)	0/27 (-)	0/27 (-)	0/34 (-)	0/32 (-)
Post-dural puncture headache	0/35 (-)	0/33 (-)	0/33 (-)	0/32 (-)	0/27 (-)	0/27 (-)	1/34 (3)	0/32 (-)
Post-op pulmonary complications (PPC)	2/21 (10)	1/19 (5)	2/19 (10)	1/18 (6)	1/15 (7)	1/15 (7)	0/19 (-)	0/19 (-)
Other	20/34 (59)	13/33 (39)	6/32 (19)	7/31 (23)	4/26 (15)	6/27 (22)	3/34 (9)	9/32 (28)
**Level 2 admission**	0/35 (-)	2/33 (6)	-	-	-	-	0/34 (-)	1/32 (3)
If yes, cardiovascular support	-	2/2 (100)	-	-	-	-	-	-
If yes, respiratory support	-	2/2 (100)	-	-	-	-	-	-
If yes, organ support	-	-	-	-	-	-	-	1/1 (100)
If yes, total length of stay *(Median [IQR], n)*		9 [6, 12]						6 [-]
**Level 3 admission**	1/35 (3)	1/32 (3)						
If yes, cardiovascular support	1/1 (100)	1/1 (100)	-	-	-	-	-	-
If yes, respiratory support	1/1 (100)	1/1 (100)	-	-	-	-	-	-
If yes, renal support	0/1 (-)	0/1 (-)	-	-	-	-	-	-
If yes, total length of stay *(Median [IQR], n)*	1 [-]	5 [-]	-	-	-	-	-	-
Discharge with flutter bag	-	-	-	-	-	-	5/34 (15)	4/33 (12)
Mortality	-	-	-	-	-	-	1/35 (3)	1/33 (3)

Frequency (%) presented (unless otherwise stated)

For peer review only

Table S6: Patient satisfaction and blinding questions results summary

		PVB	TEB
Satisfaction with pain therapy after surgery			
Discharge (n=31 for PVB, n=32 for TEB)	Very satisfied	18 (58)	20 (63)
	Satisfied	10 (33)	9 (28)
	Dissatisfied	2 (6)	2 (6)
	Very dissatisfied	1 (3)	1 (3)
3 months (n=26 for PVB, n=23 for TEB)	Very satisfied	13 (50)	9 (39)
	Satisfied	13 (50)	8 (35)
	Dissatisfied	0 (-)	6 (26)
	Very dissatisfied	0 (-)	0 (-)
6 months (n=26 for PVB, n=25 for TEB)	Very satisfied	14 (54)	16 (64)
	Satisfied	11 (42)	6 (24)
	Dissatisfied	0 (-)	2 (8)
	Very dissatisfied	1 (4)	1 (4)
Satisfaction with care provided by hospital			
Discharge (n=31 for PVB, n=32 for TEB)	Very satisfied	22 (71)	22 (69)
	Satisfied	9 (29)	8 (25)
	Dissatisfied	0 (-)	2 (6)
	Very dissatisfied	0 (-)	0 (-)
3 months (n=26 for PVB, n=23 for TEB)	Very satisfied	15 (58)	12 (52)
	Satisfied	11 (42)	9 (39)
	Dissatisfied	0 (-)	2 (9)
	Very dissatisfied	0 (-)	0 (-)
6 months (n=26 for PVB, n=26 for TEB)	Very satisfied	19 (73)	15 (58)
	Satisfied	5 (19)	9 (35)
	Dissatisfied	2 (8)	2 (8)
	Very dissatisfied	0 (-)	0 (-)
Do you know which technique you received?			
Discharge (n=31 for PVB, n=32 for TEB)	TEB	1 (3)	19 (60)
	PVB	8 (26)	3 (9)
	Don't know	22 (71)	10 (31)
3 months (n=26 for PVB, n=22 for TEB)	TEB	2 (8)	12 (55)
	PVB	2 (8)	0 (-)
	Don't know	22 (84)	10 (45)
6 months (n=26 for PVB, n=25 for TEB)	TEB	2 (8)	13 (52)
	PVB	5 (19)	1 (4)
	Don't know	19 (73)	11 (44)

Frequency (%) presented (unless otherwise stated)

For peer review only

CONSORT 2010 checklist of information to include when reporting a randomised trial*

Section/Topic	Item No	Checklist item	Reported on page No
**Title and abstract**			
	1a	Identification as a randomised trial in the title	1
	1b	Structured summary of trial design, methods, results, and conclusions (for specific guidance see CONSORT for abstracts)	2
**Introduction**			
Background and objectives	2a	Scientific background and explanation of rationale	3
	2b	Specific objectives or hypotheses	3
**Methods**			
Trial design	3a	Description of trial design (such as parallel, factorial) including allocation ratio	4
	3b	Important changes to methods after trial commencement (such as eligibility criteria), with reasons	4
Participants	4a	Eligibility criteria for participants	4
	4b	Settings and locations where the data were collected	4
Interventions	5	The interventions for each group with sufficient details to allow replication, including how and when they were actually administered	4-5
Outcomes	6a	Completely defined pre-specified primary and secondary outcome measures, including how and when they were assessed	5
	6b	Any changes to trial outcomes after the trial commenced, with reasons	5
Sample size	7a	How sample size was determined	5
	7b	When applicable, explanation of any interim analyses and stopping guidelines	5-6
Randomisation:			
Sequence generation	8a	Method used to generate the random allocation sequence	4
	8b	Type of randomisation; details of any restriction (such as blocking and block size)	4
Allocation concealment mechanism	9	Mechanism used to implement the random allocation sequence (such as sequentially numbered containers), describing any steps taken to conceal the sequence until interventions were assigned	4
Implementation	10	Who generated the random allocation sequence, who enrolled participants, and who assigned participants to interventions	4
Blinding	11a	If done, who was blinded after assignment to interventions (for example, participants, care providers, those	4

For peer review only - http://bmjopen.bmj.com/site/about/guidelines.xhtml

For peer review only

		assessing outcomes) and how	
	11b	If relevant, description of the similarity of interventions	5
Statistical methods	12a	Statistical methods used to compare groups for primary and secondary outcomes	5-6
	12b	Methods for additional analyses, such as subgroup analyses and adjusted analyses	4
**Results**			
Participant flow (a diagram is strongly recommended)	13a	For each group, the numbers of participants who were randomly assigned, received intended treatment, and were analysed for the primary outcome	Figure 1
	13b	For each group, losses and exclusions after randomisation, together with reasons	Figure 1, 6
Recruitment	14a	Dates defining the periods of recruitment and follow-up	6
	14b	Why the trial ended or was stopped	6
Baseline data	15	A table showing baseline demographic and clinical characteristics for each group	7
Numbers analysed	16	For each group, number of participants (denominator) included in each analysis and whether the analysis was by original assigned groups	Table 2,3,4
Outcomes and estimation	17a	For each primary and secondary outcome, results for each group, and the estimated effect size and its precision (such as 95% confidence interval)	Table 3,4
	17b	For binary outcomes, presentation of both absolute and relative effect sizes is recommended	Table 3,4
Ancillary analyses	18	Results of any other analyses performed, including subgroup analyses and adjusted analyses, distinguishing pre-specified from exploratory	7-12
Harms	19	All important harms or unintended effects in each group (for specific guidance see CONSORT for harms)	12
**Discussion**			
Limitations	20	Trial limitations, addressing sources of potential bias, imprecision, and, if relevant, multiplicity of analyses	12-15
Generalisability	21	Generalisability (external validity, applicability) of the trial findings	12-15
Interpretation	22	Interpretation consistent with results, balancing benefits and harms, and considering other relevant evidence	12-15
**Other information**			
Registration	23	Registration number and name of trial registry	4
Protocol	24	Where the full trial protocol can be accessed, if available	Attached
Funding	25	Sources of funding and other support (such as supply of drugs), role of funders	15

*We strongly recommend reading this statement in conjunction with the CONSORT 2010 Explanation and Elaboration for important clarifications on all the items. If relevant, we also recommend reading CONSORT extensions for cluster randomised trials, non-inferiority and equivalence trials, non-pharmacological treatments, herbal interventions, and pragmatic trials. Additional extensions are forthcoming: for those and for up to date references relevant to this checklist, see www.consort-statement.org.

For peer review only
For peer review only - http://bmjopen.bmj.com/site/about/guidelines.xhtml

BMJ Open

A RANDOMISED CONTROLLED TRIAL TO INVESTIGATE THE EFFECTIVENESS OF THORACIC EPIDURAL AND PARAVERTEBRAL BLOCKADE IN REDUCING CHRONIC POST-THORACOTOMY PAIN (TOPIC) – A PILOT STUDY TO ASSESS FEASIBILITY OF A LARGE MULTI-CENTRE TRIAL

Journal:	*BMJ Open*
Manuscript ID	bmjopen-2018-023679.R1
Article Type:	Research
Date Submitted by the Author:	02-Aug-2018
Complete List of Authors:	Yeung, Joyce ; University of Warwick, Warwick Medical School; Heart of England NHS Foundation Trust, Department of Anaesthesia<br>Middleton, Lee; University of Birmingham, School of Health and Population Sciences<br>Tryposkiadis, Kostas; University of Birmingham, Birmingham Clinical Trials Unit<br>Kerr, Amy; Heart of England NHS Foundation Trust, Department of Thoracic Surgery<br>Daniels, Jane; University of Nottingham, Nottingham Clinical Trials Unit<br>Naidu, Babu; University of Birmingham, Institute of Inflammation and Ageing; Heart of England NHS Foundation Trust, Department of Thoracic Surgery<br>Melody, Teresa; Heart of England NHS Foundation Trust, Academic Department of Anaesthesia, Critical Care, Pain and Resuscitation<br>Goebel, Andreas; University of Liverpool, Institute of Translational Medicine<br>Wilson, Matthew; University of Sheffield, Health Services Research<br>Kumar, Sajith; Heart of England NHS Foundation Trust, Department of Anaesthesia<br>Szentgyorgyi, Lajos; University Hospital of South Manchester NHS Foundation Trust, Department of Anaesthesia<br>Flanagan, Sarah; University of Birmingham, Institute of Applied Health Research<br>Shah, Rajesh; University Hospital of South Manchester NHS Foundation Trust, Department of Thoracic Surgery<br>Worrall, Antony; Heart of England NHS Foundation Trust<br>Gao, Fang; University of Birmingham, Institute of Inflammation and Ageing, College of Medical and Dental Sciences; Heart of England NHS Foundation Trust, Academic Department of Anaesthesia, Critical Care, Pain and Resuscitation
**Primary Subject Heading**:	Anaesthesia
Secondary Subject Heading:	Surgery
Keywords:	Pain management < ANAESTHETICS, Adult anaesthesia <

	ANAESTHETICS, Thoracic surgery < SURGERY, Clinical trials < THERAPEUTICS

SCHOLARONE™
Manuscripts

A RANDOMISED CONTROLLED TRIAL TO INVESTIGATE THE EFFECTIVENESS OF THORACIC EPIDURAL AND PARAVERTEBRAL BLOCKADE IN REDUCING CHRONIC POST-THORACOTOMY PAIN (TOPIC) – A PILOT STUDY TO ASSESS FEASIBILITY OF A LARGE MULTI-CENTRE TRIAL

Authors:

J Yeung[1,2,], L Middleton[3], K Tryposkiadis[3], A Kerr[1], J Daniels[4], B Naidu[1,5], T Melody[1], A Goebel[6], M Wilson[7], S Kumar[1], L Szentgyorgyi[8], S Flanagan[9], R Shah[8], A Worrall[1], and F Gao[1,5], On behalf of TOPIC Investigator Group

[1] Department of Anaesthesia, Heart of England NHS Foundation Trust, Birmingham, United Kingdom

[2] Warwick Medical School, University of Warwick, Coventry, United Kingdom

[3] Birmingham Clinical Trials Unit, University of Birmingham, Birmingham, United Kingdom

[4]Nottingham Clinical Trials Unit, University of Nottingham, Nottingham, United Kingdom

[5]Institute of Inflammation and Ageing, University of Birmingham, Birmingham, United Kingdom

[6]University of Liverpool and Walton Centre NHS Foundation Trust, Liverpool, United Kingdom

[7]School of Health and Related Research, University of Sheffield, Sheffield, United Kingdom

[8]Manchester University NHS Foundation Trust, Manchester, United Kingdom

[9]Institute of Applied Health Research, University of Birmingham, Birmingham, United Kingdom

Corresponding author: Fang Gao

E-mail: f.gaosmith@bham.ac.uk Tel: 01214147483

Institute of Inflammation and Ageing, College of Medical and Dental Sciences, University of Birmingham, Birmingham B15 2TT, United Kingdom

Word Count 3618

For peer review only

ABSTRACT

Objectives: Thoracotomy is considered one of the most painful surgical procedures. The incidence of chronic post-thoracotomy pain (CPTP) is up to 50%. Paravertebral blockade (PVB) may be superior to thoracic epidural blockade (TEB) in preventing CPTP.

Design: A randomised, parallel, external pilot study was conducted to assess whether a large randomised trial of TEB and PVB with CPTP as the primary outcome is feasible.

Setting: Two adult thoracic centres in the UK

Participants: All adult patients admitted for elective open thoracotomy. Participants were excluded if they were ASA physical status IV or V; or if there is contraindication to local anaesthetics; infection near the proposed puncture site; coagulation/thoracic spine disorders; required chest wall resection or emergency thoracic surgery or had a previous thoracotomy.

Results: All patients presenting for thoracotomy were screened over a 12-month period with 194 found to be eligible. Of these, 69 (36%) were randomised (95%CI: 29%-42%). Discounting five participants who died, 54 of 64 participants (84%) returned questionnaire booklets at six months. The number of participants indicating at least a moderate level of chest pain at six months was lower with PVB but with high levels of uncertainty (RR: 0.7; 95%CI: 0.3-1.7 for worst pain; RR: 0.3; 95%CI: 0.0-2.8 for average). There were no safety concerns.

Conclusions: A large multicentre RCT of PVB versus TEB is feasible as it is possible to randomise and follow up participants with high fidelity. Pain scores were lower on average with PVB compared to TEB but a much larger trial is required to confirm this reliably.

Trial Registration: ISRCTN45041624

For peer review only

Strengths and limitations of this study

- This randomised, external, pilot trial had pre-planned feasibility thresholds to assess whether a large trial is feasible.
- Selected two sites represented the national thoracic and anaesthetic cohort, providing best representation of full trial national recruitment
- Consultant anaesthetists in both participating sites had adequate training and appropriate assessment for the competence of each technique.
- The study was not designed and powered to be large or long enough to provide convincing evidence to support the use of either TEB or PVB as intraoperative anaesthetic technique, but provides evidence that a larger, substantive trial is feasible.

For peer review only

BACKGROUND

An estimated 7200 thoracotomies (surgical incision into the chest wall) are performed annually in the UK, most commonly to treat lung cancer.[1] Thoracotomy is considered one of the most painful surgical procedures due to tissue, muscle and nerve damage from the incision and wound retraction. Intercostal nerve injury can result in a high risk of persistent pain for months after surgery.[2-4] The incidence of chronic post-thoracotomy pain (CPTP; defined as pain that recurs or persists at least two months following the surgery)[5] is thought to be as high as 50%.[6]

Two main analgesic techniques are commonly used for perioperative pain control during thoracotomy. Thoracic epidural blockade (TEB) blocks nerves that supply the chest with local anaesthetic bilaterally, at spinal cord level. It acts by reducing onward transmission of painful nerve signals but may not abolish them completely.[7,8] Paravertebral blockade (PVB), involves injecting local anaesthetic into the paravertebral space on the side of surgery. There are studies which describe the spread of PVB injections anteriorly across the heads and necks of the ribs to the spaces above and below; medially through an intervertebral foramen or spread laterally in the intercostal plane.[9] However PVB has the potential to completely block painful nerve signals from reaching the spinal cord.[9,10] This total blockade of nerve signals could remove the stimulus for 'central sensitisation' which underpins the formation of chronic pain pathways. PVB could be uniquely effective in preventing long-term pain[11], and there is evidence from a recent trial of two techniques in breast surgery to support this premise.[12] With limited current evidence to support the most effective choice of anaesthetic technique in preventing CPTP, current UK practice varies greatly.[13] A recent Cochrane Review recommended that a high quality randomised controlled trial (RCT) to compare TEB and PVB with the primary outcome of chronic pain is urgently needed.[14]

The overall aim of this research is to determine whether PVB at thoracotomy is more effective in reducing CPTP compared to TEB. To answer this question a large, multi-centre RCT is required. The specific aim of this pilot study was to assess the feasibility of conducting a larger trial and to enhance the likelihood of its success by developing the necessary structure and processes that a large trial would need. Objectives included: an assessment of the effectiveness of the patient identification and screening process; identification of reasons for failure to randomise; development of educational and training materials for surgeons and anaesthetists; evaluation of robustness of in-hospital data collection processes; assessment of trial processes including impact on participants and staff.

METHODS

For peer review only

We conducted a randomised, parallel group, external pilot trial of TEB versus PVB for perioperative pain control during thoracotomy. Recruitment took place over 12 months in two adult thoracic centres: Heartlands Hospital, Birmingham and University Hospital South Manchester (Wythenshawe). This study was approved by NHS Research Ethics Committee (NRES Committee East Midlands - Derby, REC number 14/EM/1280) and was prospectively registered (ISRCTN45041624).

Population and inclusion/exclusion criteria

Eligible participants were all adults admitted for an elective open thoracotomy. Participants were excluded if they had any of the following: ASA (classification of fitness) physical status IV or V (as these patients are unlikely to present for elective surgery due to their physical condition); contraindication to local anaesthetics; infection near the proposed puncture site; coagulation abnormalities (in accordance with Association of Anaesthetists in Great Britain and Ireland guidance 2013) ; thoracic spine disorders; required chest wall resection or emergency thoracic surgery or had a previous thoracotomy (scarring due to previous surgery can limit the effectiveness of paravertebral block and these patients may already have existing chronic pain).

Study conduct

All patients thought to fulfil the eligibility criteria were approached with study information at their pre-operative assessment clinic. They were provided with a Patient Information Sheet and given the opportunity to consider participation. Once eligibility was confirmed and written informed consent was obtained, randomisation was performed prior to surgery using a web-based central randomisation system (via Birmingham Clinical Trials Unit) to allocate patients to either TEB or PVB in a 1:1 ratio. Minimisation was used to achieve balance between sex, age (<65 years or ≥65 years), thoracotomy for lung cancer resection or for other indication and study site. Research nurses at each site was responsible for randomisation and assigned group allocation was revealed to responsible anaesthetist when patients arrived at the operating theatre. An Oversight Committee was formed to provide independent guidance to the Trial Management Committee and to review accruing safety information during the period of recruitment. Public and patient involvement was integral to the study throughout.

Interventions

Interventions were delivered by experienced thoracic anaesthetists, trained and deemed competent in both anaesthetic techniques. Participants were not told specifically about their allocated intervention. It was not possible to blind anaesthetists, surgeons or nursing staff due to the nature of the interventions. PVB was performed using three single injections pre-incision with the patient awake or asleep, at the levels of T3-4, 5-6 and 7-8 with 15ml 0.25% levobupivicaine/bupivacaine,

For peer review only

using a landmark technique at each level. The PVB catheter was then placed at T5 under direct vision by a surgeon during surgery. A loading dose of 10ml 0.25% levobupivicaine/bupivacaine was administered before chest closure, followed by infusion of 0.125% levobupivicaine/bupivacaine 0.1-0.25ml kg^{-1} hr^{-1} until operation end. TEB was inserted pre-incision with the patient awake or asleep, with a catheter inserted at the spinal level supplying the skin at the incision site (normally T5-6), a test dose of 3ml of 0.5% bupivacaine, and a loading dose of 0.25% levobupivicaine/bupivacaine 0.1ml kg^{-1} with up to 3mg of diamorphine. This was followed by infusion of 0.125% levobupivicaine/bupivacaine with 2µg ml^{-1} fentanyl at 0.1-0.25 ml kg^{-1} hr^{-1}. Further information on delivery and training on the interventions is available in the study protocol.[15] Identical dressings and pumps were used for both interventions to maintain group allocation.

Pilot outcomes

The following outcomes and targets were set a-priori as being indicative that a larger trial would be feasible to conduct.[15] These were: i) patient recruitment rate (the proportion of eligible patients randomised) at least 25%; ii) screening rate (the proportion of all patients listed for elective thoracotomy that were screened for eligibility and recorded on a screening log) at least 90%; iii) clinicians' willingness to recruit: national survey of consultant thoracic anaesthetist indicating the number willing to participate in a larger study at least 70%; iv) data completion – number of anaesthetic/peri-operative forms completed at least 90% and follow-up questionnaires completed at six months at least 80%. The study was given a 12-month recruitment period, after which screening would stop.

Clinical and participant reported outcome measures

Baseline data (including participant completed questions as indicated below) were recorded at the pre-operative assessment appointment. Intraoperative (anaesthetic technique and use), post-operative and discharge data (analgesic use, pain scores on days 1-3, acute complications, mortality and length of stay) were collected by the study team. Three and six-month post-randomisation follow-up was conducted by postal questionnaire. This included: Visual Analogue Scales (VAS)[16] for worst and average chest pain (overall, at rest, after coughing, after moving, after physiotherapy; with higher score - maximum 10 - indicating higher levels of pain, as marked along a 10cm line), Brief Pain Inventory (BPI)[17], Neuropathic Pain Scale (NPS)[18], generic health-related quality of life (EQ-5D-5L)[19], Hospital Anxiety and Depression Scale (HADS)[20] and assessment of satisfaction and participant blinding. Serious adverse events were collected throughout. Patient outcome data was collected by research team member who was blinded to the assigned group.

Statistics

For peer review only

We expected to recruit between 50-75 participants over a 12-month recruitment period, depending on the number found to be eligible. We estimated that there would be approximately 500 open elective thoracotomies from the two sites, of which 60% would potentially be eligible (300). Using our own target criteria of 25% recruited would yield 75 participants. This number would be sufficient to measure the recruitment rate to uncertainty width up to approximately 10%.

Feasibility outcomes were considered with simple summary statistics (proportions and percentages), with uncertainty estimates provided by 95% confidence intervals. Clinical and participant reported outcome measures were analysed with point estimates (RR or mean difference) and 95% confidence intervals, adjusting for the minimisation variables. They were not subject to hypothesis testing as the size of the sample would not allow reliable assessment of efficacy.[21] Participants were considered in the groups they were randomised to regardless of compliance (intention-to-treat).

Qualitative assessment and survey of practice

In addition to the clinical assessment, semi-structured qualitative interviews were undertaken with 18 patients at 6-8 weeks discharge to gauge the impact of the trial on their hospital care and eight clinical staff (four anaesthetists, one surgeon and three members of the research team) to aid insight into trial related processes. An electronic national survey of anaesthetists on willingness to participate in a future trial was also conducted via the Association for Cardiothoracic Anaesthesia and Critical Care membership in 26 UK thoracic units.

Patient involvement

Our study question in chronic pain post thoracotomy is in the top ranked research priorities voted by patient 'What can we do to stop patients developing chronic pain after surgery?' The development of study protocol was designed in conjunction with our patient partners who also participated as a member of trial management group and trial steering committee. Our patient partners reviewed study design, paying attention to acceptability of randomisation analgesic techniques in perioperative setting and burden of study participation. Study results including plan for future study has been disseminated within local and national patient research ambassador groups.

RESULTS

Participants and follow-up

564 patients were screened for eligibility from July 2015 to July 2016 - 100% of those listed for elective thoracotomy - with 194 patients found to be eligible (Figure 1). The most common reasons for ineligibility were: undergoing video-assisted thoracoscopic surgery (174/370, 47%); participation

For peer review only

in another study which did not allow co-enrolment (59/370, 16%); previous thoracotomy (31/370, 8%). Of the 194 patients, 69 (36%, 95%CI: 29% to 42%) were ultimately randomised. Of the others, the most common reasons for non-randomisation were: anaesthetists trained to perform PVB not available (24/125, 19%); did not want to be randomised (15/125, 12%); patient had preference for PVB (15/125, 12%). Details of randomised participants are given in Table 1. The minimisation algorithm provided appropriate balance for the balancing factors; the average age of participants was 66 years with the vast majority were having a lung cancer resection operation (60/69, 87%). Seven participants (10%) reported previous problems with (unspecified) chronic pain.

At six months post-randomisation, 54 of 63 (86%) questionnaire booklets were returned, discounting 5 participants who died during the follow-up period and one participant who withdrew from the study. Of these, only one did not complete their VAS questions.

For peer review only

Table 1. Baseline characteristics of patients

		PVB (n=35)	TEB (n=34)
Age >=65, years[1]		21 (60)	20 (59)
Age, years	Mean (SD)	66.0 (10.6)	65.9 (7.9)
Gender=male[1]		19 (54)	20 (59)
Centre[1]	Birmingham Heartlands	23 (66)	26 (76)
	University Hospital South Manchester	12 (34)	8 (24)
Reason for thoracotomy=lung cancer resection[1]		30 (86)	30 (88)
ASA[2] physical status	1: Normal healthy	0 (-)	2 (6)
	2: Mild systemic disease	13 (37)	10 (30)
	3: Severe systemic disease	22 (63)	21 (64)
	Missing	-	1
ECOG[3] performance status	0: Normal activity	20 (57)	19 (58)
	1: Symptomatic but nearly fully ambulatory	14 (40)	14 (42)
	2: Symptomatic but bed <50% daytime	1 (3)	0 (-)
	3: Symptomatic bed>50% daytime	0 (-)	0 (-)
	4: Unable to get out of bed	0 (-)	0 (-)
	Missing	-	1
Dyspnoea	0: None	17 (48)	17 (52)
	1: Slight	16 (46)	13 (39)
	2: Moderate	2 (6)	1 (3)
	3: Moderately severe	0 (-)	2 (6)
	4: Severe	0 (-)	0 (-)
	5: Very severe	0 (-)	0 (-)
	Missing	-	1
Smoking status	Never smoked	7 (20)	6 (18)
	Stopped>1 year	19 (54)	18 (55)
	Stopped>6 weeks	3 (9)	3 (9)
	Stopped<6 weeks	1 (3)	4 (12)
	Current smoker	5 (14)	2 (6)
	Missing	-	1
Smoking-pack years	Median [IQR]	37 [20, 49]	37 [20, 50]
	Missing	-	1
Alcohol units per week	Median [IQR]	3 [0, 13.5]	0 [0, 9.7]
	Missing	-	1
Previous medical history=yes	COPD	7 (20)	6 (18)
	Ischaemic heart disease	4 (11)	3 (9)
	Congestive cardiac failure	0 (-)	1 (3)
	Hypertension	18 (51)	15 (45)
	Diabetes diet controlled	0 (-)	3 (9)
	Diabetes oral medication	6 (17)	3 (9)
	Diabetes insulin controlled	2 (6)	1 (3)
	Renal failure	1 (3)	1 (3)
	Previous stroke	0 (-)	0 (-)
	Hyperthyroidism	0 (-)	0 (-)
	Hypothyroidism	5 (14)	2 (6)
	Other cancer	12 (34)	7 (21)
	Chronic pain	2 (6)	5 (15)
	Missing	-	1
BMI, kg/m^2	Mean (SD)	28.4 (5.8)	27.7 (5.9)
	Missing	-	1

Frequency (%) presented (unless otherwise stated)
[1] minimisation variable; [2] American Society of Anesthesiologists; [3] Eastern Cooperative Oncology Group

For peer review only

Analgesic technique

Of the 69 randomised, 67 (97%) received their randomised intervention. One participant allocated PVB received TEB as the pleura was removed and the surgeon was unable to place the PVB. Another participant was not considered fit for surgery and was withdrawn from the study. Discounting this participant, all anaesthetic technique and intra-operative forms were completed. A summary of the anaesthetic techniques is given in Table 2; a summary of the operative details and other analgesics provided to participants up to discharge is provided in the Appendix (Tables S1, S2).

Table 2. Anaesthetic technique summary

		**PVB (n=35)**	**TEB (n=33)[1]**
Difficulty in insertion		4 (12)[2]	14 (42)
Failure to deliver technique		1 (3)	0 (-)
Intraoperative use			
Preoperative bolus given		26 (74)	25 (76)
Bolus drug given through catheter		14 (40)	24 (73)
Infusion given through catheter		23 (66)	21 (64)
Post-operative settings			
Local anaesthetic was Bupivacaine (as opposed to Levobupivacaine)		32 (94)[2]	19 (58)
Concentration	0.1%	20 (59)[2]	11 (33)
	0.125%	4 (12)[2]	22 (67)
	0.25%	10 (29)[2]	0 (-)
Fentanyl	None	32 (91)	3 (9)
	2 mcg/ml	0 (-)	0 (-)
	4 mcg/ml	3 (9)	30 (91)
Starting rate, ml/hr	Median [IQR]	20 [10, 20][2]	6 [6, 8]
Prescribed range, ml/hr	Median [IQR]	20 [15, 20][2]	12 [8, 15]

Frequency (%) presented (unless otherwise stated)

[1] one participant received neither technique as they did not have an operation; [2] one response missing (n=34)

For peer review only

Patient reported outcomes

Levels of pain in the three days post-operation appeared similar in both groups (Appendix Table S3). The number of patients indicating at least a moderate level of average or worst chest pain (based on commonly used threshold of >3 or ≥4[22, 23]) was lower with PVB compared to TEB at six months, but with expected high levels of uncertainty given the limited size of sample (Table 3; full results given in Appendix Table S4). Results from other questionnaire responses had a lot of uncertainty (Table 4).

Table 3. Incidence of significant (>3 or >=4) or severe pain (>=7) from Visual Analogue Scale Scores

	PVB n (%)	TEB n (%)	RR (95% CI) [1]
Average chest pain overall>3			
3 months	5/25 (20.0)	6/21 (28.6)	0.7 (0.2, 2.0)
**6 months**	2/27 (7.4)	5/25 (20.0)	0.4 (0.1, 1.7)
Average chest pain overall>=4			
3 months	5/25 (20.0)	4/21 (19.0)	1.1 (0.3, 3.4)
**6 months**	1/27 (3.7)	3/25 (12.0)	0.3 (0.03, 2.8)
Average chest pain overall>=7			
3 months	0/25 (-)	2/21 (10.0)	- [2]
**6 months**	0/27 (-)	1/25 (4.0)	- [2]
Worst chest pain overall>3			
3 months	9/24 (37.5)	7/23 (30.4)	1.2 (0.6, 2.8)
**6 months**	7/27 (25.9)	11/26 (42.3)	0.6 (0.3, 1.3)
Worst chest pain overall>=4			
3 months	8/24 (33.3)	7/23 (30.4)	1.1 (0.5, 2.5)
**6 months**	7/27 (25.9)	9/26 (34.6)	0.75 (0.3, 1.7)
Worst chest pain overall>=7			
3 months	6/24 (25.0)	5/23 (21.7)	1.2 (0.4, 3.3)
**6 months**	2/27 (7.4)	5/26 (19.2)	0.4 (0.1, 1.8)

[1] RR<0 indicate less incidence with PVB; [2] unable to estimate

For peer review only

Table 4. Results of patient reported outcomes

	PVB mean score (SD, n)	TEB mean score (SD, n)	Difference between groups (95% CI)
**Brief pain inventory (BPI) results summary[1]**			
Pain severity score (0-10, higher=worse pain)			
Baseline	0.9 (1.7, 34)	1.3 (2.0, 32)	-0.4 (-1.3, 0.5)
24 hours post-surgery	5.3 (1.7, 33)	3.9 (2.7, 32)	1.4 (0.3, 2.5)
48 hours post-surgery	5.2 (1.7, 30)	5.1 (2.5, 31)	0.1 (-0.9, 1.2)
72 hours post-surgery	4.9 (1.7, 23)	5.6 (2.0, 25)	-0.7 (-1.8, 0.4)
Discharge	4.0 (1.4, 31)	4.1 (2.4, 32)	-0.1 (-1.1, 0.9)
3 months	1.6 (1.7, 22)	2.5 (2.5, 21)	0.9 (-2.2, 0.4)
**6 months**	1.3 (1.8, 25)	2.3 (2.6, 23)	-1.0 (-2.3, 0.3)
Pain interference score (0-10, higher=worse pain)			
Baseline	0.6 (1.3, 34)	1.5 (2.2, 32)	-0.9 (-1.8, -0.1)
24 hours post-surgery	5.8 (2.1, 30)	5.4 (2.9, 28)	0.4 (-0.8, 1.8)
48 hours post-surgery	6.0 (2.0, 30)	5.8 (2.2, 27)	0.2 (-0.9, 1.3)
72 hours post-surgery	5.4 (2.3, 21)	5.8 (2.6, 23)	-0.4 (-1.9, 1.1)
Discharge	4.7 (2.0, 23)	4.3 (2.5, 29)	0.4 (-0.9, 1.7)
3 months	1.1 (1.4, 24)	2.8 (3.0, 22)	-1.7 (-3.1, -0.3)
**6 months**	1.6 (2.2, 27)	1.9 (2.5, 25)	-0.3 (-1.6, 1.0)
**Neuropathic pain scale (NPS) results summary[1]**			
How intense does your pain feel? (0-10, higher=worse pain)			
Baseline	1.7 (2.8, 33)	1.8 (2.8, 33)	-0.1 (-1.5, 1.3)
Discharge	5.8 (2.8, 31)	5.4 (3.8, 31)	0.4 (-1.3, 2.1)
3 months	2.2 (2.4, 25)	3.6 (3.8, 21)	-1.4 (-3.4, 0.5)
**6 months**	2.2 (2.7, 27)	2.7 (3.2, 26)	-0.5 (-2.2, 1.1)
How sharp does your pain feel? (0-10, higher=worse pain)			
Baseline	1.1 (2.8, 33)	1.6 (3.4, 32)	-0.5 (-2.1, 1.1)
Discharge	5.5 (3.2, 31)	5.4 (4.0, 31)	0.1 (-1.7, 1.9)
3 months	2.6 (3.0, 25)	3.2 (4.0, 21)	-0.6 (-2.8, 1.5)
**6 months**	3.0 (3.3, 27)	2.4 (3.3, 26)	0.6 (-1.3, 2.4)
How hot does your pain feel? (0-10, higher=worse pain)			
Baseline	1.0 (2.6, 33)	1.3 (2.7, 32)	-0.3 (-1.6, 1.0)
Discharge	3.6 (3.4, 31)	2.4 (2.9, 31)	1.2 (-0.4, 2.8)
3 months	1.6 (2.8, 25)	2.5 (3.3, 21)	-0.9 (-2.7, 1.0)
**6 months**	1.2 (2.0, 28)	1.6 (2.9, 26)	-0.4 (-1.7, 1.0)
How dull does your pain feel? (0-10, higher=worse pain)			
Baseline	1.8 (2.9, 33)	1.5 (2.5, 33)	0.3 (-1.0, 1.7)
Discharge	2.9 (2.7, 30)	3.9 (2.8, 30)	-1.0 (-2.4, 0.5)
3 months	2.6 (2.1, 25)	3.3 (3.3, 21)	-0.7 (-2.4, 0.9)
**6 months**	2.1 (2.8, 28)	2.3 (2.8, 26)	-0.2 (-1.8, 1.2)
How cold does your pain feel? (0-10, higher=worse pain)			
Baseline	0.3 (1.6, 33)	0.4 (1.7, 32)	-0.1 (-0.9, 0.7)
Discharge	1.5 (2.5, 30)	1.0 (2.2, 31)	0.5 (-0.7, 1.7)
3 months	0.2 (0.5, 25)	1.5 (2.9, 21)	-1.3 (-2.7, -0.0)
**6 months**	0.3 (0.9, 28)	0.7 (1.6, 26)	-0.4 (-1.1, 0.3)
How sensitive is your pain? (0-10, higher=worse pain)			
Baseline	0.5 (1.9, 33)	1.1 (2.5, 32)	-0.6 (-1.7, 0.5)
Discharge	2.3 (2.9, 30)	2.8 (2.9, 32)	-0.5 (-1.9, 1.0)
3 months	2.2 (3.0, 25)	2.1 (2.7, 23)	0.1 (-1.6, 1.7)
**6 months**	1.6 (2.0, 28)	2.5 (3.1, 26)	-0.9 (-2.4, 0.5)
How itchy is your pain? (0-10, higher=worse pain)			
Baseline	0 (0, 33)	0.8 (2.0, 32)	-0.8 (-1.5, -0.0)
Discharge	2.2 (2.8, 30)	2.6 (3.3, 31)	-0.4 (-2.0, 1.2)
3 months	1.2 (2.5, 25)	1.8 (2.6, 22)	-0.6 (-2.1, 0.9)
**6 months**	0.4 (0.7, 28)	2.1 (2.6, 25)	-1.7 (-2.8, -0.6)
How unpleasant is your pain? (0-10, higher=worse pain)			
Baseline	1.9 (2.9, 31)	2.1 (3.3, 33)	-0.2 (-1.7, 1.4)
Discharge	6.2 (3.0, 30)	5.6 (3.3, 30)	0.6 (-1.1, 2.2)
3 months	2.8 (2.6, 24)	4.0 (3.9, 21)	-1.2 (-3.3, 0.8)

For peer review only

**6 months**	2.4 (2.7, 28)	3.0 (3.2, 24)	0.6 (-2.3, 1.0)
How intense is your deep pain? (0-10, higher=worse pain)			
Baseline	1.3 (2.4, 32)	2.4 (3.7, 33)	-1.1 (-2.6, 0.5)
Discharge	6.2 (2.8, 31)	5.5 (3.4, 31)	0.7 (-0.9, 2.3)
3 months	3.0 (2.8, 23)	4.1 (3.7, 21)	-1.1 (-3.1, 0.9)
**6 months**	2.8 (3.0, 28)	2.5 (2.9, 25)	0.3 (-1.4, 1.9)
How intense is your surface pain? (0-10, higher=worse pain)			
Baseline	0.8 (2.1, 31)	0.7 (1.5, 32)	0.1 (-0.9, 1.0)
Discharge	3.9 (3.4, 30)	3.8 (3.3, 32)	0.1 (-1.7, 1.7)
3 months	1.9 (2.2, 24)	3.5 (3.6, 21)	-1.6 (-3.3, 0.2)
**6 months**	1.2 (1.5, 27)	2.7 (3.2, 24)	-1.5 (-2.9, -0.1)
**EQ-5D-DL results summary[2]**			
EQ-5D index score (-0.59=worst outcome, 1.0=best outcome)			
Baseline	0.75 (0.22, 35)	0.77 (0.20, 33)	-0.02 (-0.12, 0.08)
Discharge	0.49 (0.23, 27)	0.49 (0.23, 30)	-0.00 (-0.12, 0.12)
3 months	0.73 (0.17, 25)	0.65 (0.23, 23)	0.08 (-0.04, 0.20)
**6 months**	0.72 (0.23, 25)	0.69 (0.22, 25)	0.03 (-0.09, 0.16)
EQ-5D thermometer (0-100, higher=better)			
Baseline	76.6 (16.3, 34)	74.3 (17.8, 33)	2.3 (-5.9, 10.7)
Discharge	60.4 (22.7, 26)	60.2 (20.7, 30)	0.2 (-11.5, 11.9)
3 months	73.2 (21.2, 25)	67.5 (18.3, 23)	5.7 (-5.8, 17.2)
**6 months**	78.0 (14.0, 25)	64.4 (20.4, 25)	13.6 (3.6, 23.6)
**Hospital anxiety and depression index score (HADS) results summary[3]**			
Depression index score (0-21, lower=better)			
Baseline	2.9 (2.9, 33)	3.0 (2.6, 32)	-0.1 (-1.4, 1.3)
Discharge	4.3 (3.4, 28)	6.0 (5.0, 28)	-1.7 (-4.0, 0.5)
3 months	3.6 (2.7, 25)	5.5 (4.0, 21)	-1.9 (-4.1, 0.1)
**6 months**	4.5 (3.5, 24)	6.0 (4.6, 25)	-1.5 (-3.9, 0.8)
Anxiety index score (0-21, 0-lower=better)			
Baseline	6.3 (5.0, 34)	6.5 (4.4, 30)	-0.2 (-2.6, 2.1)
Discharge	5.0 (4.4, 29)	7.0 (5.3, 28)	-2.0 (-4.6, 0.6)
3 months	5.2 (4.3, 25)	6.1 (4.8, 19)	-0.9 (-3.7, 1.9)
**6 months**	5.7 (3.6, 24)	7.3 (5.0, 25)	-1.6 (-4.1, 0.9)

[1] scores<0 indicate less pain with PVB

[2] scores>0 indicate better QoL with PVB

[3] scores<0 indicate less anxiety/depression with PVB

For peer review only

Safety

No concerns were expressed by the independent Oversight Committee who met twice during the recruitment period to review study progress and safety data. Complications and short-term mortality data appeared similar in both groups (Appendix Table S5). There were five deaths; only one was considered to be a serious adverse event, this was aspiration pneumonitis and was not considered related to intervention (PVB). One other participant experienced a serious adverse event in the TEB group; this participant was diagnosed with acute kidney injury in the post-operative period.

Assessment of participant blinding

19/32 (60%) patients in the TEB group correctly identified the anaesthetic technique they had been allocated at discharge; the figure for PVB was 8/31 (26%); this reduced to 13/24 (52%) in the TEB group at 6 months and 5/26 (19%) in the PVB group (Appendix Table S6).

National survey of anaesthetists

Forty-three responses were returned. Of these, 27 anaesthetists indicated that they would be willing to randomise in a future study (63%). Five (12%) indicating that they would ‘possibly’ be willing to randomise with reasons of concern given as lack of skill and ward structure. The remaining 11 (26%) were unwilling.

Qualitative interviews

Interviewed participants had a positive experience, reporting that they felt well informed by trial staff. The consent process was felt to be well undertaken and patients felt reassured by the explanations of randomisation. A substantial number of participants reported that the study questionnaires were long and repetitive. Interviewed staff felt recruitment was successful although there were some challenges when the role was undertaken by non-clinical staff. There was a feeling that some of the data collection tools were repetitive. Clinical staff were supportive of the trial; they found that the trial processes, randomisation and procedures were very straightforward. The guidance and teaching provided for anaesthetists to get them up to speed for performing PVB was very positively received and it was considered a relatively simple procedure to learn.

DISCUSSION

Our four key indicators for feasibility were met and we have shown that a large multicentre randomised controlled trial of PVB versus TEB for peri-operative pain control with an objective of

For peer review only

assessing long-term post-thoracotomy chronic pain is feasible. It is possible to randomise and follow up patients with high fidelity over six months. In the qualitative assessment we carried out, participants and staff were largely happy with study processes. In particular, the consent, randomisation and assessment methods were considered appropriate. As hypothesised, VAS pain scores were lower with PVB compared with TEB at six months on average but with high levels of uncertainty. This would need to be investigated further in a much more substantial trial.

Whilst the evidence on short-term outcomes, e.g. minor complications and analgesic efficacy, point to PVB being at least as effective as TEB[14], current evidence on whether PVB is more effective than TEB in reducing rates of chronic pain is limited. A recent systematic review failed to attain sufficient data to compare the impact of TEB and PVB techniques on CPTP. [14] The incidence of CPTP was very poorly reported and studies did not describe how data on chronic pain was measured or collected. The lack of evidence highlights an area in need of further research. We are not aware of any current or planned randomised trials with CPTP as an outcome that may change these findings since this systematic review was published. Incidence of CPTP was slightly lower in our study - between 30-34% - than previously quoted at 49%[6], although we need to acknowledge that our figure is associated with a reasonably large amount of uncertainty given the small size of sample. A likely contributing factor to this discrepancy is the considerable and widely acknowledged variation in how pain is measured[24], as it is dependent on the measurement instrument and threshold chosen amongst other factors.[22]

This feasibility work has allowed us to fine tune study processes ahead of a larger trial. One likely modification to the future study protocol is to revise the length of the follow-up questionnaires, it was considered by participants that their length was too long and repetitive in content. Shortened and simplified follow-up questionnaire would also potentially helping to improve follow-up figures further, as lengthy questionnaires are a known predictor of participant drop-out from trials.[25]

Inclusion and exclusion criteria appeared appropriate and do not appear to need to be changed. The vast majority of ineligible participants were those scheduled to receive video-assisted thoracoscopic surgery (VATS). Whilst this procedure is becoming more popular[1], the comparison in question is not an appropriate one as TEB is not routinely used to provide pain relief for in this type of surgery. The VATS approach also does not require any rib spreading, resulting in a less invasive procedure, and whilst chronic pain is still an issue after VATS it is not considered to be as great as following open thoracotomy.[26] Unfortunately, whilst VATS is becoming more common, there will still be a substantial proportion of patients that will always be ineligible for this operation because of, for example, inability to achieve complete resection in more advanced pulmonary lung cancers. Lung cancer remains the most common cancer worldwide.[27,28]

For peer review only

It was apparent from the assessment of the ability to blind the participant to the analgesic technique that complete blinding is unlikely to be possible. Substantially more participants appeared to be able to determine they had had an epidural blockade than the paravertebral technique. Attempts to completely blind participants in any future study are likely to make processes prohibitively complex and expensive due to innate differences in the mode of delivery and action in the two analgesic techniques. This could include sham insertion techniques to disguise the randomised intervention. The question is whether patient blinding is likely to be an important potential cause of detection bias, unduly affecting the results of any future trial. The proposed primary outcome of pain rating is subjective in nature and this is a theoretical possibility. However, we believe that there is no reason to suspect that recipients of the randomised interventions have strong pre-conceptions with regard to the relative effectiveness of each analgesic technique. Furthermore, the primary outcome would be collected via questionnaires administered by post or phone, at a time remote from the original operative procedure, which are likely to be resilient to the effects of imperfect concealment.

Findings from the survey of practice were encouraging, although whilst we set a target of 70% of thoracic anaesthetists willing to randomise to a future trial as being indicative of success, we found 63% willing and 12% 'possibly willing'. To achieve 70%, we would require solutions for those anaesthetists expressing uncertainty. The major reasons given were lack of skill and ward structure. These hurdles do not appear insurmountable if funding for further training and extra nurse support could be provided; something a large funding body may be willing to provide. A successful trial would also require full engagement with anaesthetic and thoracic surgical trainees' network to maximise recruitment.

We have reported the study to randomised pilot study standards as recommended by CONSORT extension for pilot study.[29] Also as recommended[30], we set objectives a priori to determine whether the pilot study would demonstrate feasibility of a larger trial. Since this study was not a definitive trial, we have been careful not to over-interpret the findings. Hence at this stage, we cannot expect that the outcomes of this pilot study to be directly translated into clinical care, as the study was not large enough to be able to detect small to moderate realistic-sized difference in rates of chronic pain, nor was the scope wide enough in terms of the number of centres involved to return a generalisable result.

Nevertheless, this pilot study is an important precursor to a larger, substantive, trial and provides invaluable information that will help to ensure its success. A definitive study - instigated by the authors of this manuscript - has now been funded by the NIHR Health Technology Assessment Programme and is expected to start recruiting in 2018 from 20 UK thoracic centres. The design of this study has been partially informed by the results of this pilot. The primary outcome will be

For peer review only - http://bmjopen.bmj.com/site/about/guidelines.xhtml

incidence of chronic pain at six months attained from visual analogue scale responses similar to that used here; other outcomes will follow IMMPACT recommendations.[31] A total sample size of 1026 participants will give 90% power (p=0.05) to detect a reduction in incidence of CPTP of 30% with TEB (a similar event rate to that noted in this pilot study) down to 20% with PVB. The rate of recruitment noted in this study has been factored into the number of sites and length of the recruitment period required; we estimate access to a pool of 5000 participants over a two and a half year recruitment period and need to recruit a fifth of these. The design will allow a definitive answer this important question.

AUTHOR'S CONTRIBUTION

FG, JY, JPD and MW conceived the topic. FG, JY, JPD, LJM and AG designed the study with the input from SK, LS, BN, MW, TM and AW. FG oversaw the running of the trial and all the authors contributed to the ongoing management of the trial. AK, RS, BN collected data for the trial. KT performed the statistical analysis. SF evaluated the qualitative data. The manuscript was drafted by LJM, JY and FG with contributions from the other authors. All the authors contributed to the interpretation of the output and revised and reviewed the paper; they are the guarantors. The Birmingham Clinical Trials Unit undertook the randomisation and data management and monitoring. The authors had full access to all the data from the study. The authors vouch for the accuracy and completeness of the data and analyses. All authors read and approved the final manuscript.

ACKNOWLEDGEMENTS

We thank the trial participants; members of the trial Oversight Committee: Tom Treasure (Chair), Alistair Macfie (independent anaesthetist), Mahmoud Loubani (independent surgeon), Lucy Bradshaw (independent statistician), Alistair Murray (patient and public representative); the Clinical Research Ambassador Group at Heart of England NHS Foundation Trust; Birmingham Clinical Trials Unit staff: Matt Hill (database programmer) and Hannah Watson (data manager).

COMPETING INTERESTS

All authors declare that they have no competing interests.

FUNDING

The study was funded by a grant from the National Institute for Health Research for Patient Benefit (RFPB grant number PB-PG-0213-30126). The funder had no role in study design, data collection, interpretation or analysis, or in writing the report for publication.

For peer review only - http://bmjopen.bmj.com/site/about/guidelines.xhtml

SPONSORSHIP

The study sponsor was the Heart of England NHS Foundation Trust. The sponsor had no role in study design, data collection, interpretation or analysis, or in writing the report for publication.

DATA SHARING STATEMENT

All available study data can be obtained from the corresponding author.

For peer review only

References

1. The Society for Cardiothoracic Surgery in Great Britain and Ireland. SCTS 2014-2015 Thoracic Registry Data. 2015.

2. Hughes R, Gao F. Pain control for thoracotomy. *Continuing Education in Anaesthesia, Critical Care and Pain.* 2005;5(2):56-60.

3. Soto RG, Fu ES. Acute pain management for patients undergoing thoracotomy. *Ann Thorac Surg.* 2003;75(4):1349-1357.

4. Kavanagh BP, Katz J, Sandler AN. Pain control after thoracic surgery. A review of current techniques. *Anesthesiology.* 1994;81(3):737-759.

5. International Association for the Study of Pain. Classification of chronic pain. Descriptions of chronic pain syndromes and definitions of pain terms. Prepared by the International Association for the Study of Pain, Subcommittee on Taxonomy. *Pain Supplement.* 1986;3:S1-226.

6. Bayman EO, Brennan TJ. Incidence and severity of chronic pain at 3 and 6 months after thoracotomy: meta-analysis. *The Journal of Pain.* 2014;15(9):887-897.

7. Dahl JB, Rosenberg J, Lund C, Kehlet H. Effect of thoracic epidural bupivacaine 0.75% on somatosensory evoked potentials after dermatomal stimulation. *Reg Anesth.* 1990;15(2):73-75.

8. Lund C, Hansen OB, Mogensen T, Kehlet H. Effect of thoracic epidural bupivacaine on somatosensory evoked potentials after dermatomal stimulation. *Anesth Analg.*1987;66(8):731-734.

9. Richardson J, Lönnqvist PA. Thoracic paravertebral block. *Br J Anaesth.* 1998;81(2):230-238.

10. Cook E, Downs C. Analgesia After Thoracotomy - The Role of the Extrapleural Paravertebral Catheter. *Australian Anaesthesia.* 2005:103-117.

11. Richardson J, Jones J, Atkinson R. The effect of thoracic paravertebral blockade on intercostal somatosensory evoked potentials. *Anesthesia & Analgesia.* 1998;87(2):373-376.

12. Karmakar MK, Samy W, Li JW, et al. Thoracic paravertebral block and its effects on chronic pain and health-related quality of life after modified radical mastectomy. *Reg Anesth Pain Med.* 2014;39(4):289-298.

13. Turner J, Yeung J. Analgesic technique in thoracic surgery: a survey of UK ACTACC members. Unpublished data.2016.

14. Yeung JH, Gates S, Naidu BV, Wilson MJ, Gao Smith F. Paravertebral block versus thoracic epidural for patients undergoing thoracotomy. *Cochrane Database Syst Rev.* 2016;2:CD009121.

15. Yeung J, Melody T, Kerr A, Naidu B, Middleton L, Tryposkiadis K, Daniels J, Gao F. Randomised controlled pilot study to investigate the effectiveness of thoracic epidural and paravertebral blockade in reducing chronic post-thoracotomy pain: TOPIC feasibility study protocol. BMJ Open. 2016 Dec 1;6(12):e012735. doi: 10.1136/bmjopen-2016-012735.

For peer review only

16. Jensen MP, Karoly P, Braver S. The measurement of clinical pain intensity: a comparison of six methods. Pain. 1986 Oct;27(1):117-26.

17. Tan G, Jensen MP, Thornby JI, et al. Validation of the Brief Pain Inventory for chronic nonmalignant pain. J Pain 2004;5:133–7.

18. Galer BS, Jensen MP. Development and preliminary validation of a pain measure specific to neuropathic pain: the Neuropathic Pain Scale. Neurology 1997;48:332–8.

19. Herdman M, Gudex C, Lloyd A, et al. Development and preliminary testing of the new five-level version of EQ-5D (EQ-5D-5L). Qual Life Res 2011;20:1727–36.

20. Zigmond AS, Snaith RP. The hospital anxiety and depression scale. Acta Psychiatr Scand. 1983;67:361–70.

21. Moore CG, Carter RE, Nietert PJ, Stewart PW. Recommendations for planning pilot studies in clinical and translational research. Clin Transl Sci. 2011 Oct;4(5):332-7. doi: 10.1111/j.1752-8062.2011.00347.x.

22. Jensen MP, Chen C, Brugger AM. Interpretation of visual analog scale ratings and change scores: a reanalysis of two clinical trials of postoperative pain. J Pain. 2003;4:407-14.

23. Palos GR, Mendoza TR, Mobley GM, Cantor SB, Cleeland CS. Asking the community about cutpoints used to describe mild, moderate, and severe pain. J Pain. 2006 Jan;7(1):49-56.

24. Litcher-Kelly L, Martino SA, Broderick JE, Stone AA. A systematic review of measures used to assess chronic musculoskeletal pain in clinical and randomized controlled clinical trials. The journal of pain: official journal of the American Pain Society. 2007;8(12):906-913.doi:10.1016/j.jpain.2007.06.009.

25. Edwards P, Roberts I, Sandercock P, Frost C. Follow-up by mail in clinical trials: does questionnaire length matter? Control Clin Trials. 2004 Feb;25(1):31-52

26. Bendixen M, Jørgensen OD, Kronborg C, Andersen C, Licht PB. Postoperative pain and quality of life after lobectomy via video-assisted thoracoscopic surgery or anterolateral thoracotomy for early stage lung cancer: a randomised controlled trial. Lancet Oncol. 2016 Jun;17(6):836-844. doi: 10.1016/S1470-2045(16)00173-X. Epub 2016 May 6.

27. Ferlay J, Soerjomataram I, Ervik M, Dikshit R, Eser S, Mathers C et al. GLOBOCAN 2012 v1.0, Cancer Incidence and Mortality Worldwide: IARC Cancer Base No. 11.

28. Stewart BW, Wild CP, editors. World cancer report 2014. Lyon: International Agency for Research on Cancer; 2014.

29. Eldridge SM, Chan CL, Campbell MJ, Bond CM, Hopewell S, Thabane L, Lancaster GA. CONSORT 2010 statement: extension to randomised pilot and feasibility trials. BMJ. 2016 Oct 24;355:i5239. doi: 10.1136/bmj.i5239.

For peer review only

30. Thabane L, Ma J, Chu R, Cheng J, Ismaila A, Rios LP, Robson R, Thabane M, Giangregorio L, Goldsmith CH. A tutorial on pilot studies: the what, why and how. BMC Medical Research Methodology201010:1 https://doi.org/10.1186/1471-2288-10-1.

31. Dworkin RH, Turk DC, Farrar JT, Haythornthwaite JA, Jensen MP, Katz NP et al. Core outcome measures for chronic pain clinical trials: IMMPACT recommendations. Pain. 2005; 113:9-19.

For peer review only

LEGEND FOR FIGURE 1

Figure 1. Flow chart of patients through the study

For peer review only

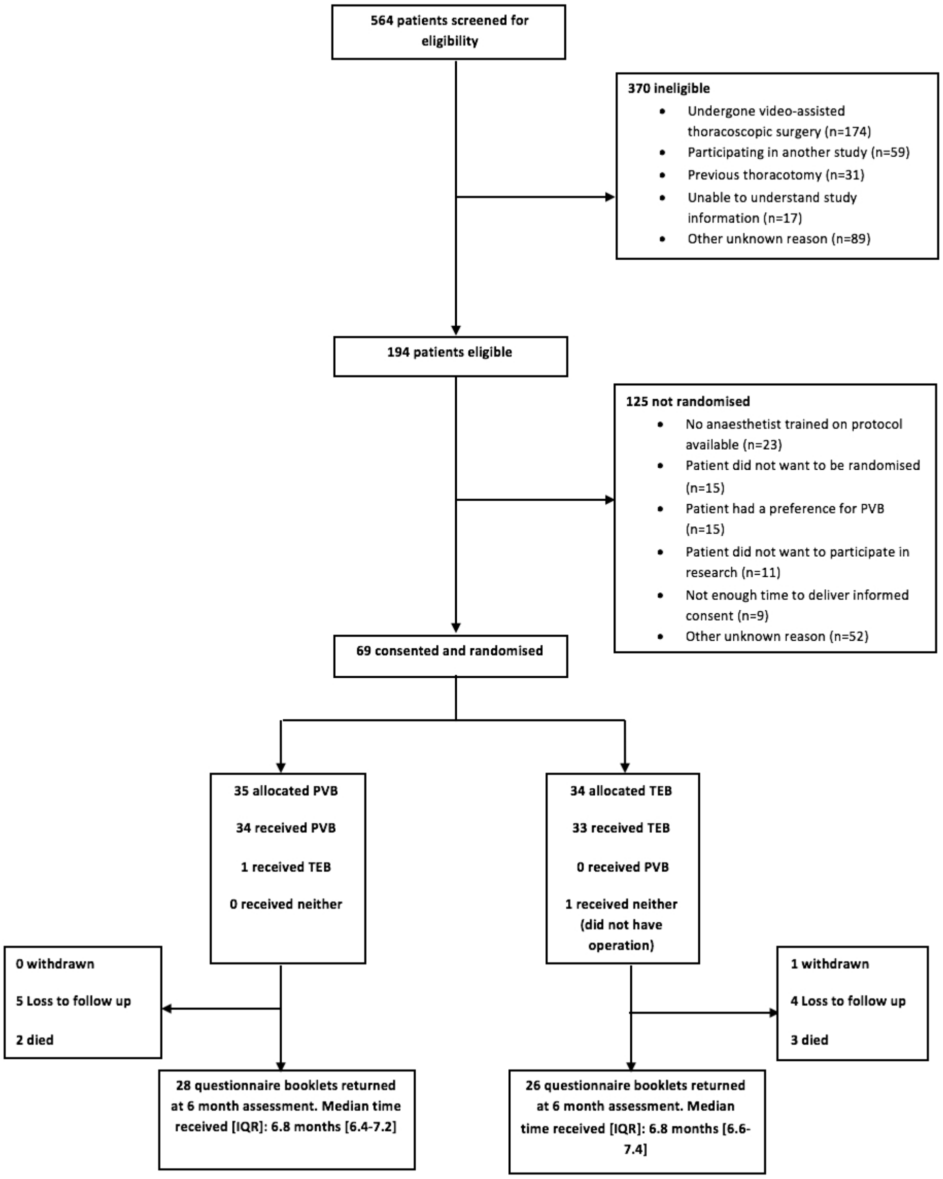

Figure 1

79x98mm (300 x 300 DPI)

TOPIC PILOT STUDY SUPPLEMENTARY APPENDIX

Table S1: Intraoperative data summary 2

Table S2: Analgesic use summary 3

Table S3: Post-operative (in hospital) pain scores summary 4

Table S4: Comprehensive summary of Visual Analogue Scale (VAS) scores 5

Table S5: Complications, short-term mortality and length of stay summary 7

Table S6: Patient satisfaction and blinding questions results summary 8

For peer review only

Table S1: Intraoperative data summary

		PVB (n=35)	TEB (n=33)[1]
Vasopressor/inotrope used		30 (86)	27 (82)
Delivery route	Boluses	24 (80)	24 (89)
	Infusion	3 (10)	1 (4)
	Both	3 (10)	2 (7)
Overall fluid therapy and balance	Median [IQR]	1000 [1000, 2000]	1000 [1000, 1500]
Grade of surgeon was consultant (as opposed to trainee)		21 (60)	18 (55)
Side of operation was left (as opposed to right)		9 (26)	10 (30)
Type of operation	Lobectomy	18 (51)	18 (55)
	Pneumonectomy	3 (9)	2 (6)
	Segmentectomy	1 (3)	1 (3)
	Wedge	3 (9)	2 (6)
	Lung biopsy	1 (3)	0 (-)
	Wedge and Segmentectomy	0 (-)	1 (3)
	Lobectomy and Wedge	1 (3)	0 (-)
	Lobectomy and Other	2 (6)	2 (6)
	Other	6 (17)	7 (21)
Surgical approach	Anterolateral	4 (12)[2]	2 (6)
	Posterolateral	22 (65)[2]	23 (70)
	Muscle sparing	0 (-)[2]	1 (3)
	Mini-thoracotomy	1 (3)[2]	0 (-)
	Posterolateral and Muscle sparing	7 (21)[2]	6 (18)
	Mini-limited lateral	0 (-)[2]	1 (3)

Frequency (%) presented (unless otherwise stated)
[1] one participant received neither technique as they did not have an operation; [2] one response missing (n=34)

For peer review only

Table S2: Analgesic use summary

			In Hospital (post-surgery)							
	Intraoperative		Day 1		Day 2		Day 3		Between day 3 and discharge/final assessment	
**Drug used**	**PVB**	**TEB**	**PVB**	**TEB**	**PVB**	**TEB**	**PVB**	**TEB**	**PVB**	**TEB**
Paracetamol	29	29	1	-	-	-	-	-	-	-
Diclofenac	3	-	3	2	-	-	-	-	-	-
Remifentanil	-	-	6	5	2	-	-	-	-	-
Fentanyl	-	-	16	11	5	4	2	-	-	-
Morphine	2	-	6	1	14	3	3	2	2	-
Diamorphine	-	2	-	10	1	7	-	1	-	-
Gabapentin	-	-	-	-	-	-	-	1	-	-
Alfentanil	-	-	1	2	3	10	1	1	-	-
Bupivicaine	-	-	-	-	-	-	-	1	-	-
Levobupivicaine	-	-	-	-	-	1	-	-	-	-
Pregabalin	-	-	-	-	-	-	1	-	-	-
Ketorolac	-	-	-	-	-	2	3	2	2	-

Number of participants administered drug indicated (total number given PVB=35 and total number given TEB=33)

For peer review only - http://bmjopen.bmj.com/site/about/guidelines.xhtml

Table S3: Post-operative (in hospital) pain scores summary

	PVB mean score (SD, n)	TEB mean score (SD, n)	Difference between groups (95% CI) [1]
Pain score at rest (0-3, higher=worse pain)			
Day 1	0.4 (0.7, 34)	0.5 (1.0, 33)	-0.1 (-0.5, 0.3)
Day 2	0.5 (0.7, 33)	0.7 (1.0, 32)	-0.2 (-0.6, 0.3)
Day 3	0.2 (0.4, 27)	0.6 (0.7, 27)	-0.4 (-0.8, -0.1)
Pain score at movement (0-3, higher=worse pain)			
Day 1	0.9 (0.9, 34)	0.8 (1.0, 33)	0.1 (-0.3, 0.6)
Day 2	1.2 (0.8, 33)	1.3 (0.9, 32)	-0.1 (-0.5, 0.4)
Day 3	0.8 (0.9, 27)	1.0 (1.0, 27)	-0.2 (-0.8, 0.3)

[1] scores<0 indicate less pain with PVB

For peer review only

Table S4: Comprehensive summary of Visual Analogue Scale (VAS) scores

	PVB mean score (SD, n)	TEB mean score (SD, n)	Difference between groups (95% CI) [1]
Average chest pain overall (0-10, higher=worse pain)			
Baseline	0.6 (1.8, 32)	0.7 (1.7, 33)	-1.0 (-1.0, 0.8)
24 hours post-surgery	5.2 (2.5, 33)	3.7 (3.4, 32)	1.5 (-0.02, 2.9)
48 hours post-surgery	6.4 (1.8, 31)	5.5 (2.7, 31)	0.9 (-0.3, 2.1)
72 hours post-surgery	5.1 (25, 2.4)	6.1 (2.5, 26)	-1.0 (-2.4, 0.3)
Discharge	4.9 (1.9, 29)	4.3 (2.5, 32)	0.6 (-0.5, 1.7)
3 months	2.1 (1.8, 25)	2.0 (2.6, 21)	0.1 (-1.3, 1.4)
**6 months**	0.8 (1.2, 27)	1.7 (2.1, 25)	-0.9 (-1.8, 0.1)
Average chest pain at rest (0-10, higher=worse pain)			
Baseline	0.4 (1.4, 32)	0.7 (1.7, 33)	-0.3 (-0.9, 0.5)
24 hours post-surgery	3.9 (2.2, 33)	3.3 (3.3, 32)	0.6 (-0.8, 2.0)
48 hours post-surgery	3.9 (2.1, 31)	4.3 (2.8, 31)	-0.4 (-1.6, 0.9)
72 hours post-surgery	3.2 (25, 2.6)	4.8 (2.8, 25)	-1.6 (-3.2, -0.1)
Discharge	3.1 (2.2, 29)	3.2 (2.5, 32)	-0.1 (-1.3, 1.1)
3 months	1.3 (1.4, 25)	1.5 (2.2, 21)	-0.2 (-1.3, 0.9)
**6 months**	0.4 (0.7, 27)	1.2 (1.7, 25)	-0.8 (-1.6, -0.1)
Average chest pain after coughing (0-10, higher=worse pain)			
Baseline	0.7 (1.9, 32)	0.9 (1.9, 33)	-0.2 (-1.2, 0.7)
24 hours post-surgery	6.2 (2.4, 33)	4.7 (3.3, 32)	1.5 (0.04, 2.9)
48 hours post-surgery	6.4 (2.5, 31)	6.1 (2.6, 31)	0.3 (-1.0, 1.5)
72 hours post-surgery	6.3 (2.6, 25)	7.0 (2.5, 26)	-0.7 (-2.2, 0.7)
Discharge	5.8 (2.2, 29)	5.5 (2.8, 32)	0.4 (-0.9, 1.7)
3 months	2.0 (1.8, 25)	1.95 (2.7, 21)	0.05 (-1.4, 1.4)
**6 months**	1.5 (1.9, 27)	2.1 (2.5, 24)	-0.6 (-1.8, 0.7)
Average chest pain after/with moving (0-10, higher=worse pain)			
Baseline	0.6 (1.9, 32)	0.8 (1.8, 33)	-0.2 (-1.1, 0.7)
24 hours post-surgery	6.1 (2.4, 33)	4.2 (3.3, 32)	1.9 (0.4, 3.3)
48 hours post-surgery	5.9 (2.3, 32)	5.4 (3.0, 31)	0.5 (-0.8, 1.9)
72 hours post-surgery	5.0 (2.6, 24)	6.3 (2.6, 25)	-1.3 (-2.8, 0.2)
Discharge	4.9 (2.2, 31)	4.5 (2.7, 31)	0.4 (-0.9, 1.6)
3 months	1.9 (1.8, 24)	2.2 (3.0, 23)	-0.3 (-1.8, 1.1)
**6 months**	1.0 (1.6, 27)	2.0 (2.7, 26)	-1.0 (-2.2, 0.3)
Average chest pain after physiotherapy (0-10, higher=worse pain)			
Baseline	0.04 (0.1, 29)	0.2 (0.5, 28)	-0.16 (-1.1, 0.7)
24 hours post-surgery	5.4 (2.3, 31)	4.1 (3.2, 30)	1.3 (-0.2, 2.7)
48 hours post-surgery	5.6 (2.3, 32)	5.0 (2.8, 31)	0.6 (-0.8, 1.9)
72 hours post-surgery	4.9 (2.4, 24)	5.9 (2.8, 25)	-1.0 (-2.5, 0.5)
Discharge	4.8 (2.1, 30)	3.8 (2.6 ,30)	1.0 (-0.2, 2.3)
3 months	1.95 (2.0, 18)	2.0 (2.9, 19)	-0.05 (-1.7, 1.6)
**6 months**	1.1 (1.6, 21)	2.0 (2.9, 19)	-0.9 (-2.3, 0.7)
Worst chest pain overall (0-10, higher=worse pain)			
Baseline	0.7 (1.7, 33)	0.9 (2.1, 33)	-0.2 (-1.2, 0.7)
24 hours post-surgery	7.7 (2.0, 33)	6.0 (3.5, 32)	1.7 (0.3, 3.2)
48 hours post-surgery	8.2 (1.6, 32)	7.4 (2.5, 31)	0.8 (-0.3, 1.9)
72 hours post-surgery	7.5 (2.0, 24)	7.9 (2.1, 25)	-0.4 (-1.6, 0.7)
Discharge	7.2 (2.1, 31)	6.6 (2.9, 31)	0.6 (-0.7, 1.9)
3 months	3.5 (2.9, 24)	2.7 (3.4, 23)	0.8 (-1.1, 2.7)
**6 months**	1.9 (2.5, 27)	3.1 (3.4, 26)	-1.2 (-2.8, 0.5)
Worst chest pain at rest (0-10, higher=worse pain)			
Baseline	0.3 (1.2, 33)	0.7 (1.7, 33)	-0.4 (-1.1, 0.4)
24 hours post-surgery	5.7 (2.8, 32)	4.8 (3.6, 32)	0.9 (-0.7, 2.5)
48 hours post-surgery	5.5 (2.7, 32)	5.4 (3.1, 31)	0.1 (-1.4, 1.6)
72 hours post-surgery	4.6 (2.8, 24)	6.1 (3.0, 25)	-1.5 (-3.2, 0.1)
Discharge	4.3 (2.5, 31)	4.7 (2.7, 31)	-0.4 (-1.7, 0.9)
3 months	2.2 (2.3, 24)	1.9 (2.7, 23)	0.3 (-1.2, 1.7)

For peer review only

**6 months**	1.0 (1.6, 27)	1.9 (2.7, 26)	-0.9 (-2.1, 0.3)
Worst chest pain after coughing (0-10, higher=worse pain)			
Baseline	0.6 (1.6, 33)	1.0 (2.2, 33)	-0.4 (-1.4, 0.6)
24 hours post-surgery	7.2 (1.9, 33)	5.5 (3.4, 32)	1.7 (0.3, 3.1)
48 hours post-surgery	7.6 (2.4, 32)	7.0 (2.5, 31)	0.6 (-0.7, 1.7)
72 hours post-surgery	7.2 (2.5, 25)	7.8 (2.1, 26)	-0.6 (-1.9, 0.7)
Discharge	6.7 (2.3, 30)	5.9 (2.9, 32)	0.8 (-0.5, 2.1)
3 months	2.9 (2.5, 25)	2.8 (3.1, 22)	0.1 (-1.7, 1.7)
**6 months**	1.8 (2.2, 28)	2.5 (2.9, 26)	-0.6 (-2.1, 0.8)
Worst chest pain after/with moving (0-10, higher=worse pain)			
Baseline	0.5 (1.5, 33)	0.9 (1.9, 33)	-0.4 (-1.2, 0.5)
24 hours post-surgery	7.2 (2.1, 33)	5.4 (3.6, 32)	1.8 (0.3, 3.3)
48 hours post-surgery	7.3 (1.9, 32)	6.5 (2.6, 31)	0.8 (-0.3, 2.1)
72 hours post-surgery	6.3 (2.3, 25)	7.0 (2.6, 26)	-0.7 (-2.1, 0.7)
Discharge	6.4 (2.3, 30)	5.8 (2.7, 32)	0.6 (-0.6, 1.9)
3 months	2.3 (1.8, 25)	2.5 (2.8, 22)	-0.2 (-1.6, 1.2)
**6 months**	1.3 (1.8, 28)	2.5 (3.2, 26)	-1.1 (-2.6, 0.4)
Worst chest pain after physiotherapy (0-10, higher=worse pain)			
Baseline	0.1 (0.2, 30)	0.2 (0.6, 29)	-0.1 (-0.4, 0.1)
24 hours post-surgery	6.2 (2.3, 31)	5.0 (3.5, 30)	1.2 (-0.4, 2.7)
48 hours post-surgery	6.5 (2.6, 31)	6.0 (2.8, 31)	0.6 (-0.8, 1.9)
72 hours post-surgery	5.9 (2.6, 25)	6.5 (2.6, 26)	-0.6 (-2.1, 0.8)
Discharge	6.0 (2.3, 29)	4.8 (2.8, 31)	1.2 (-0.1, 2.5)
3 months	2.6 (1.8, 25)	1.9 (2.7, 17)	0.7 (-0.9, 2.3)
**6 months**	1.2 (1.7, 22)	1.8 (2.8, 18)	-0.6 (-2.2, 1.0)

[1] scores<0 indicate less pain with PVB

For peer review only

Table S5: Complications, short-term mortality and length of stay summary

	Day 1		Day 2		Day 3		Between day 3 and discharge/final assessment	
	PVB	TEB	PVB	TEB	PVB	TEB	PVB	TEB
Arrhythmias	1/34 (3)	2/33 (6)	2/32 (6)	4/32 (13)	1/26 (4)	1/27 (4)	1/34 (3)	3/32 (9)
Systolic blood pressure (<90mmHg)	2/35 (6)	8/33 (24)	2/33 (6)	2/32 (6)	2/27 (7)	2/27 (7)	0/34 (-)	0/32 (-)
Low Respiratory rate (<10/minute)	1/35 (3)	0/33 (-)	0/33 (-)	0/32 (-)	0/27 (-)	0/27 (-)	0/34 (-)	0/32 (-)
Drowsiness	6/34 (18)	4/33 (12)	3/32 (9)	2/32 (6)	0/26 (-)	2/27 (7)	0/34 (-)	1/32 (3)
Nausea & vomiting	8/35 (23)	11/33 (33)	4/33 (12)	4/32 (13)	2/26 (8)	3/27 (11)	5/34 (15)	5/32 (16)
Urinary retention	0/35 (-)	0/33 (-)	1/33 (3)	0/32 (-)	0/27 (-)	0/27 (-)	1/34 (3)	1/32 (3)
Itching	0/34	1/33 (3)	0/32	1/32 (3)	0/26	0/27	0/32	1/32 (3)
High block (above T4)	0/35 (-)	0/33 (-)	0/33 (-)	0/32 (-)	0/27 (-)	0/27 (-)	0/34 (-)	0/32 (-)
Post-dural puncture headache	0/35 (-)	0/33 (-)	0/33 (-)	0/32 (-)	0/27 (-)	0/27 (-)	1/34 (3)	0/32 (-)
Post-op pulmonary complications (PPC)	2/21 (10)	1/19 (5)	2/19 (10)	1/18 (6)	1/15 (7)	1/15 (7)	0/19 (-)	0/19 (-)
Other	20/34 (59)	13/33 (39)	6/32 (19)	7/31 (23)	4/26 (15)	6/27 (22)	3/34 (9)	9/32 (28)
**Level 2 admission**	0/35 (-)	2/33 (6)	-	-	-	-	0/34 (-)	1/32 (3)
If yes, cardiovascular support	-	2/2 (100)	-	-	-	-	-	-
If yes, respiratory support	-	2/2 (100)	-	-	-	-	-	-
If yes, organ support	-	-	-	-	-	-	-	1/1 (100)
If yes, total length of stay *(Median [IQR], n)*		9 [6, 12]						6 [-]
**Level 3 admission**	1/35 (3)	1/32 (3)						
If yes, cardiovascular support	1/1 (100)	1/1 (100)	-	-	-	-	-	-
If yes, respiratory support	1/1 (100)	1/1 (100)	-	-	-	-	-	-
If yes, renal support	0/1 (-)	0/1 (-)	-	-	-	-	-	-
If yes, total length of stay *(Median [IQR], n)*	1 [-]	5 [-]	-	-	-	-	-	-
Discharge with flutter bag	-	-	-	-	-	-	5/34 (15)	4/33 (12)
Mortality	-	-	-	-	-	-	1/35 (3)	1/33 (3)

Frequency (%) presented (unless otherwise stated)

For peer review only - http://bmjopen.bmj.com/site/about/guidelines.xhtml

Table S6: Patient satisfaction and blinding questions results summary

		PVB	TEB
Satisfaction with pain therapy after surgery			
Discharge (n=31 for PVB, n=32 for TEB)	Very satisfied	18 (58)	20 (63)
	Satisfied	10 (33)	9 (28)
	Dissatisfied	2 (6)	2 (6)
	Very dissatisfied	1 (3)	1 (3)
3 months (n=26 for PVB, n=23 for TEB)	Very satisfied	13 (50)	9 (39)
	Satisfied	13 (50)	8 (35)
	Dissatisfied	0 (-)	6 (26)
	Very dissatisfied	0 (-)	0 (-)
6 months (n=26 for PVB, n=25 for TEB)	Very satisfied	14 (54)	16 (64)
	Satisfied	11 (42)	6 (24)
	Dissatisfied	0 (-)	2 (8)
	Very dissatisfied	1 (4)	1 (4)
Satisfaction with care provided by hospital			
Discharge (n=31 for PVB, n=32 for TEB)	Very satisfied	22 (71)	22 (69)
	Satisfied	9 (29)	8 (25)
	Dissatisfied	0 (-)	2 (6)
	Very dissatisfied	0 (-)	0 (-)
3 months (n=26 for PVB, n=23 for TEB)	Very satisfied	15 (58)	12 (52)
	Satisfied	11 (42)	9 (39)
	Dissatisfied	0 (-)	2 (9)
	Very dissatisfied	0 (-)	0 (-)
6 months (n=26 for PVB, n=26 for TEB)	Very satisfied	19 (73)	15 (58)
	Satisfied	5 (19)	9 (35)
	Dissatisfied	2 (8)	2 (8)
	Very dissatisfied	0 (-)	0 (-)
Do you know which technique you received?			
Discharge (n=31 for PVB, n=32 for TEB)	TEB	1 (3)	19 (60)
	PVB	8 (26)	3 (9)
	Don't know	22 (71)	10 (31)
3 months (n=26 for PVB, n=22 for TEB)	TEB	2 (8)	12 (55)
	PVB	2 (8)	0 (-)
	Don't know	22 (84)	10 (45)
6 months (n=26 for PVB, n=25 for TEB)	TEB	2 (8)	13 (52)
	PVB	5 (19)	1 (4)
	Don't know	19 (73)	11 (44)

Frequency (%) presented (unless otherwise stated)

For peer review only

CONSORT STATEMENT – PILOT STUDY

Section/topic and item No	Standard checklist item	Extension for pilot trials	Page No where item is reported
Title and abstract			
1a	Identification as a randomised trial in the title	Identification as a pilot or feasibility randomised trial in the title	1
1b	Structured summary of trial design, methods, results, and conclusions (for specific guidance see CONSORT for abstracts)	Structured summary of pilot trial design, methods, results, and conclusions (for specific guidance see CONSORT abstract extension for pilot trials)	2
Introduction			
Background and objectives:			
2a	Scientific background and explanation of rationale	Scientific background and explanation of rationale for future definitive trial, and reasons for randomised pilot trial	4
2b	Specific objectives or hypotheses	Specific objectives or research questions for pilot trial	4
Methods			
Trial design:			
3a	Description of trial design (such as parallel, factorial) including allocation ratio	Description of pilot trial design (such as parallel, factorial) including allocation ratio	4
3b	Important changes to methods after trial commencement (such as eligibility criteria), with reasons	Important changes to methods after pilot trial commencement (such as eligibility criteria), with reasons	N/A

For peer review only

Participants:			
4a	Eligibility criteria for participants		5
4b	Settings and locations where the data were collected		4
4c		How participants were identified and consented	7
Interventions:			
5	The interventions for each group with sufficient details to allow replication, including how and when they were actually administered		5-6
Outcomes:			
6a	Completely defined prespecified primary and secondary outcome measures, including how and when they were assessed	Completely defined prespecified assessments or measurements to address each pilot trial objective specified in 2b, including how and when they were assessed	6
6b	Any changes to trial outcomes after the trial commenced, with reasons	Any changes to pilot trial assessments or measurements after the pilot trial commenced, with reasons	N/A
6c		If applicable, prespecified criteria used to judge whether, or how, to proceed with future definitive trial	6
Sample size:			
7a	How sample size was determined	Rationale for numbers in the pilot trial	6
7b	When applicable, explanation of any interim analyses and stopping guidelines		N/A
Randomisation:			
Sequence generation:			

For peer review only

8a	Method used to generate the random allocation sequence		5
8b	Type of randomisation; details of any restriction (such as blocking and block size)	Type of randomisation(s); details of any restriction (such as blocking and block size)	5
Allocation concealment mechanism:			
9	Mechanism used to implement the random allocation sequence (such as sequentially numbered containers), describing any steps taken to conceal the sequence until interventions were assigned		5
Implementation:			
10	Who generated the random allocation sequence, enrolled participants, and assigned participants to interventions		5
Blinding:			
11a	If done, who was blinded after assignment to interventions (eg, participants, care providers, those assessing outcomes) and how		6
11b	If relevant, description of the similarity of interventions		6
Analytical methods:			
12a	Statistical methods used to compare	Methods used to address each pilot trial objective whether	7

For peer review only

	groups for primary and secondary outcomes	qualitative or quantitative	
12b	Methods for additional analyses, such as subgroup analyses and adjusted analyses	Not applicable	N/A
Results			
Participant flow (a diagram is strongly recommended):			
13a	For each group, the numbers of participants who were randomly assigned, received intended treatment, and were analysed for the primary outcome	For each group, the numbers of participants who were approached and/or assessed for eligibility, randomly assigned, received intended treatment, and were assessed for each objective	Figure 1
13b	For each group, losses and exclusions after randomisation, together with reasons		Figure 1
Recruitment:			
14a	Dates defining the periods of recruitment and follow-up		8
14b	Why the trial ended or was stopped	Why the pilot trial ended or was stopped	N/A
Baseline data:			
15	A table showing baseline demographic and clinical characteristics for each group		Table 1
Numbers analysed:			

For peer review only

16	For each group, number of participants (denominator) included in each analysis and whether the analysis was by original assigned groups	For each objective, number of participants (denominator) included in each analysis. If relevant, these numbers should be by randomised group	Table 2
Outcomes and estimation:			
17a	For each primary and secondary outcome, results for each group, and the estimated effect size and its precision (such as 95% confidence interval)	For each objective, results including expressions of uncertainty (such as 95% confidence interval) for any estimates. If relevant, these results should be by randomised group	Table 3, 4
17b	For binary outcomes, presentation of both absolute and relative effect sizes is recommended	Not applicable	N/A
Ancillary analyses:			
18	Results of any other analyses performed, including subgroup analyses and adjusted analyses, distinguishing prespecified from exploratory	Results of any other analyses performed that could be used to inform the future definitive trial	N/A
Harms:			
19	All important harms or unintended effects in each group (for specific guidance see CONSORT for harms)		14
19a		If relevant, other important unintended consequences	N/A
Discussion			
Limitations:			

For peer review only

20	Trial limitations, addressing sources of potential bias, imprecision, and, if relevant, multiplicity of analyses	Pilot trial limitations, addressing sources of potential bias and remaining uncertainty about feasibility	14-17
Generalisability:			
21	Generalisability (external validity, applicability) of the trial findings	Generalisability (applicability) of pilot trial methods and findings to future definitive trial and other studies	14-17
Interpretation:			
22	Interpretation consistent with results, balancing benefits and harms, and considering other relevant evidence	Interpretation consistent with pilot trial objectives and findings, balancing potential benefits and harms, and considering other relevant evidence	14-15
22a		Implications for progression from pilot to future definitive trial, including any proposed amendments	15-17
Other information			
Registration:			
23	Registration number and name of trial registry	Registration number for pilot trial and name of trial registry	2
Protocol:			
24	Where the full trial protocol can be accessed, if available	Where the pilot trial protocol can be accessed, if available	N/A
Funding:			
25	Sources of funding and other support (such as supply of drugs), role of funders		17
26		Ethical approval or approval by research review committee, confirmed with reference number	5

For peer review only

A RANDOMISED CONTROLLED TRIAL TO INVESTIGATE THE EFFECTIVENESS OF THORACIC EPIDURAL AND PARAVERTEBRAL BLOCKADE IN REDUCING CHRONIC POST-THORACOTOMY PAIN (TOPIC) – A PILOT STUDY TO ASSESS FEASIBILITY OF A LARGE MULTI-CENTRE TRIAL

Journal:	*BMJ Open*
Manuscript ID	bmjopen-2018-023679.R2
Article Type:	Research
Date Submitted by the Author:	16-Oct-2018
Complete List of Authors:	Yeung, Joyce ; University of Warwick, Warwick Medical School; Heart of England NHS Foundation Trust, Department of Anaesthesia<br>Middleton, Lee; University of Birmingham, School of Health and Population Sciences<br>Tryposkiadis, Kostas; University of Birmingham, Birmingham Clinical Trials Unit<br>Kerr, Amy; Heart of England NHS Foundation Trust, Department of Thoracic Surgery<br>Daniels, Jane; University of Nottingham, Nottingham Clinical Trials Unit<br>Naidu, Babu; University of Birmingham, Institute of Inflammation and Ageing; Heart of England NHS Foundation Trust, Department of Thoracic Surgery<br>Melody, Teresa; Heart of England NHS Foundation Trust, Academic Department of Anaesthesia, Critical Care, Pain and Resuscitation<br>Goebel, Andreas; University of Liverpool, Institute of Translational Medicine<br>Wilson, Matthew; University of Sheffield, Health Services Research<br>Kumar, Sajith; Heart of England NHS Foundation Trust, Department of Anaesthesia<br>Szentgyorgyi, Lajos; University Hospital of South Manchester NHS Foundation Trust, Department of Anaesthesia<br>Flanagan, Sarah; University of Birmingham, Institute of Applied Health Research<br>Shah, Rajesh; University Hospital of South Manchester NHS Foundation Trust, Department of Thoracic Surgery<br>Worrall, Antony; Heart of England NHS Foundation Trust<br>Gao, Fang; University of Birmingham, Institute of Inflammation and Ageing, College of Medical and Dental Sciences; Heart of England NHS Foundation Trust, Academic Department of Anaesthesia, Critical Care, Pain and Resuscitation
**Primary Subject Heading**:	Anaesthesia
Secondary Subject Heading:	Surgery
Keywords:	Pain management < ANAESTHETICS, Adult anaesthesia <

	ANAESTHETICS, Thoracic surgery < SURGERY, Clinical trials < THERAPEUTICS

SCHOLARONE™ Manuscripts

A RANDOMISED CONTROLLED TRIAL TO INVESTIGATE THE EFFECTIVENESS OF THORACIC EPIDURAL AND PARAVERTEBRAL BLOCKADE IN REDUCING CHRONIC POST-THORACOTOMY PAIN (TOPIC) – A PILOT STUDY TO ASSESS FEASIBILITY OF A LARGE MULTI-CENTRE TRIAL

Authors:

J Yeung[1,2], L Middleton[3], K Tryposkiadis[3], A Kerr[1], J Daniels[4], B Naidu[1,5], T Melody[1], A Goebel[6], M Wilson[7], S Kumar[1], L Szentgyorgyi[8], S Flanagan[9], R Shah[8], A Worrall[1], and F Gao[1,5], On behalf of TOPIC Investigator Group

[1] Department of Anaesthesia, Heart of England NHS Foundation Trust, Birmingham, United Kingdom

[2] Warwick Medical School, University of Warwick, Coventry, United Kingdom

[3] Birmingham Clinical Trials Unit, University of Birmingham, Birmingham, United Kingdom

[4]Nottingham Clinical Trials Unit, University of Nottingham, Nottingham, United Kingdom

[5]Institute of Inflammation and Ageing, University of Birmingham, Birmingham, United Kingdom

[6]University of Liverpool and Walton Centre NHS Foundation Trust, Liverpool, United Kingdom

[7]School of Health and Related Research, University of Sheffield, Sheffield, United Kingdom

[8]Manchester University NHS Foundation Trust, Manchester, United Kingdom

[9]Institute of Applied Health Research, University of Birmingham, Birmingham, United Kingdom

Corresponding author: Fang Gao

E-mail: f.gaosmith@bham.ac.uk Tel: 01214147483

Institute of Inflammation and Ageing, College of Medical and Dental Sciences, University of Birmingham, Birmingham B15 2TT, United Kingdom

Word Count 3618

For peer review only

ABSTRACT

Objectives: Thoracotomy is considered one of the most painful surgical procedures. The incidence of chronic post-thoracotomy pain (CPTP) is up to 50%. Paravertebral blockade (PVB) may be superior to thoracic epidural blockade (TEB) in preventing CPTP. The specific objective of this pilot study was to assess the feasibility of conducting a larger trial to determine whether PVB at thoracotomy is more effective in reducing CPTP compared to TEB.

Design: A randomised, parallel, external pilot study was conducted to assess whether a large randomised trial of TEB and PVB with CPTP as the primary outcome is feasible.

Setting: Two adult thoracic centres in the UK

Participants: All adult patients admitted for elective open thoracotomy. Participants were excluded if they were ASA physical status IV or V; or if there is contraindication to local anaesthetics; infection near the proposed puncture site; coagulation/thoracic spine disorders; required chest wall resection or emergency thoracic surgery or had a previous thoracotomy.

Results: All patients presenting for thoracotomy were screened over a 12-month period with 194 found to be eligible. Of these, 69 (36%) were randomised (95%CI: 29%-42%). Discounting five participants who died, 54 of 64 participants (84%) returned questionnaire booklets at six months. The number of participants indicating at least a moderate level of chest pain at six months was lower with PVB but with high levels of uncertainty (RR: 0.7; 95%CI: 0.3-1.7 for worst pain; RR: 0.3; 95%CI: 0.0-2.8 for average). There were no safety concerns.

Conclusions: A large multicentre RCT of PVB versus TEB is feasible as it is possible to randomise and follow up participants with high fidelity. Pain scores were lower on average with PVB compared to TEB but a much larger trial is required to confirm this reliably.

Trial Registration: ISRCTN45041624

For peer review only

Strengths and limitations of this study

- This randomised, external, pilot trial had pre-planned feasibility thresholds to assess whether a large trial is feasible.
- Selected two sites represented the national thoracic and anaesthetic cohort, providing best representation of full trial national recruitment
- Consultant anaesthetists in both participating sites had adequate training and appropriate assessment for the competence of each technique.
- The study was not designed and powered to be large or long enough to provide convincing evidence to support the use of either TEB or PVB as intraoperative anaesthetic technique, but provides evidence that a larger, substantive trial is feasible.

For peer review only

BACKGROUND

An estimated 7200 thoracotomies (surgical incision into the chest wall) are performed annually in the UK, most commonly to treat lung cancer.[1] Thoracotomy is considered one of the most painful surgical procedures due to tissue, muscle and nerve damage from the incision and wound retraction. Intercostal nerve injury can result in a high risk of persistent pain for months after surgery.[2-4] The incidence of chronic post-thoracotomy pain (CPTP; defined as pain that recurs or persists at least two months following the surgery)[5] is thought to be as high as 50%.[6]

Two main analgesic techniques are commonly used for perioperative pain control during thoracotomy. Thoracic epidural blockade (TEB) blocks nerves that supply the chest with local anaesthetic bilaterally, at spinal cord level. It acts by reducing onward transmission of painful nerve signals but may not abolish them completely.[7,8] Paravertebral blockade (PVB), involves injecting local anaesthetic into the paravertebral space, which contains spinal nerves (and sometimes even extension of the dura), white and grey rami communicantes, the sympathetic chain and intercostal vessels, on the side of surgery.[9] There are studies which describe the spread of PVB injections anteriorly across the heads and necks of the ribs to the spaces above and below; medially through an intervertebral foramen or spread laterally in the intercostal plane.[9] However compared to TEB, PVB has the potential to completely block painful nerve signals from reaching the spinal cord.[9,10] This total blockade of nerve signals could remove the stimulus for 'central sensitisation' which underpins the formation of chronic pain pathways. PVB could be uniquely effective in preventing long-term pain[11], and there is evidence from a recent trial of two techniques in breast surgery to support this premise.[12] With limited current evidence to support the most effective choice of anaesthetic technique in preventing CPTP, current UK practice varies greatly.[13] A recent Cochrane Review recommended that a high quality randomised controlled trial (RCT) to compare TEB and PVB with the primary outcome of chronic pain is urgently needed.[14]

The overall aim of this research is to determine whether PVB at thoracotomy is more effective in reducing CPTP compared to TEB. To answer this question a large, multi-centre RCT is required. The specific aim of this pilot study was to assess the feasibility of conducting a larger trial and to enhance the likelihood of its success by developing the necessary structure and processes that a large trial would need. Objectives included: an assessment of the effectiveness of the patient identification and screening process; identification of reasons for failure to randomise; development of educational and training materials for

For peer review only

surgeons and anaesthetists; evaluation of robustness of in-hospital data collection processes; assessment of trial processes including impact on participants and staff.

METHODS

We conducted a randomised, parallel group, external pilot trial of TEB versus PVB for perioperative pain control during thoracotomy. Recruitment took place over 12 months in two adult thoracic centres: Heartlands Hospital, Birmingham and University Hospital South Manchester (Wythenshawe). This study was approved by NHS Research Ethics Committee (NRES Committee East Midlands - Derby, REC number 14/EM/1280) and was prospectively registered (ISRCTN45041624).

Population and inclusion/exclusion criteria

Eligible participants were all adults admitted for an elective open thoracotomy. Participants were excluded if they had any of the following: ASA (classification of fitness) physical status IV or V (as these patients are unlikely to present for elective surgery due to their physical condition); contraindication to local anaesthetics; infection near the proposed puncture site; coagulation abnormalities (in accordance with Association of Anaesthetists in Great Britain and Ireland guidance 2013) ; thoracic spine disorders; required chest wall resection or emergency thoracic surgery or had a previous thoracotomy (scarring due to previous surgery can limit the effectiveness of paravertebral block and these patients may already have existing chronic pain).

Study conduct

All patients thought to fulfil the eligibility criteria were approached with study information at their pre-operative assessment clinic. They were provided with a Patient Information Sheet and given the opportunity to consider participation. Once eligibility was confirmed and written informed consent was obtained, randomisation was performed prior to surgery using a web-based central randomisation system (via Birmingham Clinical Trials Unit) to allocate patients to either TEB or PVB in a 1:1 ratio. Minimisation was used to achieve balance between sex, age (<65 years or ≥65 years), thoracotomy for lung cancer resection or for other indication and study site. Research nurses at each site was responsible for randomisation and assigned group allocation was revealed to responsible anaesthetist when patients arrived at the operating theatre. An Oversight Committee was formed to provide independent guidance to the Trial Management Committee and to review accruing safety information during the period of recruitment. Public and patient involvement was integral to the study throughout.

Interventions

For peer review only

Interventions were delivered by experienced thoracic anaesthetists, trained and deemed competent in both anaesthetic techniques. Participants were not told specifically about their allocated intervention. It was not possible to blind anaesthetists, surgeons or nursing staff due to the nature of the interventions. PVB was performed using three single injections pre-incision with the patient awake or asleep, at the levels of T3-4, 5-6 and 7-8 with 15ml 0.25% levobupivicaine/bupivacaine, using a landmark technique at each level. The PVB catheter was then placed at T5 under direct vision by a surgeon during surgery. A loading dose of 10ml 0.25% levobupivicaine/bupivacaine was administered before chest closure, followed by infusion of 0.125% levobupivicaine/bupivacaine 0.1-0.25ml kg^{-1} hr^{-1} until operation end. TEB was inserted pre-incision with the patient awake or asleep, with a catheter inserted at the spinal level supplying the skin at the incision site (normally T5-6), a test dose of 3ml of 0.5% bupivacaine, and a loading dose of 0.25% levobupivicaine/bupivacaine 0.1ml kg^{-1} with up to 3mg of diamorphine. This was followed by infusion of 0.125% levobupivicaine/bupivacaine with 2µg ml^{-1} fentanyl at 0.1-0.25 ml kg^{-1} hr^{-1}. Further information on delivery and training on the interventions is available in the study protocol.[15] Identical dressings and pumps were used for both interventions to maintain group allocation.

Pilot outcomes

The following outcomes and targets were set a-priori as being indicative that a larger trial would be feasible to conduct.[15] These were: i) patient recruitment rate (the proportion of eligible patients randomised) at least 25%; ii) screening rate (the proportion of all patients listed for elective thoracotomy that were screened for eligibility and recorded on a screening log) at least 90%; iii) clinicians' willingness to recruit: national survey of consultant thoracic anaesthetist indicating the number willing to participate in a larger study at least 70%; iv) data completion – number of anaesthetic/peri-operative forms completed at least 90% and follow-up questionnaires completed at six months at least 80%. The study was given a 12-month recruitment period, after which screening would stop.

Clinical and participant reported outcome measures

Baseline data (including participant completed questions as indicated below) were recorded at the pre-operative assessment appointment. Intraoperative (anaesthetic technique and use), post-operative and discharge data (analgesic use, pain scores on days 1-3, acute complications, mortality and length of stay) were collected by the study team. Three and six-month post-randomisation follow-up was conducted by postal questionnaire. This included: Visual Analogue Scales (VAS)[16] for worst and average chest pain (overall, at rest, after coughing, after moving, after physiotherapy; with higher score - maximum 10 - indicating higher levels of pain, as marked along a 10cm line), Brief Pain Inventory (BPI)[17], Neuropathic

For peer review only

Pain Scale (NPS)[18], generic health-related quality of life (EQ-5D-5L)[19], Hospital Anxiety and Depression Scale (HADS)[20] and assessment of satisfaction and participant blinding. Serious adverse events were collected throughout. Patient outcome data was collected by research team member who was blinded to the assigned group.

Statistics

We expected to recruit between 50-75 participants over a 12-month recruitment period, depending on the number found to be eligible. We estimated that there would be approximately 500 open elective thoracotomies from the two sites, of which 60% would potentially be eligible (300). Using our own target criteria of 25% recruited would yield 75 participants. This number would be sufficient to measure the recruitment rate to uncertainty width up to approximately 10%.

Feasibility outcomes were considered with simple summary statistics (proportions and percentages), with uncertainty estimates provided by 95% confidence intervals. Clinical and participant reported outcome measures were analysed with point estimates (RR or mean difference) and 95% confidence intervals, adjusting for the minimisation variables. They were not subject to hypothesis testing as the size of the sample would not allow reliable assessment of efficacy.[21] Participants were considered in the groups they were randomised to regardless of compliance (intention-to-treat).

Qualitative assessment and survey of practice

In addition to the clinical assessment, semi-structured qualitative interviews were undertaken with 18 patients at 6-8 weeks discharge to gauge the impact of the trial on their hospital care and eight clinical staff (four anaesthetists, one surgeon and three members of the research team) to aid insight into trial related processes. The patient and staff telephone interviews were audio-recorded and field notes were be taken to in order to capture the interviewer's thoughts subsequent to completing each interview. Audio-recordings were transcribed in full by an experienced team of professional transcribers, with transcripts subsequently proof read against the recordings by the researcher. Anonymised transcripts were analysed thematically concurrently with data collection in order to allow emerging findings to be included in subsequent interviews.[22] The resulting codes and themes were refined and consistency and variation across the interviews was explored. Once this process was completed, the resulting themes generated from the data were summarised. An electronic national survey of anaesthetists on willingness to participate in a future trial was also conducted via the Association for Cardiothoracic Anaesthesia and Critical Care membership in 26 UK thoracic units.

For peer review only

Patient involvement

Our study question in chronic pain post thoracotomy is in the top ranked research priorities voted by patient 'What can we do to stop patients developing chronic pain after surgery?' The development of study protocol was designed in conjunction with our patient partners who also participated as a member of trial management group and trial steering committee. Our patient partners reviewed study design, paying attention to acceptability of randomisation analgesic techniques in perioperative setting and burden of study participation. Study results including plan for future study has been disseminated within local and national patient research ambassador groups.

RESULTS

Participants and follow-up

564 patients were screened for eligibility from July 2015 to July 2016 - 100% of those listed for elective thoracotomy - with 194 patients found to be eligible (Figure 1). The most common reasons for ineligibility were: undergoing video-assisted thoracoscopic surgery (174/370, 47%); participation in another study which did not allow co-enrolment (59/370, 16%); previous thoracotomy (31/370, 8%). Of the 194 patients, 69 (36%, 95%CI: 29% to 42%) were ultimately randomised. Of the others, the most common reasons for non-randomisation were: anaesthetists trained to perform PVB not available (24/125, 19%); did not want to be randomised (15/125, 12%); patient had preference for PVB (15/125, 12%). Details of randomised participants are given in Table 1. The minimisation algorithm provided appropriate balance for the balancing factors; the average age of participants was 66 years with the vast majority were having a lung cancer resection operation (60/69, 87%). Seven participants (10%) reported previous problems with (unspecified) chronic pain.

At six months post-randomisation, 54 of 63 (86%) questionnaire booklets were returned, discounting 5 participants who died during the follow-up period and one participant who withdrew from the study. Of these, only one did not complete their VAS questions.

For peer review only

Table 1. Baseline characteristics of patients

For peer review only

		PVB (n=35)	TEB (n=34)
Age >=65, years[1]		21 (60)	20 (59)
Age, years	Mean (SD)	66.0 (10.6)	65.9 (7.9)
Gender=male[1]		19 (54)	20 (59)
Centre[1]	Birmingham Heartlands	23 (66)	26 (76)
	University Hospital South Manchester	12 (34)	8 (24)
Reason for thoracotomy=lung cancer resection[1]		30 (86)	30 (88)
ASA[2] physical status	1: Normal healthy	0 (-)	2 (6)
	2: Mild systemic disease	13 (37)	10 (30)
	3: Severe systemic disease	22 (63)	21 (64)
	Missing	-	1
ECOG[3] performance status	0: Normal activity	20 (57)	19 (58)
	1: Symptomatic but nearly fully ambulatory	14 (40)	14 (42)
	2: Symptomatic but bed <50% daytime	1 (3)	0 (-)
	3: Symptomatic bed>50% daytime	0 (-)	0 (-)
	4: Unable to get out of bed	0 (-)	0 (-)
	Missing	-	1
Dyspnoea	0: None	17 (48)	17 (52)
	1: Slight	16 (46)	13 (39)
	2: Moderate	2 (6)	1 (3)
	3: Moderately severe	0 (-)	2 (6)
	4: Severe	0 (-)	0 (-)
	5: Very severe	0 (-)	0 (-)
	Missing	-	1
Smoking status	Never smoked	7 (20)	6 (18)
	Stopped>1 year	19 (54)	18 (55)
	Stopped>6 weeks	3 (9)	3 (9)
	Stopped<6 weeks	1 (3)	4 (12)
	Current smoker	5 (14)	2 (6)
	Missing	-	1
Smoking-pack years	Median [IQR]	37 [20, 49]	37 [20, 50]
	Missing	-	1
Alcohol units per week	Median [IQR]	3 [0, 13.5]	0 [0, 9.7]
	Missing	-	1
Previous medical history=yes	COPD	7 (20)	6 (18)
	Ischaemic heart disease	4 (11)	3 (9)
	Congestive cardiac failure	0 (-)	1 (3)
	Hypertension	18 (51)	15 (45)
	Diabetes diet controlled	0 (-)	3 (9)
	Diabetes oral medication	6 (17)	3 (9)
	Diabetes insulin controlled	2 (6)	1 (3)
	Renal failure	1 (3)	1 (3)
	Previous stroke	0 (-)	0 (-)
	Hyperthyroidism	0 (-)	0 (-)
	Hypothyroidism	5 (14)	2 (6)
	Other cancer	12 (34)	7 (21)
	Chronic pain	2 (6)	5 (15)
	Missing	-	1
BMI, kg/m^2	Mean (SD)	28.4 (5.8)	27.7 (5.9)
	Missing	-	1

For peer review only

Frequency (%) presented (unless otherwise stated)

[1] minimisation variable; [2] American Society of Anesthesiologists; [3] Eastern Cooperative Oncology Group

For peer review only

Analgesic technique

Of the 69 randomised, 67 (97%) received their randomised intervention. One participant allocated PVB received TEB as the pleura was removed and the surgeon was unable to place the PVB. Another participant was not considered fit for surgery and was withdrawn from the study. Discounting this participant, all anaesthetic technique and intra-operative forms were completed. A summary of the anaesthetic techniques is given in Table 2; a summary of the operative details and other analgesics provided to participants up to discharge is provided in the Appendix (Tables S1, S2).

For peer review only

Table 2. Anaesthetic technique summary

		**PVB (n=35)**	**TEB (n=33)[1]**
Difficulty in insertion		4 (12)[2]	14 (42)
Failure to deliver technique		1 (3)	0 (-)
Intraoperative use			
Preoperative bolus given		26 (74)	25 (76)
Bolus drug given through catheter		14 (40)	24 (73)
Infusion given through catheter		23 (66)	21 (64)
Post-operative settings			
Local anaesthetic was Bupivacaine (as opposed to Levobupivacaine)		32 (94)[2]	19 (58)
Concentration	0.1%	20 (59)[2]	11 (33)
	0.125%	4 (12)[2]	22 (67)
	0.25%	10 (29)[2]	0 (-)
Fentanyl	None	32 (91)	3 (9)
	2 mcg/ml	0 (-)	0 (-)
	4 mcg/ml	3 (9)	30 (91)
Starting rate, ml/hr	Median [IQR]	20 [10, 20][2]	6 [6, 8]
Prescribed range, ml/hr	Median [IQR]	20 [15, 20][2]	12 [8, 15]

Frequency (%) presented (unless otherwise stated)

[1] one participant received neither technique as they did not have an operation; [2] one response missing (n=34)

Patient reported outcomes

Levels of pain in the three days post-operation appeared similar in both groups (Appendix Table S3). The number of patients indicating at least a moderate level of average or worst chest pain (based on commonly used threshold of >3 or ≥4[23, 24]) was lower with PVB compared to TEB at six months, but with expected high levels of uncertainty given the limited size of sample (Table 3; full results given in Appendix Table S4). Results from other questionnaire responses had a lot of uncertainty (Table 4).

Table 3. Incidence of significant (>3 or >=4) or severe pain (>=7) from Visual Analogue Scale Scores

	PVB n (%)	TEB n (%)	RR (95% CI) [1]
Average chest pain overall>3			
3 months	5/25 (20.0)	6/21 (28.6)	0.7 (0.2, 2.0)
**6 months**	2/27 (7.4)	5/25 (20.0)	0.4 (0.1, 1.7)
Average chest pain overall>=4			
3 months	5/25 (20.0)	4/21 (19.0)	1.1 (0.3, 3.4)
**6 months**	1/27 (3.7)	3/25 (12.0)	0.3 (0.03, 2.8)
Average chest pain overall>=7			
3 months	0/25 (-)	2/21 (10.0)	- [2]
**6 months**	0/27 (-)	1/25 (4.0)	- [2]
Worst chest pain overall>3			
3 months	9/24 (37.5)	7/23 (30.4)	1.2 (0.6, 2.8)
**6 months**	7/27 (25.9)	11/26 (42.3)	0.6 (0.3, 1.3)
Worst chest pain overall>=4			
3 months	8/24 (33.3)	7/23 (30.4)	1.1 (0.5, 2.5)
**6 months**	7/27 (25.9)	9/26 (34.6)	0.75 (0.3, 1.7)
Worst chest pain overall>=7			
3 months	6/24 (25.0)	5/23 (21.7)	1.2 (0.4, 3.3)
**6 months**	2/27 (7.4)	5/26 (19.2)	0.4 (0.1, 1.8)

[1] RR<0 indicate less incidence with PVB; [2] unable to estimate

For peer review only

Table 4. Results of patient reported outcomes

	PVB mean score (SD, n)	TEB mean score (SD, n)	Difference between groups (95% CI)
**Brief pain inventory (BPI) results summary[1]**			
Pain severity score (0-10, higher=worse pain)			
Baseline	0.9 (1.7, 34)	1.3 (2.0, 32)	-0.4 (-1.3, 0.5)
24 hours post-surgery	5.3 (1.7, 33)	3.9 (2.7, 32)	1.4 (0.3, 2.5)
48 hours post-surgery	5.2 (1.7, 30)	5.1 (2.5, 31)	0.1 (-0.9, 1.2)
72 hours post-surgery	4.9 (1.7, 23)	5.6 (2.0, 25)	-0.7 (-1.8, 0.4)
Discharge	4.0 (1.4, 31)	4.1 (2.4, 32)	-0.1 (-1.1, 0.9)
3 months	1.6 (1.7, 22)	2.5 (2.5, 21)	0.9 (-2.2, 0.4)
**6 months**	1.3 (1.8, 25)	2.3 (2.6, 23)	-1.0 (-2.3, 0.3)
Pain interference score (0-10, higher=worse pain)			
Baseline	0.6 (1.3, 34)	1.5 (2.2, 32)	-0.9 (-1.8, -0.1)
24 hours post-surgery	5.8 (2.1, 30)	5.4 (2.9, 28)	0.4 (-0.8, 1.8)
48 hours post-surgery	6.0 (2.0, 30)	5.8 (2.2, 27)	0.2 (-0.9, 1.3)
72 hours post-surgery	5.4 (2.3, 21)	5.8 (2.6, 23)	-0.4 (-1.9, 1.1)
Discharge	4.7 (2.0, 23)	4.3 (2.5, 29)	0.4 (-0.9, 1.7)
3 months	1.1 (1.4, 24)	2.8 (3.0, 22)	-1.7 (-3.1, -0.3)
**6 months**	1.6 (2.2, 27)	1.9 (2.5, 25)	-0.3 (-1.6, 1.0)
**Neuropathic pain scale (NPS) results summary[1]**			
How intense does your pain feel? (0-10, higher=worse pain)			
Baseline	1.7 (2.8, 33)	1.8 (2.8, 33)	-0.1 (-1.5, 1.3)
Discharge	5.8 (2.8, 31)	5.4 (3.8, 31)	0.4 (-1.3, 2.1)
3 months	2.2 (2.4, 25)	3.6 (3.8, 21)	-1.4 (-3.4, 0.5)
**6 months**	2.2 (2.7, 27)	2.7 (3.2, 26)	-0.5 (-2.2, 1.1)
How sharp does your pain feel? (0-10, higher=worse pain)			
Baseline	1.1 (2.8, 33)	1.6 (3.4, 32)	-0.5 (-2.1, 1.1)
Discharge	5.5 (3.2, 31)	5.4 (4.0, 31)	0.1 (-1.7, 1.9)
3 months	2.6 (3.0, 25)	3.2 (4.0, 21)	-0.6 (-2.8, 1.5)
**6 months**	3.0 (3.3, 27)	2.4 (3.3, 26)	0.6 (-1.3, 2.4)
How hot does your pain feel? (0-10, higher=worse pain)			
Baseline	1.0 (2.6, 33)	1.3 (2.7, 32)	-0.3 (-1.6, 1.0)
Discharge	3.6 (3.4, 31)	2.4 (2.9, 31)	1.2 (-0.4, 2.8)
3 months	1.6 (2.8, 25)	2.5 (3.3, 21)	-0.9 (-2.7, 1.0)
**6 months**	1.2 (2.0, 28)	1.6 (2.9, 26)	-0.4 (-1.7, 1.0)
How dull does your pain feel? (0-10, higher=worse pain)			
Baseline	1.8 (2.9, 33)	1.5 (2.5, 33)	0.3 (-1.0, 1.7)
Discharge	2.9 (2.7, 30)	3.9 (2.8, 30)	-1.0 (-2.4, 0.5)
3 months	2.6 (2.1, 25)	3.3 (3.3, 21)	-0.7 (-2.4, 0.9)
**6 months**	2.1 (2.8, 28)	2.3 (2.8, 26)	-0.2 (-1.8, 1.2)
How cold does your pain feel? (0-10, higher=worse pain)			
Baseline	0.3 (1.6, 33)	0.4 (1.7, 32)	-0.1 (-0.9, 0.7)
Discharge	1.5 (2.5, 30)	1.0 (2.2, 31)	0.5 (-0.7, 1.7)
3 months	0.2 (0.5, 25)	1.5 (2.9, 21)	-1.3 (-2.7, -0.0)
**6 months**	0.3 (0.9, 28)	0.7 (1.6, 26)	-0.4 (-1.1, 0.3)
How sensitive is your pain? (0-10, higher=worse pain)			
Baseline	0.5 (1.9, 33)	1.1 (2.5, 32)	-0.6 (-1.7, 0.5)
Discharge	2.3 (2.9, 30)	2.8 (2.9, 32)	-0.5 (-1.9, 1.0)
3 months	2.2 (3.0, 25)	2.1 (2.7, 23)	0.1 (-1.6, 1.7)
**6 months**	1.6 (2.0, 28)	2.5 (3.1, 26)	-0.9 (-2.4, 0.5)
How itchy is your pain? (0-10, higher=worse pain)			
Baseline	0 (0, 33)	0.8 (2.0, 32)	-0.8 (-1.5, -0.0)
Discharge	2.2 (2.8, 30)	2.6 (3.3, 31)	-0.4 (-2.0, 1.2)
3 months	1.2 (2.5, 25)	1.8 (2.6, 22)	-0.6 (-2.1, 0.9)
**6 months**	0.4 (0.7, 28)	2.1 (2.6, 25)	-1.7 (-2.8, -0.6)

For peer review only

How unpleasant is your pain? (0-10, higher=worse pain)			
Baseline	1.9 (2.9, 31)	2.1 (3.3, 33)	-0.2 (-1.7, 1.4)
Discharge	6.2 (3.0, 30)	5.6 (3.3, 30)	0.6 (-1.1, 2.2)
3 months	2.8 (2.6, 24)	4.0 (3.9, 21)	-1.2 (-3.3, 0.8)
**6 months**	2.4 (2.7, 28)	3.0 (3.2, 24)	0.6 (-2.3, 1.0)
How intense is your deep pain? (0-10, higher=worse pain)			
Baseline	1.3 (2.4, 32)	2.4 (3.7, 33)	-1.1 (-2.6, 0.5)
Discharge	6.2 (2.8, 31)	5.5 (3.4, 31)	0.7 (-0.9, 2.3)
3 months	3.0 (2.8, 23)	4.1 (3.7, 21)	-1.1 (-3.1, 0.9)
**6 months**	2.8 (3.0, 28)	2.5 (2.9, 25)	0.3 (-1.4, 1.9)
How intense is your surface pain? (0-10, higher=worse pain)			
Baseline	0.8 (2.1, 31)	0.7 (1.5, 32)	0.1 (-0.9, 1.0)
Discharge	3.9 (3.4, 30)	3.8 (3.3, 32)	0.1 (-1.7, 1.7)
3 months	1.9 (2.2, 24)	3.5 (3.6, 21)	-1.6 (-3.3, 0.2)
**6 months**	1.2 (1.5, 27)	2.7 (3.2, 24)	-1.5 (-2.9, -0.1)
**EQ-5D-DL results summary[2]**			
EQ-5D index score (-0.59=worst outcome, 1.0=best outcome)			
Baseline	0.75 (0.22, 35)	0.77 (0.20, 33)	-0.02 (-0.12, 0.08)
Discharge	0.49 (0.23, 27)	0.49 (0.23, 30)	-0.00 (-0.12, 0.12)
3 months	0.73 (0.17, 25)	0.65 (0.23, 23)	0.08 (-0.04, 0.20)
**6 months**	0.72 (0.23, 25)	0.69 (0.22, 25)	0.03 (-0.09, 0.16)
EQ-5D thermometer (0-100, higher=better)			
Baseline	76.6 (16.3, 34)	74.3 (17.8, 33)	2.3 (-5.9, 10.7)
Discharge	60.4 (22.7, 26)	60.2 (20.7, 30)	0.2 (-11.5, 11.9)
3 months	73.2 (21.2, 25)	67.5 (18.3, 23)	5.7 (-5.8, 17.2)
**6 months**	78.0 (14.0, 25)	64.4 (20.4, 25)	13.6 (3.6, 23.6)
**Hospital anxiety and depression index score (HADS) results summary[3]**			
Depression index score (0-21, lower=better)			
Baseline	2.9 (2.9, 33)	3.0 (2.6, 32)	-0.1 (-1.4, 1.3)
Discharge	4.3 (3.4, 28)	6.0 (5.0, 28)	-1.7 (-4.0, 0.5)
3 months	3.6 (2.7, 25)	5.5 (4.0, 21)	-1.9 (-4.1, 0.1)
**6 months**	4.5 (3.5, 24)	6.0 (4.6, 25)	-1.5 (-3.9, 0.8)
Anxiety index score (0-21, 0-lower=better)			
Baseline	6.3 (5.0, 34)	6.5 (4.4, 30)	-0.2 (-2.6, 2.1)
Discharge	5.0 (4.4, 29)	7.0 (5.3, 28)	-2.0 (-4.6, 0.6)
3 months	5.2 (4.3, 25)	6.1 (4.8, 19)	-0.9 (-3.7, 1.9)
**6 months**	5.7 (3.6, 24)	7.3 (5.0, 25)	-1.6 (-4.1, 0.9)

[1] scores<0 indicate less pain with PVB

[2] scores>0 indicate better QoL with PVB

[3] scores<0 indicate less anxiety/depression with PVB

For peer review only

Safety

No concerns were expressed by the independent Oversight Committee who met twice during the recruitment period to review study progress and safety data. Complications and short-term mortality data appeared similar in both groups (Appendix Table S5). There were five deaths; only one was considered to be a serious adverse event, this was aspiration pneumonitis and was not considered related to intervention (PVB). One other participant experienced a serious adverse event in the TEB group; this participant was diagnosed with acute kidney injury in the post-operative period.

Assessment of participant blinding

19/32 (60%) patients in the TEB group correctly identified the anaesthetic technique they had been allocated at discharge; the figure for PVB was 8/31 (26%); this reduced to 13/24 (52%) in the TEB group at 6 months and 5/26 (19%) in the PVB group (Appendix Table S6).

National survey of anaesthetists

Forty-three responses were returned. Of these, 27 anaesthetists indicated that they would be willing to randomise in a future study (63%). Five (12%) indicating that they would 'possibly' be willing to randomise with reasons of concern given as lack of skill and ward structure. The remaining 11 (26%) were unwilling.

Qualitative interviews

Interviewed participants had a positive experience, reporting that they felt well informed by trial staff. The consent process was felt to be well undertaken and patients felt reassured by the explanations of randomisation. A substantial number of participants reported that the study questionnaires were long and repetitive. Interviewed staff felt recruitment was successful although there were some challenges when the role was undertaken by non-clinical staff. There was a feeling that some of the data collection tools were repetitive. Clinical staff were supportive of the trial; they found that the trial processes, randomisation and procedures were very straightforward. The guidance and teaching provided for anaesthetists to get them up to speed for performing PVB was very positively received and it was considered a relatively simple procedure to learn.

DISCUSSION

Our four key indicators for feasibility were met and we have shown that a large multicentre randomised controlled trial of PVB versus TEB for peri-operative pain control with an objective of assessing long-term

For peer review only

post-thoracotomy chronic pain is feasible. It is possible to randomise and follow up patients with high fidelity over six months. In the qualitative assessment we carried out, participants and staff were largely happy with study processes. In particular, the consent, randomisation and assessment methods were considered appropriate. As hypothesised, VAS pain scores were lower with PVB compared with TEB at six months on average but with high levels of uncertainty. This would need to be investigated further in a much more substantial trial.

Whilst the evidence on short-term outcomes, e.g. minor complications and analgesic efficacy, point to PVB being at least as effective as TEB[14], current evidence on whether PVB is more effective than TEB in reducing rates of chronic pain is limited. A recent systematic review failed to attain sufficient data to compare the impact of TEB and PVB techniques on CPTP. [14] The incidence of CPTP was very poorly reported and studies did not describe how data on chronic pain was measured or collected. The lack of evidence highlights an area in need of further research. We are not aware of any current or planned randomised trials with CPTP as an outcome that may change these findings since this systematic review was published. Incidence of CPTP was slightly lower in our study - between 30-34% - than previously quoted at 49%[6], although we need to acknowledge that our figure is associated with a reasonably large amount of uncertainty given the small size of sample. A likely contributing factor to this discrepancy is the considerable and widely acknowledged variation in how pain is measured[25], as it is dependent on the measurement instrument and threshold chosen amongst other factors.[23]

This feasibility work has allowed us to fine tune study processes ahead of a larger trial. One likely modification to the future study protocol is to revise the length of the follow-up questionnaires, it was considered by participants that their length was too long and repetitive in content. Shortened and simplified follow-up questionnaire would also potentially helping to improve follow-up figures further, as lengthy questionnaires are a known predictor of participant drop-out from trials.[26]

Inclusion and exclusion criteria appeared appropriate and do not appear to need to be changed. The vast majority of ineligible participants were those scheduled to receive video-assisted thoracoscopic surgery (VATS). Whilst this procedure is becoming more popular[1], the comparison in question is not an appropriate one as TEB is not routinely used to provide pain relief for in this type of surgery. The VATS approach also does not require any rib spreading, resulting in a less invasive procedure, and whilst chronic pain is still an issue after VATS it is not considered to be as great as following open thoracotomy.[27] Unfortunately, whilst VATS is becoming more common, there will still be a substantial proportion of patients that will always be ineligible for this operation because of, for example, inability to achieve

For peer review only - http://bmjopen.bmj.com/site/about/guidelines.xhtml

complete resection in more advanced pulmonary lung cancers. Lung cancer remains the most common cancer worldwide.[28,29]

It was apparent from the assessment of the ability to blind the participant to the analgesic technique that complete blinding is unlikely to be possible. Substantially more participants appeared to be able to determine they had had an epidural blockade than the paravertebral technique. Attempts to completely blind participants in any future study are likely to make processes prohibitively complex and expensive due to innate differences in the mode of delivery and action in the two analgesic techniques. This could include sham insertion techniques to disguise the randomised intervention. The question is whether patient blinding is likely to be an important potential cause of detection bias, unduly affecting the results of any future trial. The proposed primary outcome of pain rating is subjective in nature and this is a theoretical possibility. However, we believe that there is no reason to suspect that recipients of the randomised interventions have strong pre-conceptions with regard to the relative effectiveness of each analgesic technique. Furthermore, the primary outcome would be collected via questionnaires administered by post or phone, at a time remote from the original operative procedure, which are likely to be resilient to the effects of imperfect concealment.

Findings from the survey of practice were encouraging, although whilst we set a target of 70% of thoracic anaesthetists willing to randomise to a future trial as being indicative of success, we found 63% willing and 12% 'possibly willing'. To achieve 70%, we would require solutions for those anaesthetists expressing uncertainty. The major reasons given were lack of skill and ward structure. These hurdles do not appear insurmountable if funding for further training and extra nurse support could be provided; something a large funding body may be willing to provide. A successful trial would also require full engagement with anaesthetic and thoracic surgical trainees' network to maximise recruitment.

We have reported the study to randomised pilot study standards as recommended by CONSORT extension for pilot study.[30] Also as recommended,[31] we set objectives a priori to determine whether the pilot study would demonstrate feasibility of a larger trial. Since this study was not a definitive trial, we have been careful not to over-interpret the findings. Hence at this stage, we cannot expect that the outcomes of this pilot study to be directly translated into clinical care, as the study was not large enough to be able to detect small to moderate realistic-sized difference in rates of chronic pain, nor was the scope wide enough in terms of the number of centres involved to return a generalisable result.

Nevertheless, this pilot study is an important precursor to a larger, substantive, trial and provides invaluable information that will help to ensure its success. A definitive study - instigated by the authors of

For peer review only

this manuscript - has now been funded by the NIHR Health Technology Assessment Programme and is expected to start recruiting in 2018 from 20 UK thoracic centres. The design of this study has been partially informed by the results of this pilot. The primary outcome will be incidence of chronic pain at six months attained from visual analogue scale responses similar to that used here; other outcomes will follow IMMPACT recommendations.[32] A total sample size of 1026 participants will give 90% power (p=0.05) to detect a reduction in incidence of CPTP of 30% with TEB (a similar event rate to that noted in this pilot study) down to 20% with PVB. The rate of recruitment noted in this study has been factored into the number of sites and length of the recruitment period required; we estimate access to a pool of 5000 participants over a two and a half year recruitment period and need to recruit a fifth of these. The design will allow a definitive answer this important question.

AUTHOR'S CONTRIBUTION

FG, JY, JPD and MW conceived the topic. FG, JY, JPD, LJM and AG designed the study with the input from SK, LS, BN, MW, TM and AW. FG oversaw the running of the trial and all the authors contributed to the ongoing management of the trial. AK, RS, BN collected data for the trial. KT performed the statistical analysis. SF evaluated the qualitative data. The manuscript was drafted by LJM, JY and FG with contributions from the other authors. All the authors contributed to the interpretation of the output and revised and reviewed the paper; they are the guarantors. The Birmingham Clinical Trials Unit undertook the randomisation and data management and monitoring. The authors had full access to all the data from the study. The authors vouch for the accuracy and completeness of the data and analyses. All authors read and approved the final manuscript.

ACKNOWLEDGEMENTS

We thank the trial participants; members of the trial Oversight Committee: Tom Treasure (Chair), Alistair Macfie (independent anaesthetist), Mahmoud Loubani (independent surgeon), Lucy Bradshaw (independent statistician), Alistair Murray (patient and public representative); the Clinical Research Ambassador Group at Heart of England NHS Foundation Trust; Birmingham Clinical Trials Unit staff: Matt Hill (database programmer) and Hannah Watson (data manager).

COMPETING INTERESTS

All authors declare that they have no competing interests.

For peer review only

FUNDING

The study was funded by a grant from the National Institute for Health Research for Patient Benefit (RFPB grant number PB-PG-0213-30126). The funder had no role in study design, data collection, interpretation or analysis, or in writing the report for publication. S F is funded by the National Institute of Health Research (NIHR) Collaboration for Leadership in Applied Health Research and Care West Midlands (CLAHRCWM).

SPONSORSHIP

The study sponsor was the Heart of England NHS Foundation Trust. The sponsor had no role in study design, data collection, interpretation or analysis, or in writing the report for publication.

DATA SHARING STATEMENT

All available study data can be obtained from the corresponding author.

For peer review only

References

1. The Society for Cardiothoracic Surgery in Great Britain and Ireland. SCTS 2014-2015 Thoracic Registry Data. 2015.

2. Hughes R, Gao F. Pain control for thoracotomy. *Continuing Education in Anaesthesia, Critical Care and Pain.* 2005;5(2):56-60.

3. Soto RG, Fu ES. Acute pain management for patients undergoing thoracotomy. *Ann Thorac Surg.* 2003;75(4):1349-1357.

4. Kavanagh BP, Katz J, Sandler AN. Pain control after thoracic surgery. A review of current techniques. *Anesthesiology.* 1994;81(3):737-759.

5. International Association for the Study of Pain. Classification of chronic pain. Descriptions of chronic pain syndromes and definitions of pain terms. Prepared by the International Association for the Study of Pain, Subcommittee on Taxonomy. *Pain Supplement.* 1986;3:S1-226.

6. Bayman EO, Brennan TJ. Incidence and severity of chronic pain at 3 and 6 months after thoracotomy: meta-analysis. *The Journal of Pain.* 2014;15(9):887-897.

7. Dahl JB, Rosenberg J, Lund C, Kehlet H. Effect of thoracic epidural bupivacaine 0.75% on somatosensory evoked potentials after dermatomal stimulation. *Reg Anesth.* 1990;15(2):73-75.

8. Lund C, Hansen OB, Mogensen T, Kehlet H. Effect of thoracic epidural bupivacaine on somatosensory evoked potentials after dermatomal stimulation. *Anesth Analg.*1987;66(8):731-734.

9. Richardson J, Lönnqvist PA. Thoracic paravertebral block. *Br J Anaesth.* 1998;81(2):230-238.

10. Cook E, Downs C. Analgesia After Thoracotomy - The Role of the Extrapleural Paravertebral Catheter. *Australian Anaesthesia.* 2005:103-117.

11. Richardson J, Jones J, Atkinson R. The effect of thoracic paravertebral blockade on intercostal somatosensory evoked potentials. *Anesthesia & Analgesia.* 1998;87(2):373-376.

12. Karmakar MK, Samy W, Li JW, et al. Thoracic paravertebral block and its effects on chronic pain and health-related quality of life after modified radical mastectomy. *Reg Anesth Pain Med.* 2014;39(4):289-298.

13. Turner J, Yeung J. Analgesic technique in thoracic surgery: a survey of UK ACTACC members. Unpublished data.2016.

14. Yeung JH, Gates S, Naidu BV, Wilson MJ, Gao Smith F. Paravertebral block versus thoracic epidural for patients undergoing thoracotomy. *Cochrane Database Syst Rev.* 2016;2:CD009121.

For peer review only

15. Yeung J, Melody T, Kerr A, Naidu B, Middleton L, Tryposkiadis K, Daniels J, Gao F. Randomised controlled pilot study to investigate the effectiveness of thoracic epidural and paravertebral blockade in reducing chronic post-thoracotomy pain: TOPIC feasibility study protocol. BMJ Open. 2016 Dec 1;6(12):e012735. doi: 10.1136/bmjopen-2016-012735.

16. Jensen MP, Karoly P, Braver S. The measurement of clinical pain intensity: a comparison of six methods. Pain. 1986 Oct;27(1):117-26.

17. Tan G, Jensen MP, Thornby JI, et al. Validation of the Brief Pain Inventory for chronic nonmalignant pain. J Pain 2004;5:133–7.

18. Galer BS, Jensen MP. Development and preliminary validation of a pain measure specific to neuropathic pain: the Neuropathic Pain Scale. Neurology 1997;48:332–8.

19. Herdman M, Gudex C, Lloyd A, et al. Development and preliminary testing of the new five-level version of EQ-5D (EQ-5D-5L). Qual Life Res 2011;20:1727–36.

20. Zigmond AS, Snaith RP. The hospital anxiety and depression scale. Acta Psychiatr Scand. 1983;67:361–70.

21. Moore CG, Carter RE, Nietert PJ, Stewart PW. Recommendations for planning pilot studies in clinical and translational research. Clin Transl Sci. 2011 Oct;4(5):332-7. doi: 10.1111/j.1752-8062.2011.00347.x.

22. Pope C, Ziebland S, Mays N. Qualitative research in health care. Analysing qualitative data. *BMJ* 2000;320(7227):114-6.

23. Jensen MP, Chen C, Brugger AM. Interpretation of visual analog scale ratings and change scores: a reanalysis of two clinical trials of postoperative pain. J Pain. 2003;4:407-14.

24. Palos GR, Mendoza TR, Mobley GM, Cantor SB, Cleeland CS. Asking the community about cutpoints used to describe mild, moderate, and severe pain. J Pain. 2006 Jan;7(1):49-56.

25. Litcher-Kelly L, Martino SA, Broderick JE, Stone AA. A systematic review of measures used to assess chronic musculoskeletal pain in clinical and randomized controlled clinical trials. The journal of pain: official journal of the American Pain Society. 2007;8(12):906-913.doi:10.1016/j.jpain.2007.06.009.

26. Edwards P, Roberts I, Sandercock P, Frost C. Follow-up by mail in clinical trials: does questionnaire length matter? Control Clin Trials. 2004 Feb;25(1):31-52

27. Bendixen M, Jørgensen OD, Kronborg C, Andersen C, Licht PB. Postoperative pain and quality of life after lobectomy via video-assisted thoracoscopic surgery or anterolateral thoracotomy for early stage lung cancer: a randomised controlled trial. Lancet Oncol. 2016 Jun;17(6):836-844. doi: 10.1016/S1470-2045(16)00173-X. Epub 2016 May 6.

28. Ferlay J, Soerjomataram I, Ervik M, Dikshit R, Eser S, Mathers C et al. GLOBOCAN 2012 v1.0, Cancer Incidence and Mortality Worldwide: IARC Cancer Base No. 11.

For peer review only

29. Stewart BW, Wild CP, editors. World cancer report 2014. Lyon: International Agency for Research on Cancer; 2014.

30. Eldridge SM, Chan CL, Campbell MJ, Bond CM, Hopewell S, Thabane L, Lancaster GA. CONSORT 2010 statement: extension to randomised pilot and feasibility trials. BMJ. 2016 Oct 24;355:i5239. doi: 10.1136/bmj.i5239.

31. Thabane L, Ma J, Chu R, Cheng J, Ismaila A, Rios LP, Robson R, Thabane M, Giangregorio L, Goldsmith CH. A tutorial on pilot studies: the what, why and how. BMC Medical Research Methodology201010:1 https://doi.org/10.1186/1471-2288-10-1.

32. Dworkin RH, Turk DC, Farrar JT, Haythornthwaite JA, Jensen MP, Katz NP et al. Core outcome measures for chronic pain clinical trials: IMMPACT recommendations. Pain. 2005; 113:9-19.

For peer review only

LEGEND FOR FIGURE 1

Figure 1. Flow chart of patients through the study

For peer review only

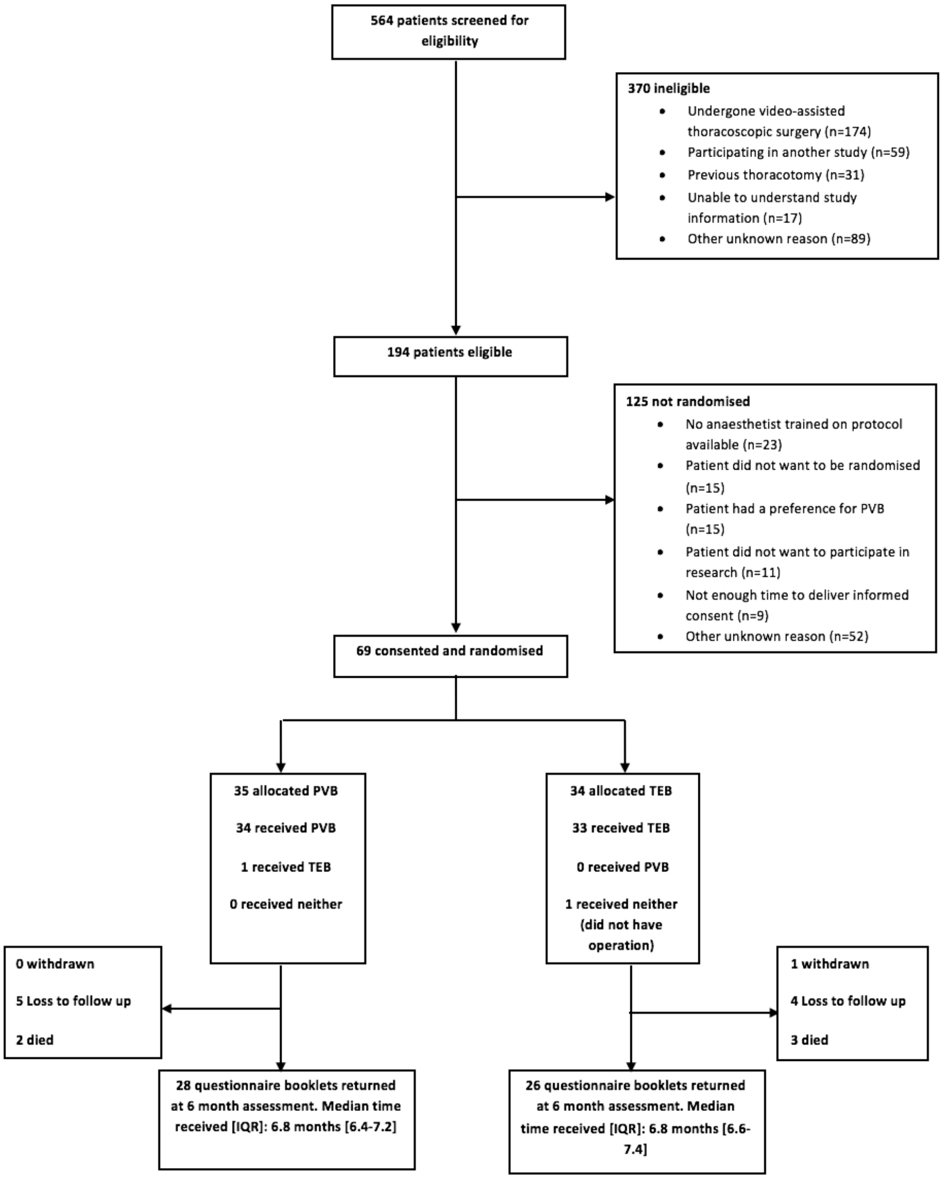

Figure 1

79x98mm (300 x 300 DPI)

TOPIC PILOT STUDY SUPPLEMENTARY APPENDIX

Table S1: Intraoperative data summary 2

Table S2: Analgesic use summary 3

Table S3: Post-operative (in hospital) pain scores summary 4

Table S4: Comprehensive summary of Visual Analogue Scale (VAS) scores 5

Table S5: Complications, short-term mortality and length of stay summary 7

Table S6: Patient satisfaction and blinding questions results summary 8

For peer review only

Table S1: Intraoperative data summary

		PVB (n=35)	TEB (n=33)[1]
Vasopressor/inotrope used		30 (86)	27 (82)
Delivery route	Boluses	24 (80)	24 (89)
	Infusion	3 (10)	1 (4)
	Both	3 (10)	2 (7)
Overall fluid therapy and balance	Median [IQR]	1000 [1000, 2000]	1000 [1000, 1500]
Grade of surgeon was consultant (as opposed to trainee)		21 (60)	18 (55)
Side of operation was left (as opposed to right)		9 (26)	10 (30)
Type of operation	Lobectomy	18 (51)	18 (55)
	Pneumonectomy	3 (9)	2 (6)
	Segmentectomy	1 (3)	1 (3)
	Wedge	3 (9)	2 (6)
	Lung biopsy	1 (3)	0 (-)
	Wedge and Segmentectomy	0 (-)	1 (3)
	Lobectomy and Wedge	1 (3)	0 (-)
	Lobectomy and Other	2 (6)	2 (6)
	Other	6 (17)	7 (21)
Surgical approach	Anterolateral	4 (12)[2]	2 (6)
	Posterolateral	22 (65)[2]	23 (70)
	Muscle sparing	0 (-)[2]	1 (3)
	Mini-thoracotomy	1 (3)[2]	0 (-)
	Posterolateral and Muscle sparing	7 (21)[2]	6 (18)
	Mini-limited lateral	0 (-)[2]	1 (3)

Frequency (%) presented (unless otherwise stated)
[1] one participant received neither technique as they did not have an operation; [2] one response missing (n=34)

For peer review only

For peer review only - http://bmjopen.bmj.com/site/about/guidelines.xhtml

Table S2: Analgesic use summary

			In Hospital (post-surgery)							
	Intraoperative		Day 1		Day 2		Day 3		Between day 3 and discharge/final assessment	
**Drug used**	**PVB**	**TEB**	**PVB**	**TEB**	**PVB**	**TEB**	**PVB**	**TEB**	**PVB**	**TEB**
Paracetamol	29	29	1	-	-	-	-	-	-	-
Diclofenac	3	-	3	2	-	-	-	-	-	-
Remifentanil	-	-	6	5	2	-	-	-	-	-
Fentanyl	-	-	16	11	5	4	2	-	-	-
Morphine	2	-	6	1	14	3	3	2	2	-
Diamorphine	-	2	-	10	1	7	-	1	-	-
Gabapentin	-	-	-	-	-	-	-	1	-	-
Alfentanil	-	-	1	2	3	10	1	1	-	-
Bupivicaine	-	-	-	-	-	-	-	1	-	-
Levobupivicaine	-	-	-	-	-	1	-	-	-	-
Pregabalin	-	-	-	-	-	-	1	-	-	-
Ketorolac	-	-	-	-	-	2	3	2	2	-

Number of participants administered drug indicated (total number given PVB=35 and total number given TEB=33)

For peer review only

Table S3: Post-operative (in hospital) pain scores summary

	PVB mean score (SD, n)	TEB mean score (SD, n)	Difference between groups (95% CI) [1]
Pain score at rest (0-3, higher=worse pain)			
Day 1	0.4 (0.7, 34)	0.5 (1.0, 33)	-0.1 (-0.5, 0.3)
Day 2	0.5 (0.7, 33)	0.7 (1.0, 32)	-0.2 (-0.6, 0.3)
Day 3	0.2 (0.4, 27)	0.6 (0.7, 27)	-0.4 (-0.8, -0.1)
Pain score at movement (0-3, higher=worse pain)			
Day 1	0.9 (0.9, 34)	0.8 (1.0, 33)	0.1 (-0.3, 0.6)
Day 2	1.2 (0.8, 33)	1.3 (0.9, 32)	-0.1 (-0.5, 0.4)
Day 3	0.8 (0.9, 27)	1.0 (1.0, 27)	-0.2 (-0.8, 0.3)

[1] scores<0 indicate less pain with PVB

For peer review only

Table S4: Comprehensive summary of Visual Analogue Scale (VAS) scores

	PVB mean score (SD, n)	TEB mean score (SD, n)	Difference between groups (95% CI) [1]
Average chest pain overall (0-10, higher=worse pain)			
Baseline	0.6 (1.8, 32)	0.7 (1.7, 33)	-1.0 (-1.0, 0.8)
24 hours post-surgery	5.2 (2.5, 33)	3.7 (3.4, 32)	1.5 (-0.02, 2.9)
48 hours post-surgery	6.4 (1.8, 31)	5.5 (2.7, 31)	0.9 (-0.3, 2.1)
72 hours post-surgery	5.1 (25, 2.4)	6.1 (2.5, 26)	-1.0 (-2.4, 0.3)
Discharge	4.9 (1.9, 29)	4.3 (2.5, 32)	0.6 (-0.5, 1.7)
3 months	2.1 (1.8, 25)	2.0 (2.6, 21)	0.1 (-1.3, 1.4)
**6 months**	0.8 (1.2, 27)	1.7 (2.1, 25)	-0.9 (-1.8, 0.1)
Average chest pain at rest (0-10, higher=worse pain)			
Baseline	0.4 (1.4, 32)	0.7 (1.7, 33)	-0.3 (-0.9, 0.5)
24 hours post-surgery	3.9 (2.2, 33)	3.3 (3.3, 32)	0.6 (-0.8, 2.0)
48 hours post-surgery	3.9 (2.1, 31)	4.3 (2.8, 31)	-0.4 (-1.6, 0.9)
72 hours post-surgery	3.2 (25, 2.6)	4.8 (2.8, 25)	-1.6 (-3.2, -0.1)
Discharge	3.1 (2.2, 29)	3.2 (2.5, 32)	-0.1 (-1.3, 1.1)
3 months	1.3 (1.4, 25)	1.5 (2.2, 21)	-0.2 (-1.3, 0.9)
**6 months**	0.4 (0.7, 27)	1.2 (1.7, 25)	-0.8 (-1.6, -0.1)
Average chest pain after coughing (0-10, higher=worse pain)			
Baseline	0.7 (1.9, 32)	0.9 (1.9, 33)	-0.2 (-1.2, 0.7)
24 hours post-surgery	6.2 (2.4, 33)	4.7 (3.3, 32)	1.5 (0.04, 2.9)
48 hours post-surgery	6.4 (2.5, 31)	6.1 (2.6, 31)	0.3 (-1.0, 1.5)
72 hours post-surgery	6.3 (2.6, 25)	7.0 (2.5, 26)	-0.7 (-2.2, 0.7)
Discharge	5.8 (2.2, 29)	5.5 (2.8, 32)	0.4 (-0.9, 1.7)
3 months	2.0 (1.8, 25)	1.95 (2.7, 21)	0.05 (-1.4, 1.4)
**6 months**	1.5 (1.9, 27)	2.1 (2.5, 24)	-0.6 (-1.8, 0.7)
Average chest pain after/with moving (0-10, higher=worse pain)			
Baseline	0.6 (1.9, 32)	0.8 (1.8, 33)	-0.2 (-1.1, 0.7)
24 hours post-surgery	6.1 (2.4, 33)	4.2 (3.3, 32)	1.9 (0.4, 3.3)
48 hours post-surgery	5.9 (2.3, 32)	5.4 (3.0, 31)	0.5 (-0.8, 1.9)
72 hours post-surgery	5.0 (2.6, 24)	6.3 (2.6, 25)	-1.3 (-2.8, 0.2)
Discharge	4.9 (2.2, 31)	4.5 (2.7, 31)	0.4 (-0.9, 1.6)
3 months	1.9 (1.8, 24)	2.2 (3.0, 23)	-0.3 (-1.8, 1.1)
**6 months**	1.0 (1.6, 27)	2.0 (2.7, 26)	-1.0 (-2.2, 0.3)
Average chest pain after physiotherapy (0-10, higher=worse pain)			
Baseline	0.04 (0.1, 29)	0.2 (0.5, 28)	-0.16 (-1.1, 0.7)
24 hours post-surgery	5.4 (2.3, 31)	4.1 (3.2, 30)	1.3 (-0.2, 2.7)
48 hours post-surgery	5.6 (2.3, 32)	5.0 (2.8, 31)	0.6 (-0.8, 1.9)
72 hours post-surgery	4.9 (2.4, 24)	5.9 (2.8, 25)	-1.0 (-2.5, 0.5)
Discharge	4.8 (2.1, 30)	3.8 (2.6 ,30)	1.0 (-0.2, 2.3)
3 months	1.95 (2.0, 18)	2.0 (2.9, 19)	-0.05 (-1.7, 1.6)
**6 months**	1.1 (1.6, 21)	2.0 (2.9, 19)	-0.9 (-2.3, 0.7)
Worst chest pain overall (0-10, higher=worse pain)			
Baseline	0.7 (1.7, 33)	0.9 (2.1, 33)	-0.2 (-1.2, 0.7)
24 hours post-surgery	7.7 (2.0, 33)	6.0 (3.5, 32)	1.7 (0.3, 3.2)
48 hours post-surgery	8.2 (1.6, 32)	7.4 (2.5, 31)	0.8 (-0.3, 1.9)
72 hours post-surgery	7.5 (2.0, 24)	7.9 (2.1, 25)	-0.4 (-1.6, 0.7)
Discharge	7.2 (2.1, 31)	6.6 (2.9, 31)	0.6 (-0.7, 1.9)
3 months	3.5 (2.9, 24)	2.7 (3.4, 23)	0.8 (-1.1, 2.7)
**6 months**	1.9 (2.5, 27)	3.1 (3.4, 26)	-1.2 (-2.8, 0.5)
Worst chest pain at rest (0-10, higher=worse pain)			
Baseline	0.3 (1.2, 33)	0.7 (1.7, 33)	-0.4 (-1.1, 0.4)
24 hours post-surgery	5.7 (2.8, 32)	4.8 (3.6, 32)	0.9 (-0.7, 2.5)
48 hours post-surgery	5.5 (2.7, 32)	5.4 (3.1, 31)	0.1 (-1.4, 1.6)
72 hours post-surgery	4.6 (2.8, 24)	6.1 (3.0, 25)	-1.5 (-3.2, 0.1)
Discharge	4.3 (2.5, 31)	4.7 (2.7, 31)	-0.4 (-1.7, 0.9)
3 months	2.2 (2.3, 24)	1.9 (2.7, 23)	0.3 (-1.2, 1.7)

For peer review only - http://bmjopen.bmj.com/site/about/guidelines.xhtml

**6 months**	1.0 (1.6, 27)	1.9 (2.7, 26)	-0.9 (-2.1, 0.3)
Worst chest pain after coughing (0-10, higher=worse pain)			
Baseline	0.6 (1.6, 33)	1.0 (2.2, 33)	-0.4 (-1.4, 0.6)
24 hours post-surgery	7.2 (1.9, 33)	5.5 (3.4, 32)	1.7 (0.3, 3.1)
48 hours post-surgery	7.6 (2.4, 32)	7.0 (2.5, 31)	0.6 (-0.7, 1.7)
72 hours post-surgery	7.2 (2.5, 25)	7.8 (2.1, 26)	-0.6 (-1.9, 0.7)
Discharge	6.7 (2.3, 30)	5.9 (2.9, 32)	0.8 (-0.5, 2.1)
3 months	2.9 (2.5, 25)	2.8 (3.1, 22)	0.1 (-1.7, 1.7)
**6 months**	1.8 (2.2, 28)	2.5 (2.9, 26)	-0.6 (-2.1, 0.8)
Worst chest pain after/with moving (0-10, higher=worse pain)			
Baseline	0.5 (1.5, 33)	0.9 (1.9, 33)	-0.4 (-1.2, 0.5)
24 hours post-surgery	7.2 (2.1, 33)	5.4 (3.6, 32)	1.8 (0.3, 3.3)
48 hours post-surgery	7.3 (1.9, 32)	6.5 (2.6, 31)	0.8 (-0.3, 2.1)
72 hours post-surgery	6.3 (2.3, 25)	7.0 (2.6, 26)	-0.7 (-2.1, 0.7)
Discharge	6.4 (2.3, 30)	5.8 (2.7, 32)	0.6 (-0.6, 1.9)
3 months	2.3 (1.8, 25)	2.5 (2.8, 22)	-0.2 (-1.6, 1.2)
**6 months**	1.3 (1.8, 28)	2.5 (3.2, 26)	-1.1 (-2.6, 0.4)
Worst chest pain after physiotherapy (0-10, higher=worse pain)			
Baseline	0.1 (0.2, 30)	0.2 (0.6, 29)	-0.1 (-0.4, 0.1)
24 hours post-surgery	6.2 (2.3, 31)	5.0 (3.5, 30)	1.2 (-0.4, 2.7)
48 hours post-surgery	6.5 (2.6, 31)	6.0 (2.8, 31)	0.6 (-0.8, 1.9)
72 hours post-surgery	5.9 (2.6, 25)	6.5 (2.6, 26)	-0.6 (-2.1, 0.8)
Discharge	6.0 (2.3, 29)	4.8 (2.8, 31)	1.2 (-0.1, 2.5)
3 months	2.6 (1.8, 25)	1.9 (2.7, 17)	0.7 (-0.9, 2.3)
**6 months**	1.2 (1.7, 22)	1.8 (2.8, 18)	-0.6 (-2.2, 1.0)

[1] scores<0 indicate less pain with PVB

For peer review only

Table S5: Complications, short-term mortality and length of stay summary

	Day 1		Day 2		Day 3		Between day 3 and discharge/final assessment	
	PVB	TEB	PVB	TEB	PVB	TEB	PVB	TEB
Arrhythmias	1/34 (3)	2/33 (6)	2/32 (6)	4/32 (13)	1/26 (4)	1/27 (4)	1/34 (3)	3/32 (9)
Systolic blood pressure (<90mmHg)	2/35 (6)	8/33 (24)	2/33 (6)	2/32 (6)	2/27 (7)	2/27 (7)	0/34 (-)	0/32 (-)
Low Respiratory rate (<10/minute)	1/35 (3)	0/33 (-)	0/33 (-)	0/32 (-)	0/27 (-)	0/27 (-)	0/34 (-)	0/32 (-)
Drowsiness	6/34 (18)	4/33 (12)	3/32 (9)	2/32 (6)	0/26 (-)	2/27 (7)	0/34 (-)	1/32 (3)
Nausea & vomiting	8/35 (23)	11/33 (33)	4/33 (12)	4/32 (13)	2/26 (8)	3/27 (11)	5/34 (15)	5/32 (16)
Urinary retention	0/35 (-)	0/33 (-)	1/33 (3)	0/32 (-)	0/27 (-)	0/27 (-)	1/34 (3)	1/32 (3)
Itching	0/34	1/33 (3)	0/32	1/32 (3)	0/26	0/27	0/32	1/32 (3)
High block (above T4)	0/35 (-)	0/33 (-)	0/33 (-)	0/32 (-)	0/27 (-)	0/27 (-)	0/34 (-)	0/32 (-)
Post-dural puncture headache	0/35 (-)	0/33 (-)	0/33 (-)	0/32 (-)	0/27 (-)	0/27 (-)	1/34 (3)	0/32 (-)
Post-op pulmonary complications (PPC)	2/21 (10)	1/19 (5)	2/19 (10)	1/18 (6)	1/15 (7)	1/15 (7)	0/19 (-)	0/19 (-)
Other	20/34 (59)	13/33 (39)	6/32 (19)	7/31 (23)	4/26 (15)	6/27 (22)	3/34 (9)	9/32 (28)
**Level 2 admission**	0/35 (-)	2/33 (6)	-	-	-	-	0/34 (-)	1/32 (3)
If yes, cardiovascular support	-	2/2 (100)	-	-	-	-	-	-
If yes, respiratory support	-	2/2 (100)	-	-	-	-	-	-
If yes, organ support	-	-	-	-	-	-	-	1/1 (100)
If yes, total length of stay *(Median [IQR], n)*		9 [6, 12]						6 [-]
**Level 3 admission**	1/35 (3)	1/32 (3)						
If yes, cardiovascular support	1/1 (100)	1/1 (100)	-	-	-	-	-	-
If yes, respiratory support	1/1 (100)	1/1 (100)	-	-	-	-	-	-
If yes, renal support	0/1 (-)	0/1 (-)	-	-	-	-	-	-
If yes, total length of stay *(Median [IQR], n)*	1 [-]	5 [-]	-	-	-	-	-	-
Discharge with flutter bag	-	-	-	-	-	-	5/34 (15)	4/33 (12)
Mortality	-	-	-	-	-	-	1/35 (3)	1/33 (3)

Frequency (%) presented (unless otherwise stated)

For peer review only

Table S6: Patient satisfaction and blinding questions results summary

		PVB	TEB
Satisfaction with pain therapy after surgery			
Discharge (n=31 for PVB, n=32 for TEB)	Very satisfied	18 (58)	20 (63)
	Satisfied	10 (33)	9 (28)
	Dissatisfied	2 (6)	2 (6)
	Very dissatisfied	1 (3)	1 (3)
3 months (n=26 for PVB, n=23 for TEB)	Very satisfied	13 (50)	9 (39)
	Satisfied	13 (50)	8 (35)
	Dissatisfied	0 (-)	6 (26)
	Very dissatisfied	0 (-)	0 (-)
6 months (n=26 for PVB, n=25 for TEB)	Very satisfied	14 (54)	16 (64)
	Satisfied	11 (42)	6 (24)
	Dissatisfied	0 (-)	2 (8)
	Very dissatisfied	1 (4)	1 (4)
Satisfaction with care provided by hospital			
Discharge (n=31 for PVB, n=32 for TEB)	Very satisfied	22 (71)	22 (69)
	Satisfied	9 (29)	8 (25)
	Dissatisfied	0 (-)	2 (6)
	Very dissatisfied	0 (-)	0 (-)
3 months (n=26 for PVB, n=23 for TEB)	Very satisfied	15 (58)	12 (52)
	Satisfied	11 (42)	9 (39)
	Dissatisfied	0 (-)	2 (9)
	Very dissatisfied	0 (-)	0 (-)
6 months (n=26 for PVB, n=26 for TEB)	Very satisfied	19 (73)	15 (58)
	Satisfied	5 (19)	9 (35)
	Dissatisfied	2 (8)	2 (8)
	Very dissatisfied	0 (-)	0 (-)
Do you know which technique you received?			
Discharge (n=31 for PVB, n=32 for TEB)	TEB	1 (3)	19 (60)
	PVB	8 (26)	3 (9)
	Don't know	22 (71)	10 (31)
3 months (n=26 for PVB, n=22 for TEB)	TEB	2 (8)	12 (55)
	PVB	2 (8)	0 (-)
	Don't know	22 (84)	10 (45)
6 months (n=26 for PVB, n=25 for TEB)	TEB	2 (8)	13 (52)
	PVB	5 (19)	1 (4)
	Don't know	19 (73)	11 (44)

Frequency (%) presented (unless otherwise stated)

For peer review only - http://bmjopen.bmj.com/site/about/guidelines.xhtml

CONSORT STATEMENT – PILOT STUDY

Section/topic and item No	Standard checklist item	Extension for pilot trials	Page No where item is reported
Title and abstract			
1a	Identification as a randomised trial in the title	Identification as a pilot or feasibility randomised trial in the title	1
1b	Structured summary of trial design, methods, results, and conclusions (for specific guidance see CONSORT for abstracts)	Structured summary of pilot trial design, methods, results, and conclusions (for specific guidance see CONSORT abstract extension for pilot trials)	2
Introduction			
Background and objectives:			
2a	Scientific background and explanation of rationale	Scientific background and explanation of rationale for future definitive trial, and reasons for randomised pilot trial	4
2b	Specific objectives or hypotheses	Specific objectives or research questions for pilot trial	4
Methods			
Trial design:			
3a	Description of trial design (such as parallel, factorial) including allocation ratio	Description of pilot trial design (such as parallel, factorial) including allocation ratio	4
3b	Important changes to methods after trial commencement (such as eligibility criteria), with reasons	Important changes to methods after pilot trial commencement (such as eligibility criteria), with reasons	N/A

For peer review only

Participants:			
4a	Eligibility criteria for participants		5
4b	Settings and locations where the data were collected		4
4c		How participants were identified and consented	7
Interventions:			
5	The interventions for each group with sufficient details to allow replication, including how and when they were actually administered		5-6
Outcomes:			
6a	Completely defined prespecified primary and secondary outcome measures, including how and when they were assessed	Completely defined prespecified assessments or measurements to address each pilot trial objective specified in 2b, including how and when they were assessed	6
6b	Any changes to trial outcomes after the trial commenced, with reasons	Any changes to pilot trial assessments or measurements after the pilot trial commenced, with reasons	N/A
6c		If applicable, prespecified criteria used to judge whether, or how, to proceed with future definitive trial	6
Sample size:			
7a	How sample size was determined	Rationale for numbers in the pilot trial	6
7b	When applicable, explanation of any interim analyses and stopping guidelines		N/A
Randomisation:			
Sequence generation:			

For peer review only

8a	Method used to generate the random allocation sequence		5
8b	Type of randomisation; details of any restriction (such as blocking and block size)	Type of randomisation(s); details of any restriction (such as blocking and block size)	5
Allocation concealment mechanism:			
9	Mechanism used to implement the random allocation sequence (such as sequentially numbered containers), describing any steps taken to conceal the sequence until interventions were assigned		5
Implementation:			
10	Who generated the random allocation sequence, enrolled participants, and assigned participants to interventions		5
Blinding:			
11a	If done, who was blinded after assignment to interventions (eg, participants, care providers, those assessing outcomes) and how		6
11b	If relevant, description of the similarity of interventions		6
Analytical methods:			
12a	Statistical methods used to compare	Methods used to address each pilot trial objective whether	7

For peer review only

	groups for primary and secondary outcomes	qualitative or quantitative	
12b	Methods for additional analyses, such as subgroup analyses and adjusted analyses	Not applicable	N/A
Results			
Participant flow (a diagram is strongly recommended):			
13a	For each group, the numbers of participants who were randomly assigned, received intended treatment, and were analysed for the primary outcome	For each group, the numbers of participants who were approached and/or assessed for eligibility, randomly assigned, received intended treatment, and were assessed for each objective	Figure 1
13b	For each group, losses and exclusions after randomisation, together with reasons		Figure 1
Recruitment:			
14a	Dates defining the periods of recruitment and follow-up		8
14b	Why the trial ended or was stopped	Why the pilot trial ended or was stopped	N/A
Baseline data:			
15	A table showing baseline demographic and clinical characteristics for each group		Table 1
Numbers analysed:			

For peer review only

16	For each group, number of participants (denominator) included in each analysis and whether the analysis was by original assigned groups	For each objective, number of participants (denominator) included in each analysis. If relevant, these numbers should be by randomised group	Table 2
Outcomes and estimation:			
17a	For each primary and secondary outcome, results for each group, and the estimated effect size and its precision (such as 95% confidence interval)	For each objective, results including expressions of uncertainty (such as 95% confidence interval) for any estimates. If relevant, these results should be by randomised group	Table 3, 4
17b	For binary outcomes, presentation of both absolute and relative effect sizes is recommended	Not applicable	N/A
Ancillary analyses:			
18	Results of any other analyses performed, including subgroup analyses and adjusted analyses, distinguishing prespecified from exploratory	Results of any other analyses performed that could be used to inform the future definitive trial	N/A
Harms:			
19	All important harms or unintended effects in each group (for specific guidance see CONSORT for harms)		14
19a		If relevant, other important unintended consequences	N/A
Discussion			
Limitations:			

For peer review only

20	Trial limitations, addressing sources of potential bias, imprecision, and, if relevant, multiplicity of analyses	Pilot trial limitations, addressing sources of potential bias and remaining uncertainty about feasibility	14-17
Generalisability:			
21	Generalisability (external validity, applicability) of the trial findings	Generalisability (applicability) of pilot trial methods and findings to future definitive trial and other studies	14-17
Interpretation:			
22	Interpretation consistent with results, balancing benefits and harms, and considering other relevant evidence	Interpretation consistent with pilot trial objectives and findings, balancing potential benefits and harms, and considering other relevant evidence	14-15
22a		Implications for progression from pilot to future definitive trial, including any proposed amendments	15-17
Other information			
Registration:			
23	Registration number and name of trial registry	Registration number for pilot trial and name of trial registry	2
Protocol:			
24	Where the full trial protocol can be accessed, if available	Where the pilot trial protocol can be accessed, if available	N/A
Funding:			
25	Sources of funding and other support (such as supply of drugs), role of funders		17
26		Ethical approval or approval by research review committee, confirmed with reference number	5

For peer review only

BMJ Open

A RANDOMISED CONTROLLED TRIAL TO INVESTIGATE THE EFFECTIVENESS OF THORACIC EPIDURAL AND PARAVERTEBRAL BLOCKADE IN REDUCING CHRONIC POST-THORACOTOMY PAIN (TOPIC) – A PILOT STUDY TO ASSESS FEASIBILITY OF A LARGE MULTI-CENTRE TRIAL

Journal:	*BMJ Open*
Manuscript ID	bmjopen-2018-023679.R3
Article Type:	Research
Date Submitted by the Author:	25-Jan-2019

Complete List of Authors:	Yeung, Joyce ; University of Warwick, Warwick Medical School; Heart of England NHS Foundation Trust, Department of Anaesthesia<br>Middleton, Lee; University of Birmingham, School of Health and Population Sciences<br>Tryposkiadis, Kostas; University of Birmingham, Birmingham Clinical Trials Unit<br>Kerr, Amy; Heart of England NHS Foundation Trust, Department of Thoracic Surgery<br>Daniels, Jane; University of Nottingham, Nottingham Clinical Trials Unit<br>Naidu, Babu; University of Birmingham, Institute of Inflammation and Ageing; Heart of England NHS Foundation Trust, Department of Thoracic Surgery<br>Melody, Teresa; Heart of England NHS Foundation Trust, Academic Department of Anaesthesia, Critical Care, Pain and Resuscitation<br>Goebel, Andreas; University of Liverpool, Institute of Translational Medicine<br>Wilson, Matthew; University of Sheffield, Health Services Research<br>Kumar, Sajith; Heart of England NHS Foundation Trust, Department of Anaesthesia<br>Szentgyorgyi, Lajos; University Hospital of South Manchester NHS Foundation Trust, Department of Anaesthesia<br>Flanagan, Sarah; University of Birmingham, Institute of Applied Health Research<br>Shah, Rajesh; University Hospital of South Manchester NHS Foundation Trust, Department of Thoracic Surgery<br>Worrall, Antony; Heart of England NHS Foundation Trust<br>Gao, Fang; University of Birmingham, Institute of Inflammation and Ageing, College of Medical and Dental Sciences; Heart of England NHS Foundation Trust, Academic Department of Anaesthesia, Critical Care, Pain and Resuscitation

**Primary Subject Heading**:	Anaesthesia
Secondary Subject Heading:	Surgery
Keywords:	Pain management < ANAESTHETICS, Adult anaesthesia <

	ANAESTHETICS, Thoracic surgery < SURGERY, Clinical trials < THERAPEUTICS

A RANDOMISED CONTROLLED TRIAL TO INVESTIGATE THE EFFECTIVENESS OF THORACIC EPIDURAL AND PARAVERTEBRAL BLOCKADE IN REDUCING CHRONIC POST-THORACOTOMY PAIN (TOPIC) – A PILOT STUDY TO ASSESS FEASIBILITY OF A LARGE MULTI-CENTRE TRIAL

Authors:

J Yeung[1,2,], **L Middleton**[3], **K Tryposkiadis**[3], **A Kerr**[1], **J Daniels**[4], **B Naidu**[1,5], **T Melody**[1], **A Goebel**[6], **M Wilson**[7], **S Kumar**[1], **L Szentgyorgyi**[8], **S Flanagan**[9], **R Shah**[8], **A Worrall**[1], **and F Gao**[1,5], On behalf of TOPIC Investigator Group

[1] Department of Anaesthesia, Heart of England NHS Foundation Trust, Birmingham, United Kingdom

[2] Warwick Medical School, University of Warwick, Coventry, United Kingdom

[3] Birmingham Clinical Trials Unit, University of Birmingham, Birmingham, United Kingdom

[4]Nottingham Clinical Trials Unit, University of Nottingham, Nottingham, United Kingdom

[5]Institute of Inflammation and Ageing, University of Birmingham, Birmingham, United Kingdom

[6]University of Liverpool and Walton Centre NHS Foundation Trust, Liverpool, United Kingdom

[7]School of Health and Related Research, University of Sheffield, Sheffield, United Kingdom

[8]Manchester University NHS Foundation Trust, Manchester, United Kingdom

[9]Institute of Applied Health Research, University of Birmingham, Birmingham, United Kingdom

Corresponding author: Fang Gao

E-mail: f.gaosmith@bham.ac.uk Tel: 01214147483

Institute of Inflammation and Ageing, College of Medical and Dental Sciences, University of Birmingham, Birmingham B15 2TT, United Kingdom

Word Count 3618

For peer review only

ABSTRACT

Objectives: Thoracotomy is considered one of the most painful surgical procedures. The incidence of chronic post-thoracotomy pain (CPTP) is up to 50%. Paravertebral blockade (PVB) may be superior to thoracic epidural blockade (TEB) in preventing CPTP. The specific objective of this pilot study was to assess the feasibility of conducting a larger trial to determine whether PVB at thoracotomy is more effective in reducing CPTP compared to TEB.

Design: A randomised, parallel, external pilot study was conducted to assess whether a large randomised trial of TEB and PVB with CPTP as the primary outcome is feasible.

Setting: Two adult thoracic centres in the UK

Participants: All adult patients admitted for elective open thoracotomy. Participants were excluded if they were ASA physical status IV or V; or if there is contraindication to local anaesthetics; infection near the proposed puncture site; coagulation/thoracic spine disorders; required chest wall resection or emergency thoracic surgery or had a previous thoracotomy.

Results: All patients presenting for thoracotomy were screened over a 12-month period with 194 found to be eligible. Of these, 69 (36%) were randomised (95%CI: 29%-42%). Discounting five participants who died, 54 of 64 participants (84%) returned questionnaire booklets at six months. The number of participants indicating at least a moderate level of chest pain at six months was lower with PVB but with high levels of uncertainty (RR: 0.7; 95%CI: 0.3-1.7 for worst pain; RR: 0.3; 95%CI: 0.0-2.8 for average). There were no safety concerns.

Conclusions: A large multicentre RCT of PVB versus TEB is feasible as it is possible to randomise and follow up participants with high fidelity. Pain scores were lower on average with PVB compared to TEB but a much larger trial is required to confirm this reliably.

Trial Registration: ISRCTN45041624

For peer review only

Strengths and limitations of this study

- This randomised, external, pilot trial had pre-planned feasibility thresholds to assess whether a large trial is feasible.
- Selected two sites represented the national thoracic and anaesthetic cohort, providing best representation of full trial national recruitment
- Consultant anaesthetists in both participating sites had adequate training and appropriate assessment for the competence of each technique.
- The study was not designed and powered to be large or long enough to provide convincing evidence to support the use of either TEB or PVB as intraoperative anaesthetic technique, but provides evidence that a larger, substantive trial is feasible.

For peer review only

BACKGROUND

An estimated 7200 thoracotomies (surgical incision into the chest wall) are performed annually in the UK, most commonly to treat lung cancer.[1] Thoracotomy is considered one of the most painful surgical procedures due to tissue, muscle and nerve damage from the incision and wound retraction. Intercostal nerve injury can result in a high risk of persistent pain for months after surgery.[2-4] The incidence of chronic post-thoracotomy pain (CPTP; defined as pain that recurs or persists at least two months following the surgery)[5] is thought to be as high as 50%.[6]

Two main analgesic techniques are commonly used for perioperative pain control during thoracotomy. Thoracic epidural blockade (TEB) blocks nerves that supply the chest with local anaesthetic bilaterally, at spinal cord level. It acts by reducing onward transmission of painful nerve signals but may not abolish them completely.[7,8] Paravertebral blockade (PVB), involves injecting local anaesthetic into the paravertebral space, which contains spinal nerves (and sometimes even extension of the dura), white and grey rami communicantes, the sympathetic chain and intercostal vessels, on the side of surgery.[9] There are studies which describe the spread of PVB injections anteriorly across the heads and necks of the ribs to the spaces above and below; medially through an intervertebral foramen or spread laterally in the intercostal plane.[9] However compared to TEB, PVB has the potential to completely block painful nerve signals from reaching the spinal cord.[9,10] This total blockade of nerve signals could remove the stimulus for ‘central sensitisation’ which underpins the formation of chronic pain pathways. PVB could be uniquely effective in preventing long-term pain[11], and there is evidence from a recent trial of two techniques in breast surgery to support this premise.[12] With limited current evidence to support the most effective choice of anaesthetic technique in preventing CPTP, current UK practice varies greatly.[13] A recent Cochrane Review recommended that a high quality randomised controlled trial (RCT) to compare TEB and PVB with the primary outcome of chronic pain is urgently needed.[14]

The overall aim of this research is to determine whether PVB at thoracotomy is more effective in reducing CPTP compared to TEB. To answer this question a large, multi-centre RCT is required. The specific aim of this pilot study was to assess the feasibility of conducting a larger trial and to enhance the likelihood of its success by developing the necessary structure and processes that a large trial would need. Objectives included: an assessment of the effectiveness of the patient identification and screening process; identification of reasons for failure to randomise; development of educational and training materials for surgeons and anaesthetists; evaluation of robustness of in-hospital data collection processes; assessment of trial processes including impact on participants and staff.

For peer review only

METHODS

We conducted a randomised, parallel group, external pilot trial of TEB versus PVB for perioperative pain control during thoracotomy. Recruitment took place over 12 months in two adult thoracic centres: Heartlands Hospital, Birmingham and University Hospital South Manchester (Wythenshawe). This study was approved by NHS Research Ethics Committee (NRES Committee East Midlands - Derby, REC number 14/EM/1280) and was prospectively registered (ISRCTN45041624).

Population and inclusion/exclusion criteria

Eligible participants were all adults admitted for an elective open thoracotomy. Participants were excluded if they had any of the following: ASA (classification of fitness) physical status IV or V (as these patients are unlikely to present for elective surgery due to their physical condition); contraindication to local anaesthetics; infection near the proposed puncture site; coagulation abnormalities (in accordance with Association of Anaesthetists in Great Britain and Ireland guidance 2013) ; thoracic spine disorders; required chest wall resection or emergency thoracic surgery or had a previous thoracotomy (scarring due to previous surgery can limit the effectiveness of paravertebral block and these patients may already have existing chronic pain).

Study conduct

All patients thought to fulfil the eligibility criteria were approached with study information at their pre-operative assessment clinic. They were provided with a Patient Information Sheet and given the opportunity to consider participation. Once eligibility was confirmed and written informed consent was obtained, randomisation was performed prior to surgery using a web-based central randomisation system (via Birmingham Clinical Trials Unit) to allocate patients to either TEB or PVB in a 1:1 ratio. Minimisation was used to achieve balance between sex, age (<65 years or ≥65 years), thoracotomy for lung cancer resection or for other indication and study site. Research nurses at each site was responsible for randomisation and assigned group allocation was revealed to responsible anaesthetist when patients arrived at the operating theatre. An Oversight Committee was formed to provide independent guidance to the Trial Management Committee and to review accruing safety information during the period of recruitment. Public and patient involvement was integral to the study throughout.

Interventions

Interventions were delivered by experienced thoracic anaesthetists, trained and deemed competent in both anaesthetic techniques. Participants were not told specifically about their allocated intervention. It was not possible to blind anaesthetists, surgeons or nursing staff due to the nature of the interventions. PVB was performed using three single injections pre-incision with the patient awake

For peer review only

or asleep, at the levels of T3-4, 5-6 and 7-8 with 15ml 0.25% levobupivicaine/bupivacaine, using a landmark technique at each level. The PVB catheter was then placed at T5 under direct vision by a surgeon during surgery. A loading dose of 10ml 0.25% levobupivicaine/bupivacaine was administered before chest closure, followed by infusion of 0.125% levobupivicaine/bupivacaine 0.1-0.25ml kg^{-1} hr^{-1} until operation end. TEB was inserted pre-incision with the patient awake or asleep, with a catheter inserted at the spinal level supplying the skin at the incision site (normally T5-6), a test dose of 3ml of 0.5% bupivacaine, and a loading dose of 0.25% levobupivicaine/bupivacaine 0.1ml kg^{-1} with up to 3mg of diamorphine. This was followed by infusion of 0.125% levobupivicaine/bupivacaine with 2µg ml^{-1} fentanyl at 0.1-0.25 ml kg^{-1} hr^{-1}. Further information on delivery and training on the interventions is available in the study protocol.[15] Identical dressings and pumps were used for both interventions to maintain group allocation.

Pilot outcomes

The following outcomes and targets were set a-priori as being indicative that a larger trial would be feasible to conduct.[15] These were: i) patient recruitment rate (the proportion of eligible patients randomised) at least 25%; ii) screening rate (the proportion of all patients listed for elective thoracotomy that were screened for eligibility and recorded on a screening log) at least 90%; iii) clinicians' willingness to recruit: national survey of consultant thoracic anaesthetist indicating the number willing to participate in a larger study at least 70%; iv) data completion – number of anaesthetic/peri-operative forms completed at least 90% and follow-up questionnaires completed at six months at least 80%. The study was given a 12-month recruitment period, after which screening would stop.

Clinical and participant reported outcome measures

Baseline data (including participant completed questions as indicated below) were recorded at the pre-operative assessment appointment. Intraoperative (anaesthetic technique and use), post-operative and discharge data (analgesic use, pain scores on days 1-3, acute complications, mortality and length of stay) were collected by the study team. Three and six-month post-randomisation follow-up was conducted by postal questionnaire. This included: Visual Analogue Scales (VAS)[16] for worst and average chest pain (overall, at rest, after coughing, after moving, after physiotherapy; with higher score - maximum 10 - indicating higher levels of pain, as marked along a 10cm line), Brief Pain Inventory (BPI)[17], Neuropathic Pain Scale (NPS)[18], generic health-related quality of life (EQ-5D-5L)[19], Hospital Anxiety and Depression Scale (HADS)[20] and assessment of satisfaction and participant blinding. Serious adverse events were collected throughout. Patient outcome data was collected by research team member who was blinded to the assigned group.

For peer review only

Statistics

We expected to recruit between 50-75 participants over a 12-month recruitment period, depending on the number found to be eligible. We estimated that there would be approximately 500 open elective thoracotomies from the two sites, of which 60% would potentially be eligible (300). Using our own target criteria of 25% recruited would yield 75 participants. This number would be sufficient to measure the recruitment rate to uncertainty width up to approximately 10%.

Feasibility outcomes were considered with simple summary statistics (proportions and percentages), with uncertainty estimates provided by 95% confidence intervals. Clinical and participant reported outcome measures were analysed with point estimates (RR or mean difference) and 95% confidence intervals, adjusting for the minimisation variables. They were not subject to hypothesis testing as the size of the sample would not allow reliable assessment of efficacy.[21] Participants were considered in the groups they were randomised to regardless of compliance (intention-to-treat).

Qualitative assessment and survey of practice

In addition to the clinical assessment, semi-structured qualitative interviews were undertaken with 18 patients at 6-8 weeks discharge to gauge the impact of the trial on their hospital care and eight clinical staff (four anaesthetists, one surgeon and three members of the research team) to aid insight into trial related processes. Patient who were recruited to the trial were asked if they would be willing to be interviewed about their experiences of being in the trial. Those who consented to be interviewed were contacted by the qualitative researcher to arrange a telephone interview. The name and contact details of staff who had been involved in the trail and were willing to be interviewed were provided to the qualitative researcher and an interview was arranged. The patient and staff telephone interviews were audio-recorded and field notes were be taken to in order to capture the interviewer's thoughts subsequent to completing each interview. Audio-recordings were transcribed in full by an experienced team of professional transcribers, with transcripts subsequently proof read against the recordings by the researcher. Anonymised transcripts were analysed thematically concurrently with data collection in order to allow emerging findings to be included in subsequent interviews.[22] The resulting codes and themes were refined and consistency and variation across the interviews was explored. Once this process was completed, the resulting themes generated from the data were summarised. The main themes for patient interviews included the acceptability of the trial; motivations for being involved in the trial; experiences of participating in the trial – for example, did patients feel well-informed about what involvement would entail; thoughts about the questionnaires used to assess levels of pain. The main themes from the staff interviews included experiences of patient identification and screening; experiences of the randomisation process; and reflections on the

For peer review only

trial in general terms –what processes went well and were there any areas for improvement. An electronic national survey of anaesthetists on willingness to participate in a future trial was also conducted via the Association for Cardiothoracic Anaesthesia and Critical Care membership in 26 UK thoracic units.

Patient involvement

Our study question in chronic pain post thoracotomy is in the top ranked research priorities voted by patient 'What can we do to stop patients developing chronic pain after surgery?' The development of study protocol was designed in conjunction with our patient partners who also participated as a member of trial management group and trial steering committee. Our patient partners reviewed study design, paying attention to acceptability of randomisation analgesic techniques in perioperative setting and burden of study participation. Study results including plan for future study has been disseminated within local and national patient research ambassador groups.

RESULTS

Participants and follow-up

564 patients were screened for eligibility from July 2015 to July 2016 - 100% of those listed for elective thoracotomy - with 194 patients found to be eligible (Figure 1). The most common reasons for ineligibility were: undergoing video-assisted thoracoscopic surgery (174/370, 47%); participation in another study which did not allow co-enrolment (59/370, 16%); previous thoracotomy (31/370, 8%). Of the 194 patients, 69 (36%, 95%CI: 29% to 42%) were ultimately randomised. Of the others, the most common reasons for non-randomisation were: anaesthetists trained to perform PVB not available (24/125, 19%); did not want to be randomised (15/125, 12%); patient had preference for PVB (15/125, 12%). Details of randomised participants are given in Table 1. The minimisation algorithm provided appropriate balance for the balancing factors; the average age of participants was 66 years with the vast majority were having a lung cancer resection operation (60/69, 87%). Seven participants (10%) reported previous problems with (unspecified) chronic pain.

At six months post-randomisation, 54 of 63 (86%) questionnaire booklets were returned, discounting 5 participants who died during the follow-up period and one participant who withdrew from the study. Of these, only one did not complete their VAS questions.

For peer review only

Table 1. Baseline characteristics of patients

		PVB (n=35)	TEB (n=34)
Age >=65, years[1]		21 (60)	20 (59)
Age, years	Mean (SD)	66.0 (10.6)	65.9 (7.9)
Gender=male[1]		19 (54)	20 (59)
Centre[1]	Birmingham Heartlands	23 (66)	26 (76)
	University Hospital South Manchester	12 (34)	8 (24)
Reason for thoracotomy=lung cancer resection[1]		30 (86)	30 (88)
ASA[2] physical status	1: Normal healthy	0 (-)	2 (6)
	2: Mild systemic disease	13 (37)	10 (30)
	3: Severe systemic disease	22 (63)	21 (64)
	Missing	-	1
ECOG[3] performance status	0: Normal activity	20 (57)	19 (58)
	1: Symptomatic but nearly fully ambulatory	14 (40)	14 (42)
	2: Symptomatic but bed <50% daytime	1 (3)	0 (-)
	3: Symptomatic bed>50% daytime	0 (-)	0 (-)
	4: Unable to get out of bed	0 (-)	0 (-)
	Missing	-	1
Dyspnoea	0: None	17 (48)	17 (52)
	1: Slight	16 (46)	13 (39)
	2: Moderate	2 (6)	1 (3)
	3: Moderately severe	0 (-)	2 (6)
	4: Severe	0 (-)	0 (-)
	5: Very severe	0 (-)	0 (-)
	Missing	-	1
Smoking status	Never smoked	7 (20)	6 (18)
	Stopped>1 year	19 (54)	18 (55)
	Stopped>6 weeks	3 (9)	3 (9)
	Stopped<6 weeks	1 (3)	4 (12)
	Current smoker	5 (14)	2 (6)
	Missing	-	1
Smoking-pack years	Median [IQR]	37 [20, 49]	37 [20, 50]
	Missing	-	1
Alcohol units per week	Median [IQR]	3 [0, 13.5]	0 [0, 9.7]
	Missing	-	1
Previous medical history=yes	COPD	7 (20)	6 (18)
	Ischaemic heart disease	4 (11)	3 (9)
	Congestive cardiac failure	0 (-)	1 (3)
	Hypertension	18 (51)	15 (45)
	Diabetes diet controlled	0 (-)	3 (9)
	Diabetes oral medication	6 (17)	3 (9)
	Diabetes insulin controlled	2 (6)	1 (3)
	Renal failure	1 (3)	1 (3)
	Previous stroke	0 (-)	0 (-)
	Hyperthyroidism	0 (-)	0 (-)
	Hypothyroidism	5 (14)	2 (6)
	Other cancer	12 (34)	7 (21)
	Chronic pain	2 (6)	5 (15)
	Missing	-	1
BMI, kg/m^2	Mean (SD)	28.4 (5.8)	27.7 (5.9)
	Missing	-	1

Frequency (%) presented (unless otherwise stated)

[1] minimisation variable; [2] American Society of Anesthesiologists; [3] Eastern Cooperative Oncology Group

For peer review only

Analgesic technique

Of the 69 randomised, 67 (97%) received their randomised intervention. One participant allocated PVB received TEB as the pleura was removed and the surgeon was unable to place the PVB. Another participant was not considered fit for surgery and was withdrawn from the study. Discounting this participant, all anaesthetic technique and intra-operative forms were completed. A summary of the anaesthetic techniques is given in Table 2; a summary of the operative details and other analgesics provided to participants up to discharge is provided in the Appendix (Tables S1, S2).

Table 2. Anaesthetic technique summary

		PVB (n=35)	TEB (n=33)[1]
Difficulty in insertion		4 (12)[2]	14 (42)
Failure to deliver technique		1 (3)	0 (-)
Intraoperative use			
Preoperative bolus given		26 (74)	25 (76)
Bolus drug given through catheter		14 (40)	24 (73)
Infusion given through catheter		23 (66)	21 (64)
Post-operative settings			
Local anaesthetic was Bupivacaine (as opposed to Levobupivacaine)		32 (94)[2]	19 (58)
Concentration	0.1%	20 (59)[2]	11 (33)
	0.125%	4 (12)[2]	22 (67)
	0.25%	10 (29)[2]	0 (-)
Fentanyl	None	32 (91)	3 (9)
	2 mcg/ml	0 (-)	0 (-)
	4 mcg/ml	3 (9)	30 (91)
Starting rate, ml/hr	Median [IQR]	20 [10, 20][2]	6 [6, 8]
Prescribed range, ml/hr	Median [IQR]	20 [15, 20][2]	12 [8, 15]

Frequency (%) presented (unless otherwise stated)

[1] one participant received neither technique as they did not have an operation; [2] one response missing (n=34)

For peer review only

Patient reported outcomes

Levels of pain in the three days post-operation appeared similar in both groups (Appendix Table S3). The number of patients indicating at least a moderate level of average or worst chest pain (based on commonly used threshold of >3 or ≥4[23, 24]) was lower with PVB compared to TEB at six months, but with expected high levels of uncertainty given the limited size of sample (Table 3; full results given in Appendix Table S4). Results from other questionnaire responses had a lot of uncertainty (Table 4).

Table 3. Incidence of significant (>3 or >=4) or severe pain (>=7) from Visual Analogue Scale Scores

	PVB n (%)	TEB n (%)	RR (95% CI) [1]
Average chest pain overall>3			
3 months	5/25 (20.0)	6/21 (28.6)	0.7 (0.2, 2.0)
**6 months**	2/27 (7.4)	5/25 (20.0)	0.4 (0.1, 1.7)
Average chest pain overall>=4			
3 months	5/25 (20.0)	4/21 (19.0)	1.1 (0.3, 3.4)
**6 months**	1/27 (3.7)	3/25 (12.0)	0.3 (0.03, 2.8)
Average chest pain overall>=7			
3 months	0/25 (-)	2/21 (10.0)	- [2]
**6 months**	0/27 (-)	1/25 (4.0)	- [2]
Worst chest pain overall>3			
3 months	9/24 (37.5)	7/23 (30.4)	1.2 (0.6, 2.8)
**6 months**	7/27 (25.9)	11/26 (42.3)	0.6 (0.3, 1.3)
Worst chest pain overall>=4			
3 months	8/24 (33.3)	7/23 (30.4)	1.1 (0.5, 2.5)
**6 months**	7/27 (25.9)	9/26 (34.6)	0.75 (0.3, 1.7)
Worst chest pain overall>=7			
3 months	6/24 (25.0)	5/23 (21.7)	1.2 (0.4, 3.3)
**6 months**	2/27 (7.4)	5/26 (19.2)	0.4 (0.1, 1.8)

[1] RR<~~0~~ 1 indicate less incidence with PVB; [2] unable to estimate

For peer review only

Table 4. Results of patient reported outcomes

	PVB mean score (SD, n)	TEB mean score (SD, n)	Difference between group means s (95% CI)
**Brief pain inventory (BPI) results summary[1]**			
Pain severity score (0-10, higher=worse pain)			
Baseline	0.9 (1.7, 34)	1.3 (2.0, 32)	-0.4 (-1.3, 0.5)
24 hours post-surgery	5.3 (1.7, 33)	3.9 (2.7, 32)	1.4 (0.3, 2.5)
48 hours post-surgery	5.2 (1.7, 30)	5.1 (2.5, 31)	0.1 (-0.9, 1.2)
72 hours post-surgery	4.9 (1.7, 23)	5.6 (2.0, 25)	-0.7 (-1.8, 0.4)
Discharge	4.0 (1.4, 31)	4.1 (2.4, 32)	-0.1 (-1.1, 0.9)
3 months	1.6 (1.7, 22)	2.5 (2.5, 21)	0.9 (-2.2, 0.4)
**6 months**	1.3 (1.8, 25)	2.3 (2.6, 23)	-1.0 (-2.3, 0.3)
Pain interference score (0-10, higher=worse pain)			
Baseline	0.6 (1.3, 34)	1.5 (2.2, 32)	-0.9 (-1.8, -0.1)
24 hours post-surgery	5.8 (2.1, 30)	5.4 (2.9, 28)	0.4 (-0.8, 1.8)
48 hours post-surgery	6.0 (2.0, 30)	5.8 (2.2, 27)	0.2 (-0.9, 1.3)
72 hours post-surgery	5.4 (2.3, 21)	5.8 (2.6, 23)	-0.4 (-1.9, 1.1)
Discharge	4.7 (2.0, 23)	4.3 (2.5, 29)	0.4 (-0.9, 1.7)
3 months	1.1 (1.4, 24)	2.8 (3.0, 22)	-1.7 (-3.1, -0.3)
**6 months**	1.6 (2.2, 27)	1.9 (2.5, 25)	-0.3 (-1.6, 1.0)
**Neuropathic pain scale (NPS) results summary[1]**			
How intense does your pain feel? (0-10, higher=worse pain)			
Baseline	1.7 (2.8, 33)	1.8 (2.8, 33)	-0.1 (-1.5, 1.3)
Discharge	5.8 (2.8, 31)	5.4 (3.8, 31)	0.4 (-1.3, 2.1)
3 months	2.2 (2.4, 25)	3.6 (3.8, 21)	-1.4 (-3.4, 0.5)
**6 months**	2.2 (2.7, 27)	2.7 (3.2, 26)	-0.5 (-2.2, 1.1)
How sharp does your pain feel? (0-10, higher=worse pain)			
Baseline	1.1 (2.8, 33)	1.6 (3.4, 32)	-0.5 (-2.1, 1.1)
Discharge	5.5 (3.2, 31)	5.4 (4.0, 31)	0.1 (-1.7, 1.9)
3 months	2.6 (3.0, 25)	3.2 (4.0, 21)	-0.6 (-2.8, 1.5)
**6 months**	3.0 (3.3, 27)	2.4 (3.3, 26)	0.6 (-1.3, 2.4)
How hot does your pain feel? (0-10, higher=worse pain)			
Baseline	1.0 (2.6, 33)	1.3 (2.7, 32)	-0.3 (-1.6, 1.0)
Discharge	3.6 (3.4, 31)	2.4 (2.9, 31)	1.2 (-0.4, 2.8)
3 months	1.6 (2.8, 25)	2.5 (3.3, 21)	-0.9 (-2.7, 1.0)
**6 months**	1.2 (2.0, 28)	1.6 (2.9, 26)	-0.4 (-1.7, 1.0)
How dull does your pain feel? (0-10, higher=worse pain)			
Baseline	1.8 (2.9, 33)	1.5 (2.5, 33)	0.3 (-1.0, 1.7)
Discharge	2.9 (2.7, 30)	3.9 (2.8, 30)	-1.0 (-2.4, 0.5)
3 months	2.6 (2.1, 25)	3.3 (3.3, 21)	-0.7 (-2.4, 0.9)
**6 months**	2.1 (2.8, 28)	2.3 (2.8, 26)	-0.2 (-1.8, 1.2)
How cold does your pain feel? (0-10, higher=worse pain)			
Baseline	0.3 (1.6, 33)	0.4 (1.7, 32)	-0.1 (-0.9, 0.7)
Discharge	1.5 (2.5, 30)	1.0 (2.2, 31)	0.5 (-0.7, 1.7)
3 months	0.2 (0.5, 25)	1.5 (2.9, 21)	-1.3 (-2.7, -0.0)
**6 months**	0.3 (0.9, 28)	0.7 (1.6, 26)	-0.4 (-1.1, 0.3)
How sensitive is your pain? (0-10, higher=worse pain)			
Baseline	0.5 (1.9, 33)	1.1 (2.5, 32)	-0.6 (-1.7, 0.5)
Discharge	2.3 (2.9, 30)	2.8 (2.9, 32)	-0.5 (-1.9, 1.0)
3 months	2.2 (3.0, 25)	2.1 (2.7, 23)	0.1 (-1.6, 1.7)
**6 months**	1.6 (2.0, 28)	2.5 (3.1, 26)	-0.9 (-2.4, 0.5)
How itchy is your pain? (0-10, higher=worse pain)			
Baseline	0 (0, 33)	0.8 (2.0, 32)	-0.8 (-1.5, -0.0)
Discharge	2.2 (2.8, 30)	2.6 (3.3, 31)	-0.4 (-2.0, 1.2)
3 months	1.2 (2.5, 25)	1.8 (2.6, 22)	-0.6 (-2.1, 0.9)
**6 months**	0.4 (0.7, 28)	2.1 (2.6, 25)	-1.7 (-2.8, -0.6)
How unpleasant is your pain? (0-10, higher=worse pain)			
Baseline	1.9 (2.9, 31)	2.1 (3.3, 33)	-0.2 (-1.7, 1.4)
Discharge	6.2 (3.0, 30)	5.6 (3.3, 30)	0.6 (-1.1, 2.2)
3 months	2.8 (2.6, 24)	4.0 (3.9, 21)	-1.2 (-3.3, 0.8)

For peer review only

**6 months**	2.4 (2.7, 28)	3.0 (3.2, 24)	0.6 (-2.3, 1.0)
How intense is your deep pain? (0-10, higher=worse pain)			
Baseline	1.3 (2.4, 32)	2.4 (3.7, 33)	-1.1 (-2.6, 0.5)
Discharge	6.2 (2.8, 31)	5.5 (3.4, 31)	0.7 (-0.9, 2.3)
3 months	3.0 (2.8, 23)	4.1 (3.7, 21)	-1.1 (-3.1, 0.9)
**6 months**	2.8 (3.0, 28)	2.5 (2.9, 25)	0.3 (-1.4, 1.9)
How intense is your surface pain? (0-10, higher=worse pain)			
Baseline	0.8 (2.1, 31)	0.7 (1.5, 32)	0.1 (-0.9, 1.0)
Discharge	3.9 (3.4, 30)	3.8 (3.3, 32)	0.1 (-1.7, 1.7)
3 months	1.9 (2.2, 24)	3.5 (3.6, 21)	-1.6 (-3.3, 0.2)
**6 months**	1.2 (1.5, 27)	2.7 (3.2, 24)	-1.5 (-2.9, -0.1)
**EQ-5D-DL results summary[2]**			
EQ-5D index score (-0.59=worst outcome, 1.0=best outcome)			
Baseline	0.75 (0.22, 35)	0.77 (0.20, 33)	-0.02 (-0.12, 0.08)
Discharge	0.49 (0.23, 27)	0.49 (0.23, 30)	-0.00 (-0.12, 0.12)
3 months	0.73 (0.17, 25)	0.65 (0.23, 23)	0.08 (-0.04, 0.20)
**6 months**	0.72 (0.23, 25)	0.69 (0.22, 25)	0.03 (-0.09, 0.16)
EQ-5D thermometer (0-100, higher=better)			
Baseline	76.6 (16.3, 34)	74.3 (17.8, 33)	2.3 (-5.9, 10.7)
Discharge	60.4 (22.7, 26)	60.2 (20.7, 30)	0.2 (-11.5, 11.9)
3 months	73.2 (21.2, 25)	67.5 (18.3, 23)	5.7 (-5.8, 17.2)
**6 months**	78.0 (14.0, 25)	64.4 (20.4, 25)	13.6 (3.6, 23.6)
**Hospital anxiety and depression index score (HADS) results summary[3]**			
Depression index score (0-21, lower=better)			
Baseline	2.9 (2.9, 33)	3.0 (2.6, 32)	-0.1 (-1.4, 1.3)
Discharge	4.3 (3.4, 28)	6.0 (5.0, 28)	-1.7 (-4.0, 0.5)
3 months	3.6 (2.7, 25)	5.5 (4.0, 21)	-1.9 (-4.1, 0.1)
**6 months**	4.5 (3.5, 24)	6.0 (4.6, 25)	-1.5 (-3.9, 0.8)
Anxiety index score (0-21, 0-lower=better)			
Baseline	6.3 (5.0, 34)	6.5 (4.4, 30)	-0.2 (-2.6, 2.1)
Discharge	5.0 (4.4, 29)	7.0 (5.3, 28)	-2.0 (-4.6, 0.6)
3 months	5.2 (4.3, 25)	6.1 (4.8, 19)	-0.9 (-3.7, 1.9)
**6 months**	5.7 (3.6, 24)	7.3 (5.0, 25)	-1.6 (-4.1, 0.9)

[1] scores<0 indicate less pain with PVB

[2] scores>0 indicate better QoL with PVB

[3] scores<0 indicate less anxiety/depression with PVB

For peer review only

Safety

No concerns were expressed by the independent Oversight Committee who met twice during the recruitment period to review study progress and safety data. Complications and short-term mortality data appeared similar in both groups (Appendix Table S5). There were five deaths; only one was considered to be a serious adverse event, this was aspiration pneumonitis and was not considered related to intervention (PVB). One other participant experienced a serious adverse event in the TEB group; this participant was diagnosed with acute kidney injury in the post-operative period.

Assessment of participant blinding

19/32 (60%) patients in the TEB group correctly identified the anaesthetic technique they had been allocated at discharge; the figure for PVB was 8/31 (26%); this reduced to 13/24 (52%) in the TEB group at 6 months and 5/26 (19%) in the PVB group (Appendix Table S6).

National survey of anaesthetists

Forty-three responses were returned. Of these, 27 anaesthetists indicated that they would be willing to randomise in a future study (63%). Five (12%) indicating that they would 'possibly' be willing to randomise with reasons of concern given as lack of skill and ward structure. The remaining 11 (26%) were unwilling.

Qualitative interviews

Interviewed participants had a positive experience, reporting that they felt well informed by trial staff. The consent process was felt to be well undertaken and patients felt reassured by the explanations of randomisation. A substantial number of participants reported that the study questionnaires were long and repetitive. Interviewed staff felt recruitment was successful although there were some challenges when the role was undertaken by non-clinical staff. There was a feeling that some of the data collection tools were repetitive. Clinical staff were supportive of the trial; they found that the trial processes, randomisation and procedures were very straightforward. The guidance and teaching provided for anaesthetists to get them up to speed for performing PVB was very positively received and it was considered a relatively simple procedure to learn.

DISCUSSION

Our four key indicators for feasibility were met and we have shown that a large multicentre randomised controlled trial of PVB versus TEB for peri-operative pain control with an objective of assessing long-term post-thoracotomy chronic pain is feasible. It is possible to randomise and follow

For peer review only

up patients with high fidelity over six months. In the qualitative assessment we carried out, participants and staff were largely happy with study processes. In particular, the consent, randomisation and assessment methods were considered appropriate. As hypothesised, VAS pain scores were lower with PVB compared with TEB at six months on average but with high levels of uncertainty. This would need to be investigated further in a much more substantial trial.

Whilst the evidence on short-term outcomes, e.g. minor complications and analgesic efficacy, point to PVB being at least as effective as TEB[14], current evidence on whether PVB is more effective than TEB in reducing rates of chronic pain is limited. A recent systematic review failed to attain sufficient data to compare the impact of TEB and PVB techniques on CPTP. [14] The incidence of CPTP was very poorly reported and studies did not describe how data on chronic pain was measured or collected. The lack of evidence highlights an area in need of further research. We are not aware of any current or planned randomised trials with CPTP as an outcome that may change these findings since this systematic review was published. Incidence of CPTP was slightly lower in our study - between 30-34% - than previously quoted at 49%[6], although we need to acknowledge that our figure is associated with a reasonably large amount of uncertainty given the small size of sample. A likely contributing factor to this discrepancy is the considerable and widely acknowledged variation in how pain is measured[25], as it is dependent on the measurement instrument and threshold chosen amongst other factors.[23]

This feasibility work has allowed us to fine tune study processes ahead of a larger trial. One likely modification to the future study protocol is to revise the length of the follow-up questionnaires, it was considered by participants that their length was too long and repetitive in content. Shortened and simplified follow-up questionnaire would also potentially helping to improve follow-up figures further, as lengthy questionnaires are a known predictor of participant drop-out from trials.[26]

Inclusion and exclusion criteria appeared appropriate and do not appear to need to be changed. The vast majority of ineligible participants were those scheduled to receive video-assisted thoracoscopic surgery (VATS). Whilst this procedure is becoming more popular[1], the comparison in question is not an appropriate one as TEB is not routinely used to provide pain relief for in this type of surgery. The VATS approach also does not require any rib spreading, resulting in a less invasive procedure, and whilst chronic pain is still an issue after VATS it is not considered to be as great as following open thoracotomy.[27] Unfortunately, whilst VATS is becoming more common, there will still be a substantial proportion of patients that will always be ineligible for this operation because of, for example, inability to achieve complete resection in more advanced pulmonary lung cancers. Lung cancer remains the most common cancer worldwide.[28,29]

For peer review only

It was apparent from the assessment of the ability to blind the participant to the analgesic technique that complete blinding is unlikely to be possible. Substantially more participants appeared to be able to determine they had had an epidural blockade than the paravertebral technique. Attempts to completely blind participants in any future study are likely to make processes prohibitively complex and expensive due to innate differences in the mode of delivery and action in the two analgesic techniques. This could include sham insertion techniques to disguise the randomised intervention. The question is whether patient blinding is likely to be an important potential cause of detection bias, unduly affecting the results of any future trial. The proposed primary outcome of pain rating is subjective in nature and this is a theoretical possibility. However, we believe that there is no reason to suspect that recipients of the randomised interventions have strong pre-conceptions with regard to the relative effectiveness of each analgesic technique. Furthermore, the primary outcome would be collected via questionnaires administered by post or phone, at a time remote from the original operative procedure, which are likely to be resilient to the effects of imperfect concealment.

Findings from the survey of practice were encouraging, although whilst we set a target of 70% of thoracic anaesthetists willing to randomise to a future trial as being indicative of success, we found 63% willing and 12% 'possibly willing'. To achieve 70%, we would require solutions for those anaesthetists expressing uncertainty. The major reasons given were lack of skill and ward structure. These hurdles do not appear insurmountable if funding for further training and extra nurse support could be provided; something a large funding body may be willing to provide. A successful trial would also require full engagement with anaesthetic and thoracic surgical trainees' network to maximise recruitment.

We have reported the study to randomised pilot study standards as recommended by CONSORT extension for pilot study.[30] Also as recommended,[31] we set objectives a priori to determine whether the pilot study would demonstrate feasibility of a larger trial. Since this study was not a definitive trial, we have been careful not to over-interpret the findings. Hence at this stage, we cannot expect that the outcomes of this pilot study to be directly translated into clinical care, as the study was not large enough to be able to detect small to moderate realistic-sized difference in rates of chronic pain, nor was the scope wide enough in terms of the number of centres involved to return a generalisable result.

Nevertheless, this pilot study is an important precursor to a larger, substantive, trial and provides invaluable information that will help to ensure its success. A definitive study - instigated by the authors of this manuscript - has now been funded by the NIHR Health Technology Assessment Programme and is expected to start recruiting in 2018 from 20 UK thoracic centres. The design of this study has been partially informed by the results of this pilot. The primary outcome will be incidence of chronic pain at six months attained from visual analogue scale responses similar to that used here; other outcomes

For peer review only

will follow IMMPACT recommendations.[32] A total sample size of 1026 participants will give 90% power (p=0.05) to detect a reduction in incidence of CPTP of 30% with TEB (a similar event rate to that noted in this pilot study) down to 20% with PVB. The rate of recruitment noted in this study has been factored into the number of sites and length of the recruitment period required; we estimate access to a pool of 5000 participants over a two and a half year recruitment period and need to recruit a fifth of these. The design will allow a definitive answer this important question.

AUTHOR'S CONTRIBUTION

FG, JY, JPD and MW conceived the topic. FG, JY, JPD, LJM and AG designed the study with the input from SK, LS, BN, MW, TM and AW. FG oversaw the running of the trial and all the authors contributed to the ongoing management of the trial. AK, RS, BN collected data for the trial. KT performed the statistical analysis. SF evaluated the qualitative data. The manuscript was drafted by LJM, JY and FG with contributions from the other authors. All the authors contributed to the interpretation of the output and revised and reviewed the paper; they are the guarantors. The Birmingham Clinical Trials Unit undertook the randomisation and data management and monitoring. The authors had full access to all the data from the study. The authors vouch for the accuracy and completeness of the data and analyses. All authors read and approved the final manuscript.

ACKNOWLEDGEMENTS

We thank the trial participants; members of the trial Oversight Committee: Tom Treasure (Chair), Alistair Macfie (independent anaesthetist), Mahmoud Loubani (independent surgeon), Lucy Bradshaw (independent statistician), Alistair Murray (patient and public representative); the Clinical Research Ambassador Group at Heart of England NHS Foundation Trust; Birmingham Clinical Trials Unit staff: Matt Hill (database programmer) and Hannah Watson (data manager).

COMPETING INTERESTS

All authors declare that they have no competing interests.

FUNDING

The study was funded by a grant from the National Institute for Health Research for Patient Benefit (RFPB grant number PB-PG-0213-30126). The funder had no role in study design, data collection, interpretation or analysis, or in writing the report for publication. S F is funded by the National Institute of Health Research (NIHR) Collaboration for Leadership in Applied Health Research and Care West Midlands (CLAHRCWM).

For peer review only

SPONSORSHIP

The study sponsor was the Heart of England NHS Foundation Trust. The sponsor had no role in study design, data collection, interpretation or analysis, or in writing the report for publication.

DATA SHARING STATEMENT

All available study data can be obtained from the corresponding author.

For peer review only

References

1. The Society for Cardiothoracic Surgery in Great Britain and Ireland. SCTS 2014-2015 Thoracic Registry Data. 2015.

2. Hughes R, Gao F. Pain control for thoracotomy. *Continuing Education in Anaesthesia, Critical Care and Pain.* 2005;5(2):56-60.

3. Soto RG, Fu ES. Acute pain management for patients undergoing thoracotomy. *Ann Thorac Surg.* 2003;75(4):1349-1357.

4. Kavanagh BP, Katz J, Sandler AN. Pain control after thoracic surgery. A review of current techniques. *Anesthesiology.* 1994;81(3):737-759.

5. International Association for the Study of Pain. Classification of chronic pain. Descriptions of chronic pain syndromes and definitions of pain terms. Prepared by the International Association for the Study of Pain, Subcommittee on Taxonomy. *Pain Supplement.* 1986;3:S1-226.

6. Bayman EO, Brennan TJ. Incidence and severity of chronic pain at 3 and 6 months after thoracotomy: meta-analysis. *The Journal of Pain.* 2014;15(9):887-897.

7. Dahl JB, Rosenberg J, Lund C, Kehlet H. Effect of thoracic epidural bupivacaine 0.75% on somatosensory evoked potentials after dermatomal stimulation. *Reg Anesth.* 1990;15(2):73-75.

8. Lund C, Hansen OB, Mogensen T, Kehlet H. Effect of thoracic epidural bupivacaine on somatosensory evoked potentials after dermatomal stimulation. *Anesth Analg.*1987;66(8):731-734.

9. Richardson J, Lönnqvist PA. Thoracic paravertebral block. *Br J Anaesth.* 1998;81(2):230-238.

10. Cook E, Downs C. Analgesia After Thoracotomy - The Role of the Extrapleural Paravertebral Catheter. *Australian Anaesthesia.* 2005:103-117.

11. Richardson J, Jones J, Atkinson R. The effect of thoracic paravertebral blockade on intercostal somatosensory evoked potentials. *Anesthesia & Analgesia.* 1998;87(2):373-376.

12. Karmakar MK, Samy W, Li JW, et al. Thoracic paravertebral block and its effects on chronic pain and health-related quality of life after modified radical mastectomy. *Reg Anesth Pain Med.* 2014;39(4):289-298.

13. Turner J, Yeung J. Analgesic technique in thoracic surgery: a survey of UK ACTACC members. Unpublished data.2016.

14. Yeung JH, Gates S, Naidu BV, Wilson MJ, Gao Smith F. Paravertebral block versus thoracic epidural for patients undergoing thoracotomy. *Cochrane Database Syst Rev.* 2016;2:CD009121.

15. Yeung J, Melody T, Kerr A, Naidu B, Middleton L, Tryposkiadis K, Daniels J, Gao F. Randomised controlled pilot study to investigate the effectiveness of thoracic epidural and paravertebral blockade in reducing chronic post-thoracotomy pain: TOPIC feasibility study protocol. BMJ Open. 2016 Dec 1;6(12):e012735. doi: 10.1136/bmjopen-2016-012735.

For peer review only - http://bmjopen.bmj.com/site/about/guidelines.xhtml

16. Jensen MP, Karoly P, Braver S. The measurement of clinical pain intensity: a comparison of six methods. Pain. 1986 Oct;27(1):117-26.

17. Tan G, Jensen MP, Thornby JI, et al. Validation of the Brief Pain Inventory for chronic nonmalignant pain. J Pain 2004;5:133–7.

18. Galer BS, Jensen MP. Development and preliminary validation of a pain measure specific to neuropathic pain: the Neuropathic Pain Scale. Neurology 1997;48:332–8.

19. Herdman M, Gudex C, Lloyd A, et al. Development and preliminary testing of the new five-level version of EQ-5D (EQ-5D-5L). Qual Life Res 2011;20:1727–36.

20. Zigmond AS, Snaith RP. The hospital anxiety and depression scale. Acta Psychiatr Scand. 1983;67:361–70.

21. Moore CG, Carter RE, Nietert PJ, Stewart PW. Recommendations for planning pilot studies in clinical and translational research. Clin Transl Sci. 2011 Oct;4(5):332-7. doi: 10.1111/j.1752-8062.2011.00347.x.

22. Pope C, Ziebland S, Mays N. Qualitative research in health care. Analysing qualitative data. *BMJ* 2000;320(7227):114-6.

23. Jensen MP, Chen C, Brugger AM. Interpretation of visual analog scale ratings and change scores: a reanalysis of two clinical trials of postoperative pain. J Pain. 2003;4:407-14.

24. Palos GR, Mendoza TR, Mobley GM, Cantor SB, Cleeland CS. Asking the community about cutpoints used to describe mild, moderate, and severe pain. J Pain. 2006 Jan;7(1):49-56.

25. Litcher-Kelly L, Martino SA, Broderick JE, Stone AA. A systematic review of measures used to assess chronic musculoskeletal pain in clinical and randomized controlled clinical trials. The journal of pain: official journal of the American Pain Society. 2007;8(12):906-913.doi:10.1016/j.jpain.2007.06.009.

26. Edwards P, Roberts I, Sandercock P, Frost C. Follow-up by mail in clinical trials: does questionnaire length matter? Control Clin Trials. 2004 Feb;25(1):31-52

27. Bendixen M, Jørgensen OD, Kronborg C, Andersen C, Licht PB. Postoperative pain and quality of life after lobectomy via video-assisted thoracoscopic surgery or anterolateral thoracotomy for early stage lung cancer: a randomised controlled trial. Lancet Oncol. 2016 Jun;17(6):836-844. doi: 10.1016/S1470-2045(16)00173-X. Epub 2016 May 6.

28. Ferlay J, Soerjomataram I, Ervik M, Dikshit R, Eser S, Mathers C et al. GLOBOCAN 2012 v1.0, Cancer Incidence and Mortality Worldwide: IARC Cancer Base No. 11.

29. Stewart BW, Wild CP, editors. World cancer report 2014. Lyon: International Agency for Research on Cancer; 2014.

30. Eldridge SM, Chan CL, Campbell MJ, Bond CM, Hopewell S, Thabane L, Lancaster GA. CONSORT 2010 statement: extension to randomised pilot and feasibility trials. BMJ. 2016 Oct 24;355:i5239. doi: 10.1136/bmj.i5239.

For peer review only

31. Thabane L, Ma J, Chu R, Cheng J, Ismaila A, Rios LP, Robson R, Thabane M, Giangregorio L, Goldsmith CH. A tutorial on pilot studies: the what, why and how. BMC Medical Research Methodology201010:1 https://doi.org/10.1186/1471-2288-10-1.

32. Dworkin RH, Turk DC, Farrar JT, Haythornthwaite JA, Jensen MP, Katz NP et al. Core outcome measures for chronic pain clinical trials: IMMPACT recommendations. Pain. 2005; 113:9-19.

For peer review only

LEGEND FOR FIGURE 1

Figure 1. Flow chart of patients through the study

For peer review only

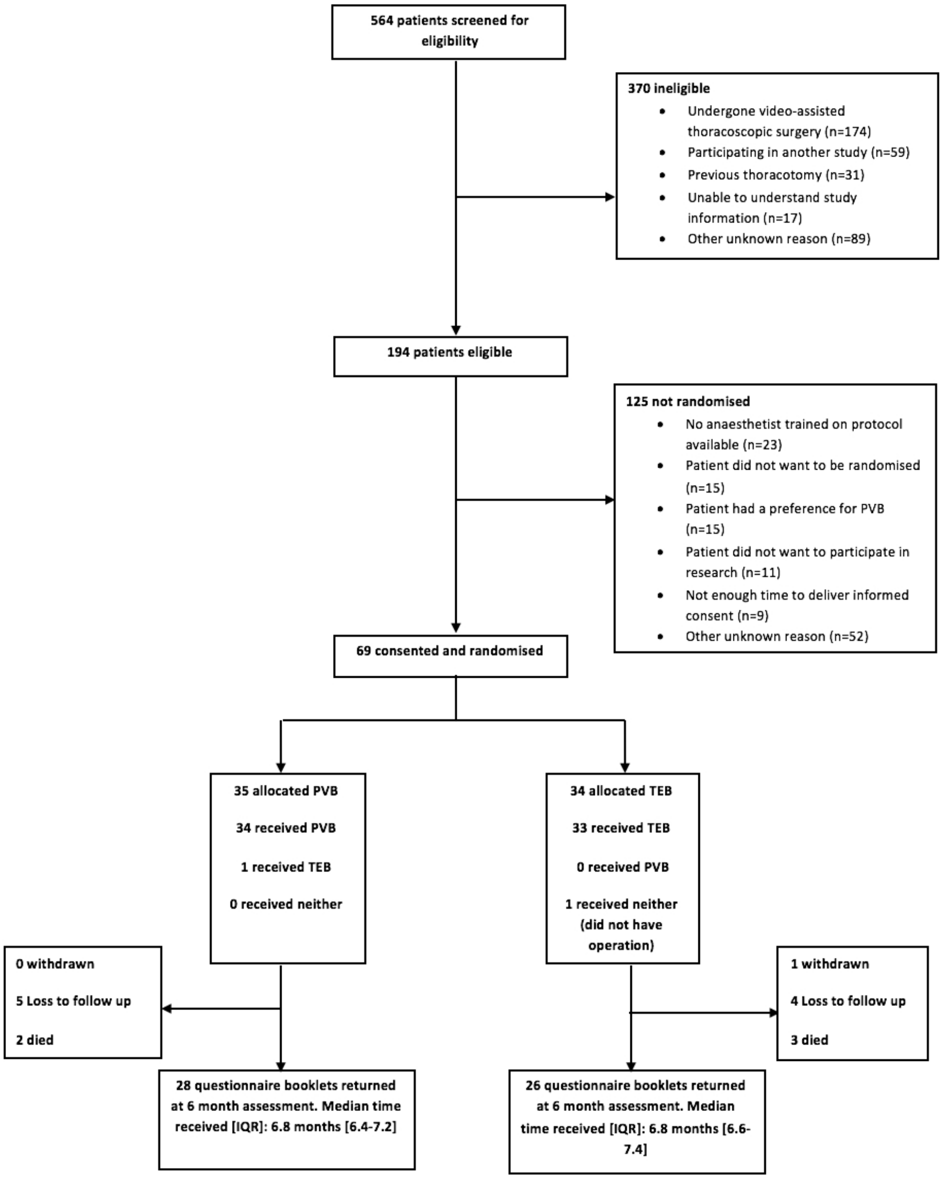

Figure 1

79x98mm (300 x 300 DPI)

TOPIC PILOT STUDY SUPPLEMENTARY APPENDIX

Table S1: Intraoperative data summary 2

Table S2: Analgesic use summary 3

Table S3: Post-operative (in hospital) pain scores summary 4

Table S4: Comprehensive summary of Visual Analogue Scale (VAS) scores 5

Table S5: Complications, short-term mortality and length of stay summary 7

Table S6: Patient satisfaction and blinding questions results summary 8

For peer review only

Table S1: Intraoperative data summary

		PVB (n=35)	TEB (n=33)[1]
Vasopressor/inotrope used		30 (86)	27 (82)
Delivery route	Boluses	24 (80)	24 (89)
	Infusion	3 (10)	1 (4)
	Both	3 (10)	2 (7)
Overall fluid therapy and balance	Median [IQR]	1000 [1000, 2000]	1000 [1000, 1500]
Grade of surgeon was consultant (as opposed to trainee)		21 (60)	18 (55)
Side of operation was left (as opposed to right)		9 (26)	10 (30)
Type of operation	Lobectomy	18 (51)	18 (55)
	Pneumonectomy	3 (9)	2 (6)
	Segmentectomy	1 (3)	1 (3)
	Wedge	3 (9)	2 (6)
	Lung biopsy	1 (3)	0 (-)
	Wedge and Segmentectomy	0 (-)	1 (3)
	Lobectomy and Wedge	1 (3)	0 (-)
	Lobectomy and Other	2 (6)	2 (6)
	Other	6 (17)	7 (21)
Surgical approach	Anterolateral	4 (12)[2]	2 (6)
	Posterolateral	22 (65)[2]	23 (70)
	Muscle sparing	0 (-)[2]	1 (3)
	Mini-thoracotomy	1 (3)[2]	0 (-)
	Posterolateral and Muscle sparing	7 (21)[2]	6 (18)
	Mini-limited lateral	0 (-)[2]	1 (3)

Frequency (%) presented (unless otherwise stated)
[1] one participant received neither technique as they did not have an operation; [2] one response missing (n=34)

For peer review only

Table S2: Analgesic use summary

			In Hospital (post-surgery)							
	Intraoperative		Day 1		Day 2		Day 3		Between day 3 and discharge/final assessment	
**Drug used**	**PVB**	**TEB**	**PVB**	**TEB**	**PVB**	**TEB**	**PVB**	**TEB**	**PVB**	**TEB**
Paracetamol	29	29	1	-	-	-	-	-	-	-
Diclofenac	3	-	3	2	-	-	-	-	-	-
Remifentanil	-	-	6	5	2	-	-	-	-	-
Fentanyl	-	-	16	11	5	4	2	-	-	-
Morphine	2	-	6	1	14	3	3	2	2	-
Diamorphine	-	2	-	10	1	7	-	1	-	-
Gabapentin	-	-	-	-	-	-	-	1	-	-
Alfentanil	-	-	1	2	3	10	1	1	-	-
Bupivicaine	-	-	-	-	-	-	-	1	-	-
Levobupivicaine	-	-	-	-	-	1	-	-	-	-
Pregabalin	-	-	-	-	-	-	1	-	-	-
Ketorolac	-	-	-	-	-	2	3	2	2	-

Number of participants administered drug indicated (total number given PVB=35 and total number given TEB=33)

For peer review only

Table S3: Post-operative (in hospital) pain scores summary

	PVB mean score (SD, n)	TEB mean score (SD, n)	Difference between groups (95% CI) [1]
Pain score at rest (0-3, higher=worse pain)			
Day 1	0.4 (0.7, 34)	0.5 (1.0, 33)	-0.1 (-0.5, 0.3)
Day 2	0.5 (0.7, 33)	0.7 (1.0, 32)	-0.2 (-0.6, 0.3)
Day 3	0.2 (0.4, 27)	0.6 (0.7, 27)	-0.4 (-0.8, -0.1)
Pain score at movement (0-3, higher=worse pain)			
Day 1	0.9 (0.9, 34)	0.8 (1.0, 33)	0.1 (-0.3, 0.6)
Day 2	1.2 (0.8, 33)	1.3 (0.9, 32)	-0.1 (-0.5, 0.4)
Day 3	0.8 (0.9, 27)	1.0 (1.0, 27)	-0.2 (-0.8, 0.3)

[1] scores<0 indicate less pain with PVB

For peer review only

For peer review only - http://bmjopen.bmj.com/site/about/guidelines.xhtml

Table S4: Comprehensive summary of Visual Analogue Scale (VAS) scores

	PVB mean score (SD, n)	TEB mean score (SD, n)	Difference between groups (95% CI) [1]
Average chest pain overall (0-10, higher=worse pain)			
Baseline	0.6 (1.8, 32)	0.7 (1.7, 33)	-1.0 (-1.0, 0.8)
24 hours post-surgery	5.2 (2.5, 33)	3.7 (3.4, 32)	1.5 (-0.02, 2.9)
48 hours post-surgery	6.4 (1.8, 31)	5.5 (2.7, 31)	0.9 (-0.3, 2.1)
72 hours post-surgery	5.1 (25, 2.4)	6.1 (2.5, 26)	-1.0 (-2.4, 0.3)
Discharge	4.9 (1.9, 29)	4.3 (2.5, 32)	0.6 (-0.5, 1.7)
3 months	2.1 (1.8, 25)	2.0 (2.6, 21)	0.1 (-1.3, 1.4)
**6 months**	0.8 (1.2, 27)	1.7 (2.1, 25)	-0.9 (-1.8, 0.1)
Average chest pain at rest (0-10, higher=worse pain)			
Baseline	0.4 (1.4, 32)	0.7 (1.7, 33)	-0.3 (-0.9, 0.5)
24 hours post-surgery	3.9 (2.2, 33)	3.3 (3.3, 32)	0.6 (-0.8, 2.0)
48 hours post-surgery	3.9 (2.1, 31)	4.3 (2.8, 31)	-0.4 (-1.6, 0.9)
72 hours post-surgery	3.2 (25, 2.6)	4.8 (2.8, 25)	-1.6 (-3.2, -0.1)
Discharge	3.1 (2.2, 29)	3.2 (2.5, 32)	-0.1 (-1.3, 1.1)
3 months	1.3 (1.4, 25)	1.5 (2.2, 21)	-0.2 (-1.3, 0.9)
**6 months**	0.4 (0.7, 27)	1.2 (1.7, 25)	-0.8 (-1.6, -0.1)
Average chest pain after coughing (0-10, higher=worse pain)			
Baseline	0.7 (1.9, 32)	0.9 (1.9, 33)	-0.2 (-1.2, 0.7)
24 hours post-surgery	6.2 (2.4, 33)	4.7 (3.3, 32)	1.5 (0.04, 2.9)
48 hours post-surgery	6.4 (2.5, 31)	6.1 (2.6, 31)	0.3 (-1.0, 1.5)
72 hours post-surgery	6.3 (2.6, 25)	7.0 (2.5, 26)	-0.7 (-2.2, 0.7)
Discharge	5.8 (2.2, 29)	5.5 (2.8, 32)	0.4 (-0.9, 1.7)
3 months	2.0 (1.8, 25)	1.95 (2.7, 21)	0.05 (-1.4, 1.4)
**6 months**	1.5 (1.9, 27)	2.1 (2.5, 24)	-0.6 (-1.8, 0.7)
Average chest pain after/with moving (0-10, higher=worse pain)			
Baseline	0.6 (1.9, 32)	0.8 (1.8, 33)	-0.2 (-1.1, 0.7)
24 hours post-surgery	6.1 (2.4, 33)	4.2 (3.3, 32)	1.9 (0.4, 3.3)
48 hours post-surgery	5.9 (2.3, 32)	5.4 (3.0, 31)	0.5 (-0.8, 1.9)
72 hours post-surgery	5.0 (2.6, 24)	6.3 (2.6, 25)	-1.3 (-2.8, 0.2)
Discharge	4.9 (2.2, 31)	4.5 (2.7, 31)	0.4 (-0.9, 1.6)
3 months	1.9 (1.8, 24)	2.2 (3.0, 23)	-0.3 (-1.8, 1.1)
**6 months**	1.0 (1.6, 27)	2.0 (2.7, 26)	-1.0 (-2.2, 0.3)
Average chest pain after physiotherapy (0-10, higher=worse pain)			
Baseline	0.04 (0.1, 29)	0.2 (0.5, 28)	-0.16 (-1.1, 0.7)
24 hours post-surgery	5.4 (2.3, 31)	4.1 (3.2, 30)	1.3 (-0.2, 2.7)
48 hours post-surgery	5.6 (2.3, 32)	5.0 (2.8, 31)	0.6 (-0.8, 1.9)
72 hours post-surgery	4.9 (2.4, 24)	5.9 (2.8, 25)	-1.0 (-2.5, 0.5)
Discharge	4.8 (2.1, 30)	3.8 (2.6 ,30)	1.0 (-0.2, 2.3)
3 months	1.95 (2.0, 18)	2.0 (2.9, 19)	-0.05 (-1.7, 1.6)
**6 months**	1.1 (1.6, 21)	2.0 (2.9, 19)	-0.9 (-2.3, 0.7)
Worst chest pain overall (0-10, higher=worse pain)			
Baseline	0.7 (1.7, 33)	0.9 (2.1, 33)	-0.2 (-1.2, 0.7)
24 hours post-surgery	7.7 (2.0, 33)	6.0 (3.5, 32)	1.7 (0.3, 3.2)
48 hours post-surgery	8.2 (1.6, 32)	7.4 (2.5, 31)	0.8 (-0.3, 1.9)
72 hours post-surgery	7.5 (2.0, 24)	7.9 (2.1, 25)	-0.4 (-1.6, 0.7)
Discharge	7.2 (2.1, 31)	6.6 (2.9, 31)	0.6 (-0.7, 1.9)
3 months	3.5 (2.9, 24)	2.7 (3.4, 23)	0.8 (-1.1, 2.7)
**6 months**	1.9 (2.5, 27)	3.1 (3.4, 26)	-1.2 (-2.8, 0.5)
Worst chest pain at rest (0-10, higher=worse pain)			
Baseline	0.3 (1.2, 33)	0.7 (1.7, 33)	-0.4 (-1.1, 0.4)
24 hours post-surgery	5.7 (2.8, 32)	4.8 (3.6, 32)	0.9 (-0.7, 2.5)
48 hours post-surgery	5.5 (2.7, 32)	5.4 (3.1, 31)	0.1 (-1.4, 1.6)
72 hours post-surgery	4.6 (2.8, 24)	6.1 (3.0, 25)	-1.5 (-3.2, 0.1)
Discharge	4.3 (2.5, 31)	4.7 (2.7, 31)	-0.4 (-1.7, 0.9)
3 months	2.2 (2.3, 24)	1.9 (2.7, 23)	0.3 (-1.2, 1.7)

For peer review only - http://bmjopen.bmj.com/site/about/guidelines.xhtml

**6 months**	1.0 (1.6, 27)	1.9 (2.7, 26)	-0.9 (-2.1, 0.3)
Worst chest pain after coughing (0-10, higher=worse pain)			
Baseline	0.6 (1.6, 33)	1.0 (2.2, 33)	-0.4 (-1.4, 0.6)
24 hours post-surgery	7.2 (1.9, 33)	5.5 (3.4, 32)	1.7 (0.3, 3.1)
48 hours post-surgery	7.6 (2.4, 32)	7.0 (2.5, 31)	0.6 (-0.7, 1.7)
72 hours post-surgery	7.2 (2.5, 25)	7.8 (2.1, 26)	-0.6 (-1.9, 0.7)
Discharge	6.7 (2.3, 30)	5.9 (2.9, 32)	0.8 (-0.5, 2.1)
3 months	2.9 (2.5, 25)	2.8 (3.1, 22)	0.1 (-1.7, 1.7)
**6 months**	1.8 (2.2, 28)	2.5 (2.9, 26)	-0.6 (-2.1, 0.8)
Worst chest pain after/with moving (0-10, higher=worse pain)			
Baseline	0.5 (1.5, 33)	0.9 (1.9, 33)	-0.4 (-1.2, 0.5)
24 hours post-surgery	7.2 (2.1, 33)	5.4 (3.6, 32)	1.8 (0.3, 3.3)
48 hours post-surgery	7.3 (1.9, 32)	6.5 (2.6, 31)	0.8 (-0.3, 2.1)
72 hours post-surgery	6.3 (2.3, 25)	7.0 (2.6, 26)	-0.7 (-2.1, 0.7)
Discharge	6.4 (2.3, 30)	5.8 (2.7, 32)	0.6 (-0.6, 1.9)
3 months	2.3 (1.8, 25)	2.5 (2.8, 22)	-0.2 (-1.6, 1.2)
**6 months**	1.3 (1.8, 28)	2.5 (3.2, 26)	-1.1 (-2.6, 0.4)
Worst chest pain after physiotherapy (0-10, higher=worse pain)			
Baseline	0.1 (0.2, 30)	0.2 (0.6, 29)	-0.1 (-0.4, 0.1)
24 hours post-surgery	6.2 (2.3, 31)	5.0 (3.5, 30)	1.2 (-0.4, 2.7)
48 hours post-surgery	6.5 (2.6, 31)	6.0 (2.8, 31)	0.6 (-0.8, 1.9)
72 hours post-surgery	5.9 (2.6, 25)	6.5 (2.6, 26)	-0.6 (-2.1, 0.8)
Discharge	6.0 (2.3, 29)	4.8 (2.8, 31)	1.2 (-0.1, 2.5)
3 months	2.6 (1.8, 25)	1.9 (2.7, 17)	0.7 (-0.9, 2.3)
**6 months**	1.2 (1.7, 22)	1.8 (2.8, 18)	-0.6 (-2.2, 1.0)

[1] scores<0 indicate less pain with PVB

For peer review only

Table S5: Complications, short-term mortality and length of stay summary

	Day 1		Day 2		Day 3		Between day 3 and discharge/final assessment	
	PVB	TEB	PVB	TEB	PVB	TEB	PVB	TEB
Arrhythmias	1/34 (3)	2/33 (6)	2/32 (6)	4/32 (13)	1/26 (4)	1/27 (4)	1/34 (3)	3/32 (9)
Systolic blood pressure (<90mmHg)	2/35 (6)	8/33 (24)	2/33 (6)	2/32 (6)	2/27 (7)	2/27 (7)	0/34 (-)	0/32 (-)
Low Respiratory rate (<10/minute)	1/35 (3)	0/33 (-)	0/33 (-)	0/32 (-)	0/27 (-)	0/27 (-)	0/34 (-)	0/32 (-)
Drowsiness	6/34 (18)	4/33 (12)	3/32 (9)	2/32 (6)	0/26 (-)	2/27 (7)	0/34 (-)	1/32 (3)
Nausea & vomiting	8/35 (23)	11/33 (33)	4/33 (12)	4/32 (13)	2/26 (8)	3/27 (11)	5/34 (15)	5/32 (16)
Urinary retention	0/35 (-)	0/33 (-)	1/33 (3)	0/32 (-)	0/27 (-)	0/27 (-)	1/34 (3)	1/32 (3)
Itching	0/34	1/33 (3)	0/32	1/32 (3)	0/26	0/27	0/32	1/32 (3)
High block (above T4)	0/35 (-)	0/33 (-)	0/33 (-)	0/32 (-)	0/27 (-)	0/27 (-)	0/34 (-)	0/32 (-)
Post-dural puncture headache	0/35 (-)	0/33 (-)	0/33 (-)	0/32 (-)	0/27 (-)	0/27 (-)	1/34 (3)	0/32 (-)
Post-op pulmonary complications (PPC)	2/21 (10)	1/19 (5)	2/19 (10)	1/18 (6)	1/15 (7)	1/15 (7)	0/19 (-)	0/19 (-)
Other	20/34 (59)	13/33 (39)	6/32 (19)	7/31 (23)	4/26 (15)	6/27 (22)	3/34 (9)	9/32 (28)
**Level 2 admission**	0/35 (-)	2/33 (6)	-	-	-	-	0/34 (-)	1/32 (3)
If yes, cardiovascular support	-	2/2 (100)	-	-	-	-	-	-
If yes, respiratory support	-	2/2 (100)	-	-	-	-	-	-
If yes, organ support	-	-	-	-	-	-	-	1/1 (100)
If yes, total length of stay *(Median [IQR], n)*		9 [6, 12]						6 [-]
**Level 3 admission**	1/35 (3)	1/32 (3)						
If yes, cardiovascular support	1/1 (100)	1/1 (100)	-	-	-	-	-	-
If yes, respiratory support	1/1 (100)	1/1 (100)	-	-	-	-	-	-
If yes, renal support	0/1 (-)	0/1 (-)	-	-	-	-	-	-
If yes, total length of stay *(Median [IQR], n)*	1 [-]	5 [-]	-	-	-	-	-	-
Discharge with flutter bag	-	-	-	-	-	-	5/34 (15)	4/33 (12)
Mortality	-	-	-	-	-	-	1/35 (3)	1/33 (3)

Frequency (%) presented (unless otherwise stated)

For peer review only

Table S6: Patient satisfaction and blinding questions results summary

		PVB	TEB
Satisfaction with pain therapy after surgery			
Discharge (n=31 for PVB, n=32 for TEB)	Very satisfied	18 (58)	20 (63)
	Satisfied	10 (33)	9 (28)
	Dissatisfied	2 (6)	2 (6)
	Very dissatisfied	1 (3)	1 (3)
3 months (n=26 for PVB, n=23 for TEB)	Very satisfied	13 (50)	9 (39)
	Satisfied	13 (50)	8 (35)
	Dissatisfied	0 (-)	6 (26)
	Very dissatisfied	0 (-)	0 (-)
6 months (n=26 for PVB, n=25 for TEB)	Very satisfied	14 (54)	16 (64)
	Satisfied	11 (42)	6 (24)
	Dissatisfied	0 (-)	2 (8)
	Very dissatisfied	1 (4)	1 (4)
Satisfaction with care provided by hospital			
Discharge (n=31 for PVB, n=32 for TEB)	Very satisfied	22 (71)	22 (69)
	Satisfied	9 (29)	8 (25)
	Dissatisfied	0 (-)	2 (6)
	Very dissatisfied	0 (-)	0 (-)
3 months (n=26 for PVB, n=23 for TEB)	Very satisfied	15 (58)	12 (52)
	Satisfied	11 (42)	9 (39)
	Dissatisfied	0 (-)	2 (9)
	Very dissatisfied	0 (-)	0 (-)
6 months (n=26 for PVB, n=26 for TEB)	Very satisfied	19 (73)	15 (58)
	Satisfied	5 (19)	9 (35)
	Dissatisfied	2 (8)	2 (8)
	Very dissatisfied	0 (-)	0 (-)
Do you know which technique you received?			
Discharge (n=31 for PVB, n=32 for TEB)	TEB	1 (3)	19 (60)
	PVB	8 (26)	3 (9)
	Don't know	22 (71)	10 (31)
3 months (n=26 for PVB, n=22 for TEB)	TEB	2 (8)	12 (55)
	PVB	2 (8)	0 (-)
	Don't know	22 (84)	10 (45)
6 months (n=26 for PVB, n=25 for TEB)	TEB	2 (8)	13 (52)
	PVB	5 (19)	1 (4)
	Don't know	19 (73)	11 (44)

Frequency (%) presented (unless otherwise stated)

For peer review only - http://bmjopen.bmj.com/site/about/guidelines.xhtml

CONSORT STATEMENT – PILOT STUDY

Section/topic and item No	Standard checklist item	Extension for pilot trials	Page No where item is reported
Title and abstract			
1a	Identification as a randomised trial in the title	Identification as a pilot or feasibility randomised trial in the title	1
1b	Structured summary of trial design, methods, results, and conclusions (for specific guidance see CONSORT for abstracts)	Structured summary of pilot trial design, methods, results, and conclusions (for specific guidance see CONSORT abstract extension for pilot trials)	2
Introduction			
Background and objectives:			
2a	Scientific background and explanation of rationale	Scientific background and explanation of rationale for future definitive trial, and reasons for randomised pilot trial	4
2b	Specific objectives or hypotheses	Specific objectives or research questions for pilot trial	4
Methods			
Trial design:			
3a	Description of trial design (such as parallel, factorial) including allocation ratio	Description of pilot trial design (such as parallel, factorial) including allocation ratio	4
3b	Important changes to methods after trial commencement (such as eligibility criteria), with reasons	Important changes to methods after pilot trial commencement (such as eligibility criteria), with reasons	N/A

For peer review only

Participants:			
4a	Eligibility criteria for participants		5
4b	Settings and locations where the data were collected		4
4c		How participants were identified and consented	7
Interventions:			
5	The interventions for each group with sufficient details to allow replication, including how and when they were actually administered		5-6
Outcomes:			
6a	Completely defined prespecified primary and secondary outcome measures, including how and when they were assessed	Completely defined prespecified assessments or measurements to address each pilot trial objective specified in 2b, including how and when they were assessed	6
6b	Any changes to trial outcomes after the trial commenced, with reasons	Any changes to pilot trial assessments or measurements after the pilot trial commenced, with reasons	N/A
6c		If applicable, prespecified criteria used to judge whether, or how, to proceed with future definitive trial	6
Sample size:			
7a	How sample size was determined	Rationale for numbers in the pilot trial	6
7b	When applicable, explanation of any interim analyses and stopping guidelines		N/A
Randomisation:			
Sequence generation:			

For peer review only

8a	Method used to generate the random allocation sequence		5
8b	Type of randomisation; details of any restriction (such as blocking and block size)	Type of randomisation(s); details of any restriction (such as blocking and block size)	5
Allocation concealment mechanism:			
9	Mechanism used to implement the random allocation sequence (such as sequentially numbered containers), describing any steps taken to conceal the sequence until interventions were assigned		5
Implementation:			
10	Who generated the random allocation sequence, enrolled participants, and assigned participants to interventions		5
Blinding:			
11a	If done, who was blinded after assignment to interventions (eg, participants, care providers, those assessing outcomes) and how		6
11b	If relevant, description of the similarity of interventions		6
Analytical methods:			
12a	Statistical methods used to compare	Methods used to address each pilot trial objective whether	7

For peer review only

	groups for primary and secondary outcomes	qualitative or quantitative	
12b	Methods for additional analyses, such as subgroup analyses and adjusted analyses	Not applicable	N/A
Results			
Participant flow (a diagram is strongly recommended):			
13a	For each group, the numbers of participants who were randomly assigned, received intended treatment, and were analysed for the primary outcome	For each group, the numbers of participants who were approached and/or assessed for eligibility, randomly assigned, received intended treatment, and were assessed for each objective	Figure 1
13b	For each group, losses and exclusions after randomisation, together with reasons		Figure 1
Recruitment:			
14a	Dates defining the periods of recruitment and follow-up		8
14b	Why the trial ended or was stopped	Why the pilot trial ended or was stopped	N/A
Baseline data:			
15	A table showing baseline demographic and clinical characteristics for each group		Table 1
Numbers analysed:			

For peer review only

16	For each group, number of participants (denominator) included in each analysis and whether the analysis was by original assigned groups	For each objective, number of participants (denominator) included in each analysis. If relevant, these numbers should be by randomised group	Table 2
Outcomes and estimation:			
17a	For each primary and secondary outcome, results for each group, and the estimated effect size and its precision (such as 95% confidence interval)	For each objective, results including expressions of uncertainty (such as 95% confidence interval) for any estimates. If relevant, these results should be by randomised group	Table 3, 4
17b	For binary outcomes, presentation of both absolute and relative effect sizes is recommended	Not applicable	N/A
Ancillary analyses:			
18	Results of any other analyses performed, including subgroup analyses and adjusted analyses, distinguishing prespecified from exploratory	Results of any other analyses performed that could be used to inform the future definitive trial	N/A
Harms:			
19	All important harms or unintended effects in each group (for specific guidance see CONSORT for harms)		14
19a		If relevant, other important unintended consequences	N/A
Discussion			
Limitations:			

For peer review only

20	Trial limitations, addressing sources of potential bias, imprecision, and, if relevant, multiplicity of analyses	Pilot trial limitations, addressing sources of potential bias and remaining uncertainty about feasibility	14-17
Generalisability:			
21	Generalisability (external validity, applicability) of the trial findings	Generalisability (applicability) of pilot trial methods and findings to future definitive trial and other studies	14-17
Interpretation:			
22	Interpretation consistent with results, balancing benefits and harms, and considering other relevant evidence	Interpretation consistent with pilot trial objectives and findings, balancing potential benefits and harms, and considering other relevant evidence	14-15
22a		Implications for progression from pilot to future definitive trial, including any proposed amendments	15-17
Other information			
Registration:			
23	Registration number and name of trial registry	Registration number for pilot trial and name of trial registry	2
Protocol:			
24	Where the full trial protocol can be accessed, if available	Where the pilot trial protocol can be accessed, if available	N/A
Funding:			
25	Sources of funding and other support (such as supply of drugs), role of funders		17
26		Ethical approval or approval by research review committee, confirmed with reference number	5

For peer review only
